# Influence of the magnetic field on bandgap and chemical composition of zinc thin films prepared by sparking discharge process

**DOI:** 10.1038/s41598-020-58183-4

**Published:** 2020-01-29

**Authors:** Stefan Ručman, Panich Intra, E. Kantarak, W. Sroila, T. Kumpika, J. Jakmunee, W. Punyodom, Biljana Arsić, Pisith Singjai

**Affiliations:** 1 Material Science Research Center, Faculty of Science, Chiang Mai University, Chiang Mai, Thailand; 2 Research Unit of Applied Electric Field in Engineering (RUEE), College of Integrated Science and Technology, Rajamangala University of Technology Lanna, Chiang Mai, Thailand; 3 Department of Physics and Material Science, Faculty of Science, Chiang Mai University, Chiang Mai, Thailand; 4 Department of Chemistry, Faculty of Science, Chiang Mai University, Chiang Mai, Thailand; 5 Center of Excellence in Material Science and Technology, Chiang Mai University, Chiang Mai, Thailand; 6 Department of Mathematics, Faculty of Sciences and Mathematics, University of Nis, Nis, Republic of Serbia

**Keywords:** Solid-state chemistry, Synthesis and processing, Nanometrology

## Abstract

We examine the influence of the magnetic field on the chemical reaction of nitrogen and carbon dioxide in sparking electric discharge of zinc wires. Samples are prepared on Indium Tin Oxide (ITO) and quartz substrates in the form of thin films at 0 T, 0.2 T and 0.4 T. Different chemical composition of thin-films prepared by sparking discharge was obtained and verified by XPS, Raman and Cyclic voltammetry. Carbon dioxide conversion to carbonates was observed for zinc sparked in CO_2_ and nitrogen affecting crystallization of thin films was confirmed by XRD. Synthesis route for thin-film preparation used in this study is electric sparking discharge, convenient for fast ionization of metal and gasses. Band gap energy of thin films prepared by this method was starting from 2.81 eV and 4.24 eV, with the lowest band gaps prepared on ITO in 0.4 T. Dynamic mobility analysis (DMA) indicates smaller particles are fabricated by discharging zinc wires in a higher magnetic field. Nitridification of zinc nanoparticles occurred on 0.2 Tesla magnetic field strength and it was detectable even after XPS ion gun etching. Carbonation and nitridification of zinc thin films by sparking wires inside the magnetic field to observe the effect of the magnetic field on bandgap and chemical composition are confirmed by XPS.

## Introduction

Scientific community regards separately magnetic field influence *versus* electric field on chemical reaction. However, in our point of view these two-fields induce the same mechanisms in the chemical reaction. Based on the J. Howgego’s review^1^ oriented electric field influence the stability of charged transition states in molecules or atoms; the electric field has to be aligned with a particular axis of a molecule-electric field could influence the distribution of the electron density. The second group of researchers are those that investigate the magnetic field influence on chemical reactions. It was observed that magnetic field has an effect on absorption, crystallization, radicals pairing, triplet-triplet annihilation, protein crystallization, calcium carbonate crystallization or aragonite *versus* calcite formation. There are physically clear evidences of the fact that though the energy of magnetic interactions is small, under certain conditions relatively weak magnetic fields can noticeably affect the rates of chemical reactions^2^. Main underlying chemical principle is radical pair mechanism, where transition between triplet-singlet radical pair state is a tailored by discrete strength of magnetic field^3^. In our experimental design we use sparking discharge process as an ideal setup to conduct experiments in non-equilibrium conditions. Namely, the high voltage and low current across spark gap between tips of metal (Zinc) wires, enables surrounding that is full of exited molecules of gasses flowing across the sparking gap, and metal wires atoms that are melted by high voltage^4^. Voltage applied at the end of metal wire is large enough to melt it and break down CO_2_ or N_2_ that are present. Therefore, each molecule will be present in their radical or exited states which opens the opportunity for magnetic field to influence reaction products. Mikelson and Karklin^5^, solidified metal alloys melt in magnetic field from 0.5 T to 1.5 T and they found that primary nuclei of the alloy crystalized, were oriented with their longer axes along the lines of force of the magnetic field. They explain crystal orientation effect of magnetic field as depending on magnetic properties of crystal nucleus and those of melts. In their opinion, homogeneity of magnetic field also played an important role in the crystal orientation. Next emphasis of their work is on the electron flow generation if the melt which crystalizes is conductive when cooling down heterogeneity by phase transition will arise. The last emphasis was the influence of applied magnetic field on thermal convection which will define final crystal by influencing viscosity of the melt. Regarding inorganic molecules, there are two direactions examining effect of magnetic field on crystallization, one in thermal processing/analysis^6^ and other in solution chemistry^7^ (sedimentation experiments). In the first case, the high magnetic field (up to 20 T) was introduced in high temperature environments in order to control material functionality. We can observe two phenomena regarding this case: A) effect on phase transitions and B) an increase in coercivity of permanent magnets. Iron phase in multicomponent crystalline alloys will increase in the nucleation rate of iron by annealing in magnetic fields^8^, also using spark discharge inside of pure nitrogen atmosphere where will be different products of nitride obtained^4^ or with field above 0.4 T as a product will be created pure zerovalent metals^9^ Regarding solution chemistry, precipitation of 90% of CaCO_3_ in 1.2 T external magnetic field resulted in sedimentation of aragonite/vaterite crystals. The externally applied magnetic field is lowering the zeta potential of CaCO_3_^10^. Aragonite is formed at high temperature and pressure from melts. Therefore, the formation of calcite is energetically in favor. However, why in applied external field, aragonite is formed over calcite? Theoretically, it can happen only if magnetic field is 45 T, but still researchers achieve it under 0.4 T; with the high gain of aragonite concentration is achieved (80%)^11^. In our work sparking discharge method produce thin films inside magnetic field on different substrates, with focus on chemical reaction that is occurring in the region of zinc wire electrical discharge during breakdown voltage of gases (CO_2_ and N_2_) under magnetic field of permanent magnet that is not uniform. Such chemical reaction fabricates nanoparticles which morphology, crystallinity and chemistry depends on composition of carrier gas, magnetic field and type of substrate used for nanoparticles deposition.

## Results

**Figure 1:**
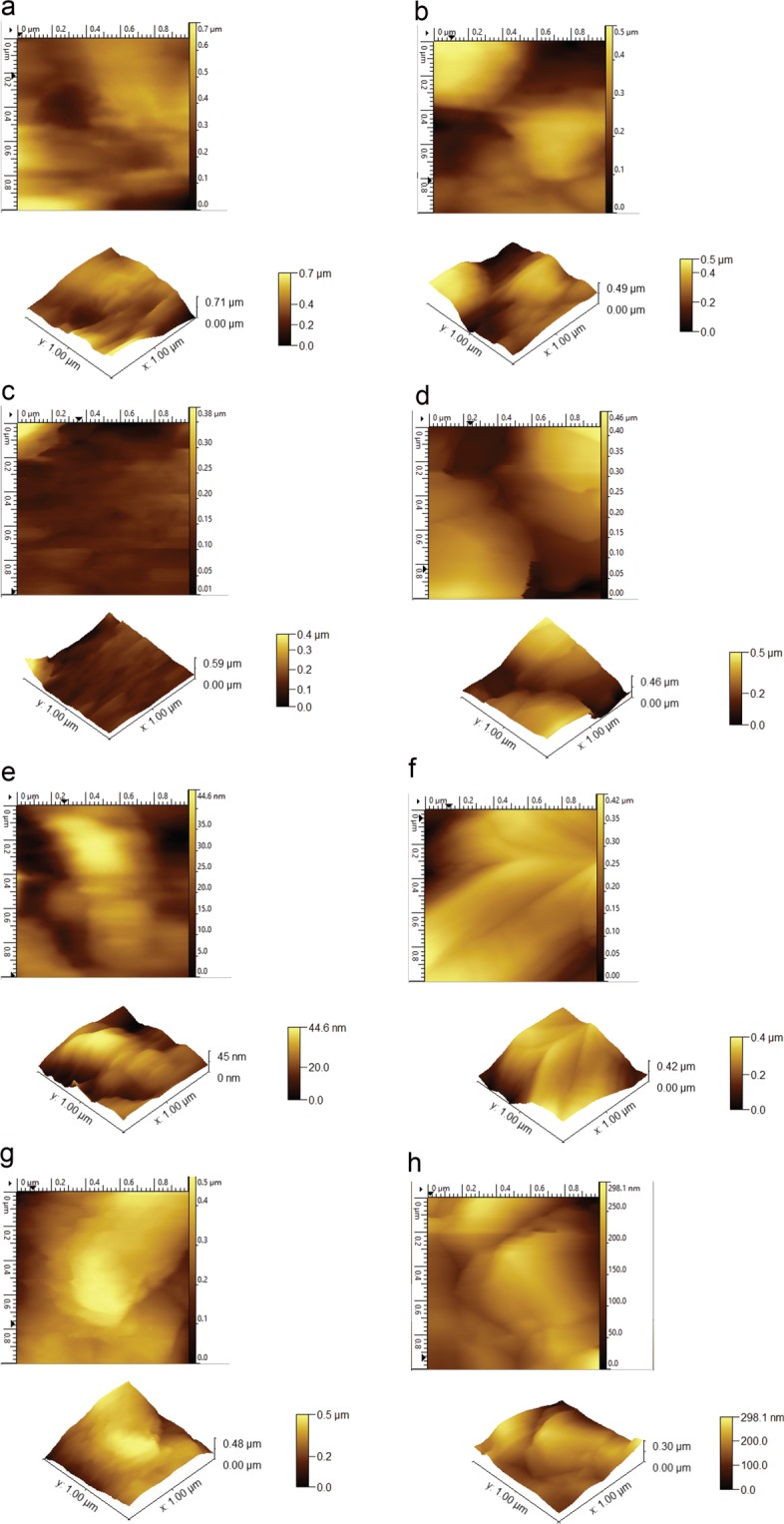
(**a**) Atomic Force Microscopy, non-tapping mode, sample of Zinc deposited on quartz, prepared in 0.2 T magnetic field in the flow of CO_2_ resulting in average value of thin film 385.479 nm; RMS roughness (Sq and grain-wise): 110.594 nm with mean roughness (Sa): 90.453 nm. (**b)** Zinc deposited on quartz, 0.2 T in the N_2_ flow resulting in average value of thin film 260.411 nm; RMS roughness (Sq and grain-wise): 107.752 nm with mean roughness (Sa): 87.033 nm. (**c**) Zinc deposited on quartz, 0.4 T in the flow of CO_2_ resulting in average value of thin film 136.673 nm; RMS roughness (Sq and grain-wise): 40.4048 nm with mean roughness (Sa): 24.5386 nm. (**d**) Zinc deposited on ITO, 0.4 T in the flow of CO_2_ resulting in average value of thin film 232.204 nm; RMS roughness (Sq and grain-wise): 97.1808 nm with mean roughness (Sa): 82.6234 nm. (**e**) Zinc deposited on ITO, 0.4 T in the flow of N_2_, average value of thin film 22.1103 nm; RMS roughness (Sq and grain-wise): 9.34226 nm with mean roughness (Sa): 7.48186 nm. (**f**) Zinc deposited on ITO, 0.2 T in the flow of CO_2_ resulting in average value of thin film 274.252 nm; RMS roughness (Sq and grain-wise): 73.6122 nm with mean roughness (Sa): 56.5023 nm. (**g**) Zinc deposited on quartz, 0.4 T in the flow of N_2_ resulting in average value of thin film 320.978 nm; RMS roughness (Sq and grain-wise): 84.3617 nm with mean roughness (Sa): 68.2234 nm. (**h**) Zinc deposited on ITO, 0.2 T in the flow of N_2_ resulting in average value of thin film 175.142 nm; RMS roughness (Sq and grain-wise): 43.8872 nm with mean roughness (Sa): 35.7607 nm.

**Figure 2:**
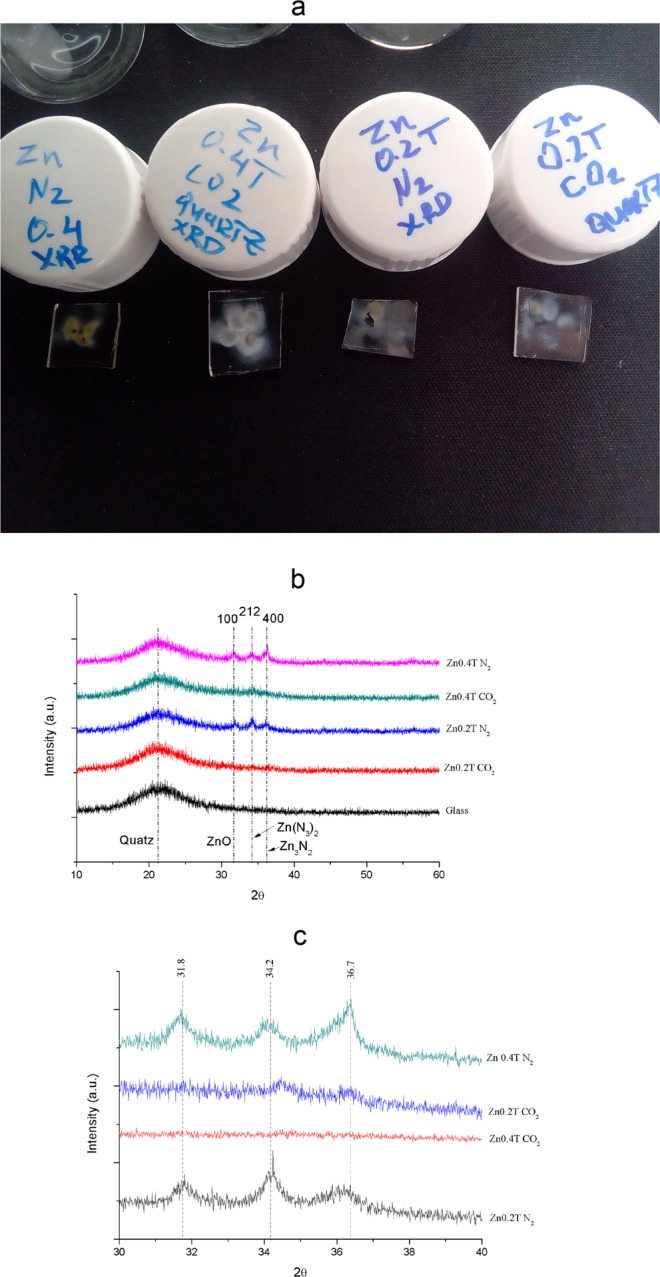
(**a**) Real life picture representation of Zinc thin films deposited on quartz substrate for GIXRD analysis, from left to right first is Zn sparked inside of 0.4 T under N_2_ conditions, second in CO_2_; third is Zinc sparked inside of 0.2 T under Nitrogen and last one under CO_2_. (**b**) XRD diffractograms of thin films from Fig. 2a. (**c**) Close up scan of observed peaks. Nitrogen plays important role in crystallization of thin films at ambient temperature.

**Figure 3:**
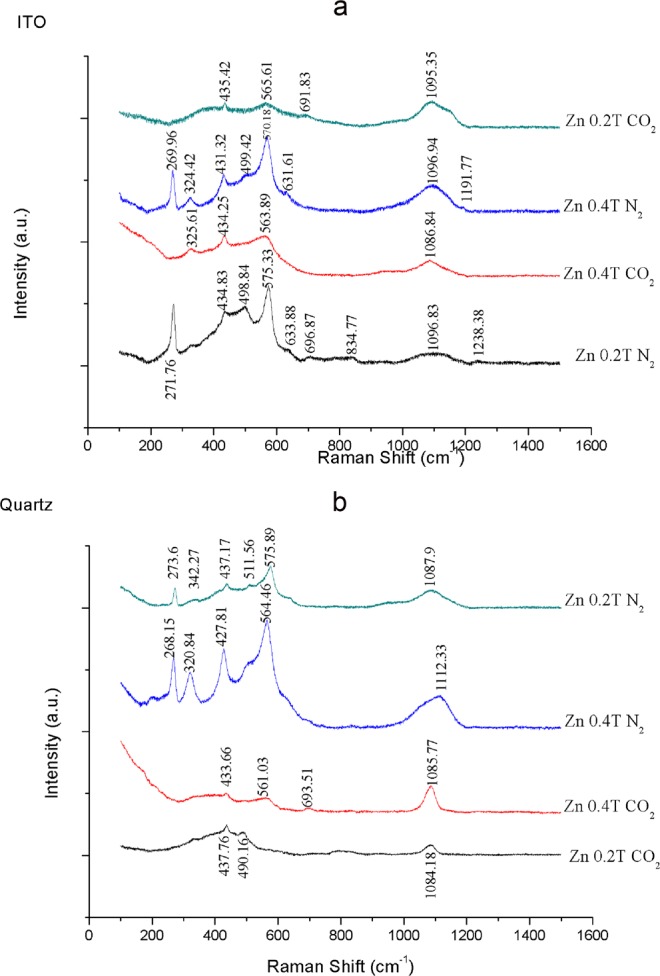
(**a**) Raman spectroscopy of substrate deposited on ITO substrate at different magnetic field and different atmosphere; (**b**) Raman spectroscopy of substrate deposited on quartz at different magnetic field and carrier gasses.

**Figure 4:**
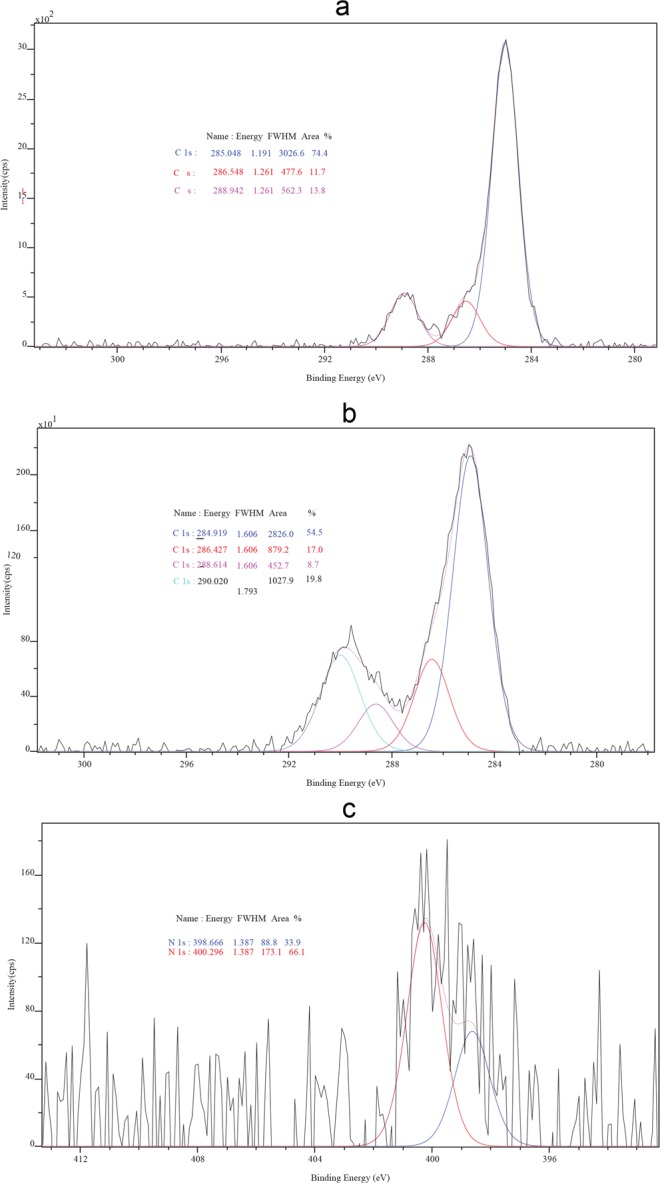
(**a**) Etched thin film substrate prepared by sparking zinc wire in 0.4 T on ITO under nitrogen atmosphere. C 1 s binding energy (**b**) C 1 s XPS profile of Zinc sparked in CO_2_, 0.4 T on ITO. Distinguishable difference from (a). (**c**) Etched by XPS beam thin film prepared at 0.2 T in nitrogen atmosphere on quartz. Zinc nitride peak on 398.666 and 400.296, results not represented in Table 2.

**Figure 5:**
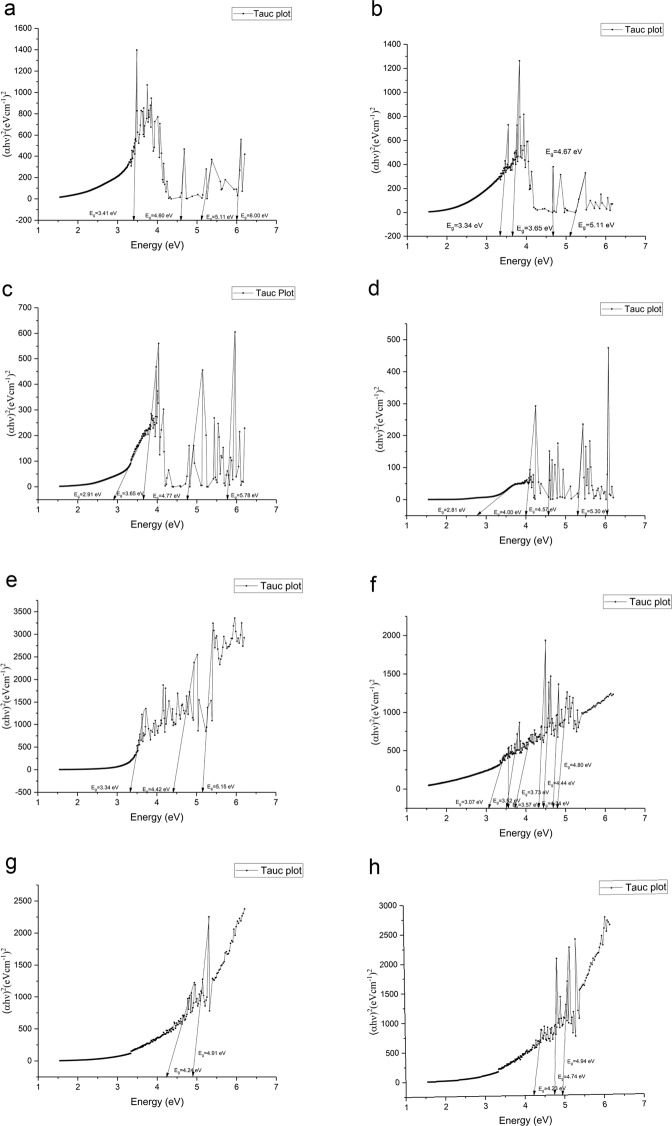
(**a**) Zinc thin film, wire sparked in nitrogen,0.2 T on ITO. (**b)** Zinc thin film, wire sparked in carbon dioxide, 0.2 T on ITO. (**c**) Zinc thin film, wire sparked in carbon dioxide, 0.4 T; on ITO (**d**) Zinc thin film, wire sparked in nitrogen, 0.4 T; on ITO (**e**) Zinc thin film, wire sparked in nitrogen, 0.4 T on quartz substrate. (**f**) Zinc thin film, wire sparked in carbon dioxide, 0.4 T on quartz substrate. (**g**) Zinc thin film, wire sparked in nitrogen, 0.2 T on quartz substrate (**h**) Zinc thin film, wire sparked in carbon dioxide, 0.2 T on quartz substrate.

**Figure 6:**
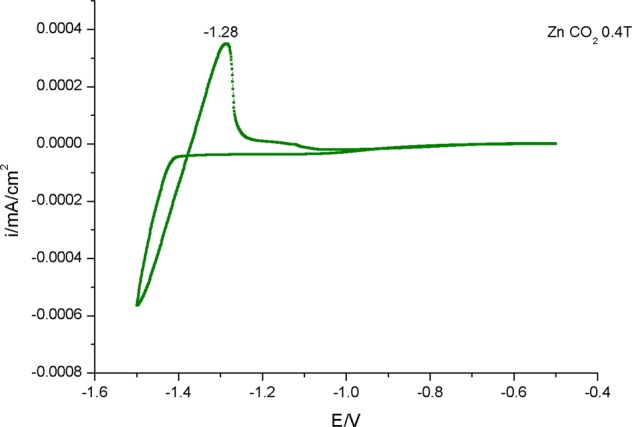
Cyclic voltammogram of thin film prepared in carbon dioxide, at 0.4 T on ITO.

**Figure 7:**
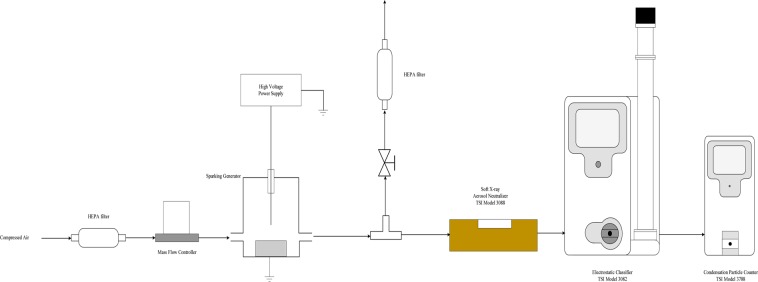
Compressed air 10 L/s flown trough HEPA filter, mass flow controller that direct to inlet of sparking machine chamber, outlet is changing to control filter in two direction one in DMA TSI Model apparatus and second one to regulate flow in first.

Sparking discharge process usually uses high voltage to facilitate transfer of matter in the medium between tips of the wires. If this medium is a gas, due to abrasion of an electrode (metal wire) with high voltage, highly charged particles are released in the form of aerosols and these particles can be collected on any type of substrate. To investigate nanoparticle distribution of aerosols created by sparking discharge and influence of magnetic field on median aerodynamic diameter we employed dynamic mobility analyzer (DMA), measured at different applied voltage and with and without presence of magnetic field. Results are presented in Table 1. After that we focus on the characterization of surface morphology with AFM (Fig. 1) and crystallization effect of substrate, carrier gas and different magnetic field by GI-XRD (Fig. 2), following with chemical characterization of thin film surface by RAMAN (Fig. 3) and XPS (Table 2 and Fig. 4) to determinate effect of substrate on a composition. Also, we focus on magnetic field influence on chemical composition as well as different discharge gases used as electron carrier medium between two electrodes (Zinc metal wire). Carbon dioxide (CO_2_) and nitrogen (N_2_) were used as carrier gases in which Zinc wires were discharged; using XPS surface etching we determined how deep inside of thin film substrate are these gases incorporated or if their effect is superficial to zinc oxidation, nitridification or carbonation. The effect of magnetic field and type of substrate used for nanoparticle deposition is observed and they are both investigated for influence on band gap using Tauc plot, characterization with UV-Vis (Fig. 5); substrate prepared on ITO is further examined with Cyclic Voltammetry in 0.1 M KOH (Fig. 6).Schematic representation of experimental setup connected to DMA, as well as photographs of sparking machine and chamber are represented at Fig. 7 and explained in Methods section.

**Table 1:** Comparison of results obtained from dynamic mobility analyzer (DMA) by sparking Zinc wire at different conditions. Geometric mean as average particle size, concentration in number of particles per cm^3^.

Zinc wire sparked inside of 0.4 T. In the gas flow	energy applied across the gap; kV/A	2.13/0.12	3.36/0.36	4.68/0.52
	Average number particle size; nm	41.1	56.2	66.8
	concentration	3.65*10^6^	9.98*10^6^	1.34*10^7^
	Geometric SD	1.93	1.79	1.73
Zinc wire sparked inside of 0 T. In the gas flow	energy applied across the gap; kV/A	2.15/0.13	3.38/0.36	4.64/0.52
	Average number particle size; nm	38.1	54.9	66.2
	concentration	2.01*10^6^	8.25*10^6^	1.14*10^7^
	Geometric SD	1.93	1.80	1.72
Zinc wire sparked inside of 0 T with no gas flow	energy applied across the gap; kV/A	2.13/0.12	3.36/0.36	4.64/0.55
	Average number particle size; nm	135	181.3	145.7
	concentration	5.27*10^5^	1.57*10^6^	5.92*10^5^
	Geometric SD	1.94	1.81	1.85
Double head sparked zinc in 0 T, with air flow	energy applied across the gap; kV/A	1) 4.58/2.76 2) 4.62/0.54	Double head sparked zinc in 0 T, no air flow	1) 4.58/2.76 2) 4.62/0.54
	Average number particle size; nm	72.2		192.9
	concentration	1.41*10^7^		5.53*10^5^
	Geometric SD	1.71		1.60

**Table 2:** XPS results of thin films prepared on different substrates, under different atmosphere and magnetic field.

	Zn 2p	O 1 s	N 1 s	C 1 s
N_2_; 0.2 T; ITO;	1021.543/1044.615	530.284/531.479/532.275/533.444	—	285.034/286.382/288.893
CO_2_; 0.2 T; ITO	1022.102/1045.146	530.085/531.304/532.100/533.025/534.276	—	284.972/286.455/288.781/289.927
CO_2_; 0.4 T; ITO	1021.281/1044.325	529.873/530.945/532.017/533.089/534.372	—	284.919/286.427/288.614/290.020
N_2_; 0.4 T; ITO	1021.572/1044.639	530.233/531.428/532.203	—	285.001/286.484/288.776
N_2_; 0.4 T; Quartz	1021.391/1044.496	530.188/531.609/532.433	—	284.933/286.449/288.691
CO_2_; 0.4 T, Quartz	1022.127/1045.165	530.058/531.309/532.105/533.242	—	285.037/286.503/288.762/289.908
N_2_; 0.2 T; quartz	1021.564/1044.652	530.123/531.318/532.142/533.579	400.074	284.983/286.034/287.153/288.949
CO_2_; 0.2 T; quartz	1022.169/1045.244	530.485/531.931/532.841	—	285.052/286.552/288.810/289.973

## Particle distribution (DMA)

Table 1 Comparison of results obtained from dynamic mobility analyzer (DMA) by sparking Zinc wire at different conditions. Geometric mean as average particle size, concentration in number of particles per cm3

According to the values represented in Table 1, Zinc wire sparked in 0.4 T magnetic field has lower average size and higher concentration of produced aerosol particles. These results point out that magnetic field stabilizes ionized zinc particles and increase frequency of sparked wire. Furthermore, gas flow plays an important role in particle distribution which is in accordance with previous research^12^.

Further analysis of aerosol deposited on glass substrate with Atomic Force Microscopy (non-contact mode) provide us with information of average roughness approximately 273.821 nm ± 37.404 at 0.2 T, with lower roughness when magnetic field is 0.4 T of the value 177.99 nm ± 55.56. Which is in accordance with DMA results that are providing information about magnetic field influence on nanoparticle size and concentration. At Fig. 1. results are presented from AFM of zinc particles deposited on different substrates in 0.2 T or 0.4 T nonuniform field in carbon dioxide or nitrogen flow.

Concentric pattern on AFM image is assumed to correspond to direction of magnetic field from permanent magnet that is used to create nonuniform magnetic field. As shown on Fig. 2a, we can observe agglomeration and circular pattering on glass substrate. These substrates are then used for characterization of XRD diffraction peaks, represented on Fig. 2b,c.

GI-XRD provides information that Nitrogen atmosphere enhances crystallization of thin films, which is in accordance with previous research work^13^. Diffraction peaks found are identified as Zinc oxide and Zinc nitride phases. They were matched in JSPDF database as belonging to (100) at 31.8° (00-005-0664 from ZnO), (212) at 34.2^o^ (00-023-0740 from Zn(N_3_)_2_) and (400) at 36.7^o^ (00-035-0762 from Zn_3_N_2_); GI-XRD data are showed on Fig. 2b,c and are followed with RAMAN and XPS characterization in order to determinate influence of nitrogen and carbon dioxide on chemical composition of films.

Results indicates that sparking in high purity nitrogen atmosphere crystalize thin-films. Magnetic field utilization during electrical spark discharge fabricates nanoparticles that are smaller in diameter size and higher in particle concentration.

### Chemical composition

For the reason of clarity, substrates deposited on ITO and quartz are represented in separated graphs. There is overwhelming evidence that zinc deposited on ITO provides different RAMAN shift comparing with quartz as well difference in thin films prepared by sparking in CO_2_ and N_2_.

Substrates prepared in nitrogen gas are polymorph substantially in comparison with thin films synthetized in carbon dioxide, that also exhibit lower peak intensity probably because lower crystalinity which is comfirmed with GI-XRD as well as later with bandgap estimation^14^. Characteristic properties of Raman scattering from thin-films prepared by sparking in carbon dioxide is peak around 435 cm^−1^ and weak influence of both substrates on spectra. While substrate influence can be observed for thin films prepared by sparking inside of nitrogen atmosphere. Raman peak of ZnO bulk crystal which also represents the band characteristic of the wurtzite phase is on mode at 436.16 cm^−1^^15^; main Raman signature for zinc carbonate is 1090 cm^−1^ approximetly^16^. Futher investigation of thin films with XPS provide us with unique binding energy information about carbon (C 1 s) on 290 eV for substrate prepared in CO_2_ corresponding to metal carbonates, as shown in Fig. 4 and in Table 2. Nitrogen binding energy was observed with thin films prepared on quartz and at 0.2 T inside of nitrogen atmosphere. Binding energy was persistant at 398.6 even afer etching of substrate with ion beam of XPS, as represented at Fig. 4c. XPS shows an evidence of nitridification and carbonation of Zinc thin-films. What is possible to conclude is that sparking during the flow of ultrapure Nitrogen gas will allow incorporation of excited nitrogen species only in one particular range of 0.2 T, which is also shown in our previously published experiment with Iron^4^. When Carbon dioxide was used as a carrier gas in sparking chamber, there is no influence on chemical reaction. It means that high voltage which was applied across sparking gap even if molecules of CO_2_ were excited and ionized, it didn’t provide condition for the magnetic field to influence thin film crystallization and chemical reaction of Carbonate (ionized carbon dioxide), providing us with conclusion to believe that in CaCO_3_ weak magnetic force reactor Calcium have a greater role in conversion of calcite into aragonite^17^.

### Band gap

Characterization by optical band gap was done for elucidating heterogeneity by crystallinities and chemical composition of thin films prepared by sparking process under different dielectric gases used during sparking discharge process, with no thermal annealing applied under only magnetic field of 0.2 T and 0.4 T. Additionally substrates prepared on ITO were characterized by Cyclic Voltammetry. Tauc plots are represented at Fig. 5.

Thin films prepared in higher magnetic field 0.4 T have lower band gap energies than the one prepared in 0.2 T, which is related to the size of zinc nanoparticles produced by spark discharge as shown in Table 1. Size of nanoparticles decreases with increase in magnetic field. Using permanent magnet that doesn’t generate uniform magnetic field will create polycrystalline structures that will consequently provide more than one band gap present. This polycrystallinity is observed in above mentioned AFM and XRD results. There are two explanations of such a different and various band gap values. First one is due to incomplete coverage of thin film on substrate and heterogenicity as observed by AFM results, which is in accordance with already known effect of film thickness on the energy band gap^18^. There is a strong dependence between band gap and optical properties due to carrier confinement if one decreases the thickness of the thin film. The second important explanation of different band gap results is that thin films prepared in this experiment are not from the same compound, as shown by the XPS and Raman, even if we used ultrapure gas to spark Zinc wire, we still get zerovalent Zinc, Zinc oxide and zinc nitride/carbonate. Oxidation, effect of substrate, inefficient dielectric gas discharge and uneven carbonation/nitridification results in the different nanoparticles produced by the sparking discharge process, as seen by different colors of thin films in Fig. 2a and several band gaps detected in one sample^19^.

Thin films prepared on ITO have considerably lower band gap of sparked zinc thin films, to investigate this further we characterized them by cyclic voltammetry. Result show in Fig. 6, of thin film prepared in 0.4 T under carbon dioxide condition on ITO, other thin films deviate slightly from observed peak.

Aerosol-generated metal nanoparticles can oxidize during particle formation due to impurities in the carrier gas. One method to obtain high crystallinity and avoid thermal annealing or sintering is sparking inside magnetic field^4,9^. As shown in our previous work there is an influence on the crystallinity of nanoparticles when they are prepared in the magnetic field. Three possible reasons, either Lorentz force, magnetocrystalline anisotropy or effect of magnetic field on radical pairing^20–22^.

In our experiment we used DMA to show the difference in the distribution of aerosol generated particles inside and outside magnetic field, when sparked discharged Zinc wires were inside magnetic field. Different nanoparticle size influence grain size of thin film which influence band gap as explained previously.

## Discussion

Spark discharge process or spark ablation is electrical process for synthesis of aerosols that are composed of nanoparticles discharged in carrier gas, flow of the gas will influence particle size and have a purpose as dielectric material. In our study we deposited zinc thin films on two types of substrate materials, one is ITO and second quartz substrate. When the optical direct band gap was calculated there is difference in energy for 0.2–0.4 eV higher for substrates prepared on quartz. Influence of substrate on electronic properties of thin films is previously reported^23^. Effect of substrate for thin films needs to be take into consideration. There is better nucleation of primary aerosol nanoparticles on quartz, in terms of lattice compatibility and less strain on Zn films from quartz, than ITO. For compressive (negative) strain, the band gap should increase which can be concluded from AFM surface morphology and XRD results of thin films deposited on quartz^24^. If the substrate is more crystalline, the overgrown film has a higher band gap^25^.

Sparking inside of ultrapure nitrogen atmosphere enhances crystallization due to incorporation of nitrogen in the thin film^4,26–28^. Since the thin films prepared in this work were not annealed, we would like to know what role magnetic field has in nitride formation, as observed with sample prepared in 0.2 T and on quartz and why it hasn’t influenced carbonization or conversion of CO_2_ in Zinc Carbonate.

High power nitrogen laser emits light when excited with high voltage^29–31^. This excitation induces nitrogen to be in triplet or unstable state, which is susceptible to influence of the magnetic field if two radicals, or ionized molecules/atoms are in close proximity, the magnetic field in that example will influence on triplet-singlet crossing. At 0.4 T nitrogen is excluded from bonding with zinc, however this higher field will create smaller particles and thus lower optical band gap. Since sparking discharge process utilizes a high voltage to evaporate the zinc wire, knowing the principle of nitrogen laser, on the tips of wire there will be metal melt and high voltage through spark gap will turn carrier gases into plasma or into some other excited state. In comparison of dissociation energy between CO_2_ and N_2_^32^, we can conclude that dissociation energy of the CO_2_ is higher than the N_2_ at constant temperature and nitrogen can create highly active plasma environment than CO_2_. In such an active environment the zinc vapor will take a longer time to settle and form a crystal, this will consequently lower the speed of crystallization processes which will give higher crystallinity^33^.

## Methods

Zinc wire, 99.9% of purity ordered from ADVENT. Quartz and ITO substrate from NANOCS. The substrates were sonically cleaned in acetone, distilled water, and ethanol, and then dried by nitrogen gas blowing. The two sharp tips were prepared from the zinc wire (Ø 0.38 mm, purity 99.97%, Advent Research Materials Ltd). The tips were then placed horizontally at 3 mm spacing and 2 mm above the center of the substrate. The sparking occurred as in conditions described in our previous work^34,35^. The experiment was done repeatedly 150–200 times in ultrapure nitrogen and carbon dioxide gas flow (300 psi) at pressure slightly higher inside of sparking head box in order to prevent contamination with oxygen. 0.4 Tesla high temperature Neodymium magnet was obtained from Ningbo Risheng Magnets Co., Ltd. (Ningbo, China). 0.2 Tesla magnet was made from stacking discoid plates permanent magnets. The surface morphology, root-mean-square (rms) roughness, and film thickness were characterized by atomic force microscopy. AFM measurements were performed by a XE 70 model (Park system, Korea) in contact mode with NCS36 cantilevers, with tip apex radius of curvature under 10 nm, the scan rate of 1 Hz and scan area 1*1 μm^2^, to study surface morphology. The Raman spectra were obtained with a 514.5 nm argon ion laser at room temperature (Jobin Yvon Horiba T64000). The oxidation states and chemical composition of elements were analyzed by XPS (AXIS Ultra DLD, Kratos Analytical Ltd.). Optical transmittances were carried out in the range of 200 to 1100 nm using UV–vis spectrophotometer (Varian Carry 50 C). For thin film characterization, Rigaku Smartlab XRD (Rigaku, Tokyo, Japan) with Rigaku’s PDXL software for X-ray analysis was used with Cu source (1.541862 A) and D/teX Ultra 250 detectors. Cyclic voltammetry. A µAutolab type II (Metrohm, Netherland) was used for conducting electrochemical measurements of cyclic voltammetry. The three-electrode system consists of a thin film sample on substrate on a clamp as a working electrode, a Ag/AgCl as a reference electrode and a platinum rod as an auxiliary electrode. In Fig. 7 schematically is represented experimental setup of sparking machine connected to Dynamic Mobility Analyzer (DMA), operating principle was explained previously^36,37^.

## Supplementary information

Related Manuscript File.

Related Manuscript File.

Related Manuscript File.

Related Manuscript File.

Related Manuscript File.

Related Manuscript File.

transmitance of thin films prepared on ITO.

transmitance of thin films prepared on Quartz.
