# Author Correction: Influence of the magnetic field on bandgap and chemical composition of zinc thin films prepared by sparking discharge process

**DOI:** 10.1038/s41598-020-61009-y

**Published:** 2020-03-10

**Authors:** Stefan Ručman, Panich Intra, E. Kantarak, W. Sroila, T. Kumpika, J. Jakmunee, W. Punyodom, Biljana Arsić, Pisith Singjai

**Affiliations:** 1 Material Science Research Center, Faculty of Science, Chiang Mai University, Chiang Mai, Thailand; 2 Research Unit of Applied Electric Field in Engineering (RUEE), College of Integrated Science and Technology, Rajamangala University of Technology Lanna, Chiang Mai, Thailand; 3 Department of Physics and Material Science, Faculty of Science, Chiang Mai University, Chiang Mai, Thailand; 4 Department of Chemistry, Faculty of Science, Chiang Mai University, Chiang Mai, Thailand; 5 Center of Excellence in Material Science and Technology, Chiang Mai University, Chiang Mai, Thailand; 6 Department of Mathematics, Faculty of Sciences and Mathematics, University of Nis, Nis, Serbia

Correction to: *Scientific Reports* 10.1038/s41598-020-58183-4, published online 29 January 2020

The original version of this Article contained a repeated typographical error where “Differential Mobility Analyzer” and “Differential Mobility Analysis” were given as “dynamic mobility analyzer” and “dynamic mobility analysis” respectively.

In the Abstract,

“Dynamic mobility analysis (DMA) indicates smaller particles are fabricated by discharging zinc wires in a higher magnetic field.”

now reads:

“Differential Mobility Analysis (DMA) indicates smaller particles are fabricated by discharging zinc wires in a higher magnetic field.”

In the Results section,

“To investigate nanoparticle distribution of aerosols created by sparking discharge and influence of magnetic field on median aerodynamic diameter we employed dynamic mobility analyzer (DMA), measured at different applied voltage and with and without presence of magnetic field.”

now reads:

“To investigate nanoparticle distribution of aerosols created by sparking discharge and influence of magnetic field on median aerodynamic diameter we employed Differential Mobility Analyzer (DMA), measured at different applied voltage and with and without presence of magnetic field.”

In the Methods section,

“In Fig. 7 schematically is represented experimental setup of sparking machine connected to Dynamic Mobility Analyzer (DMA), operating principle was explained previously^36,37^.”,

now reads:

“In Fig. 7 schematically is represented experimental setup of sparking machine connected to Differential Mobility Analyzer (DMA), operating principle was explained previously^36,37^.”

In the legend of Table 1, where,

“Table 1. Comparison of results obtained from dynamic mobility analyzer (DMA) by sparking Zinc wire at different conditions. Geometric mean as average particle size, concentration in number of particles per cm^3^.”

now reads:

“Table 1. Comparison of results obtained from Differential Mobility Analyzer (DMA) by sparking Zinc wire at different conditions. Geometric mean as average particle size, concentration in number of particles per cm^3^.”

Additionally, the first two sentences in the Particle distribution (DMA) section are a duplication of the legend of Table 1. The text,

“Table 1. Comparison of results obtained from dynamic mobility analyzer (DMA) by sparking Zinc wire at different conditions. Geometric mean as average particle size, concentration in number of particles per cm^3^.”, has been removed from this section.

These errors have now been corrected in the PDF and HTML versions of the Article.

