# Supplementary material for: Influence of the magnetic field on bandgap and chemical composition of zinc thin films prepared by sparking discharge process
Source: Sci Rep. 2020 Jan 29;10:1388. doi: 10.1038/s41598-020-58183-4 (PMC6989455; doi:10.1038/s41598-020-58183-4)
Supplement: Supplementary file 1 — Related Manuscript File. [file 41598_2020_58183_MOESM1_ESM.zip › Zn_AFM_241262.pdf]

1) Zn_N2

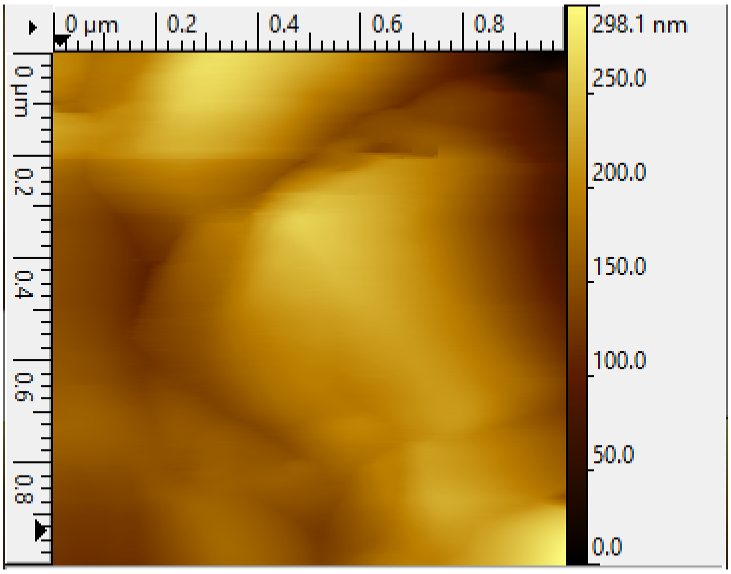

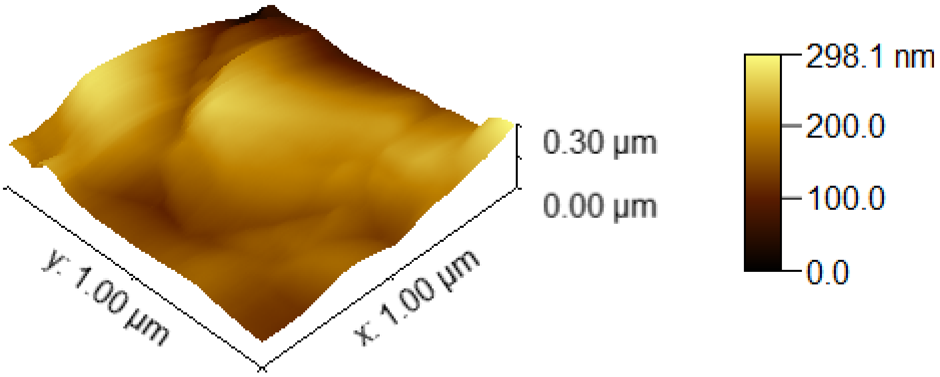

Statistical Quantities

Average value: 175.142 nm

RMS roughness (Sq): 43.8872 nm

RMS (grain-wise): 43.8872 nm

Mean roughness (Sa): 35.7607 nm

Skew (Ssk): -0.201434

Kurtosis: 0.119204

2) Zn_N2_0.4_XRD

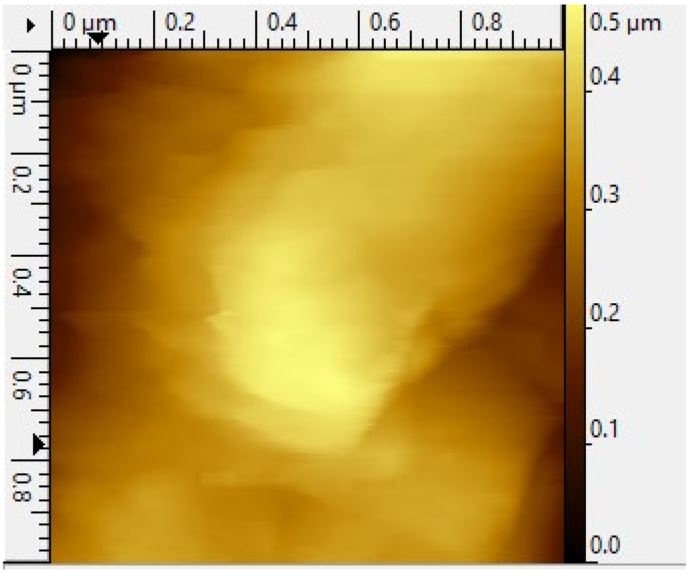

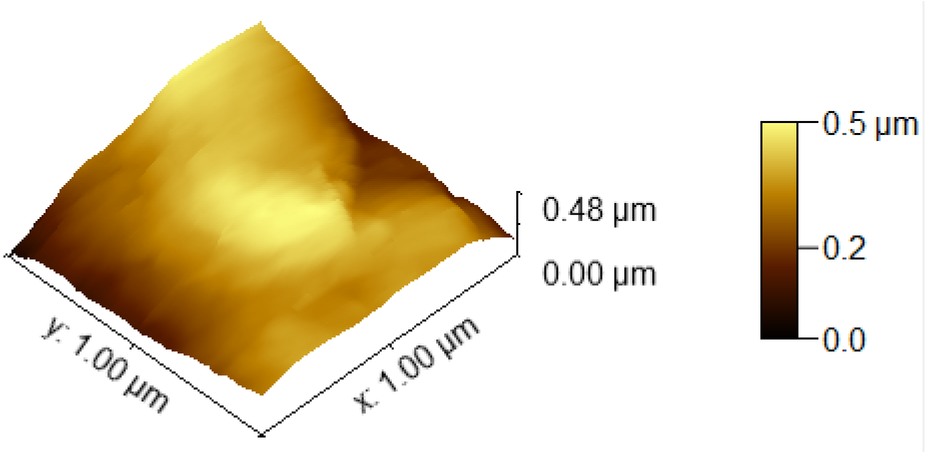

Statistical Quantities

Average value: 320.978 nm

RMS roughness (Sq): 84.3617 nm

RMS (grain-wise): 84.3617 nm

Mean roughness (Sa): 68.2234 nm

Skew (Ssk): -0.515569

Kurtosis: -0.313578

3) Zn_CO2

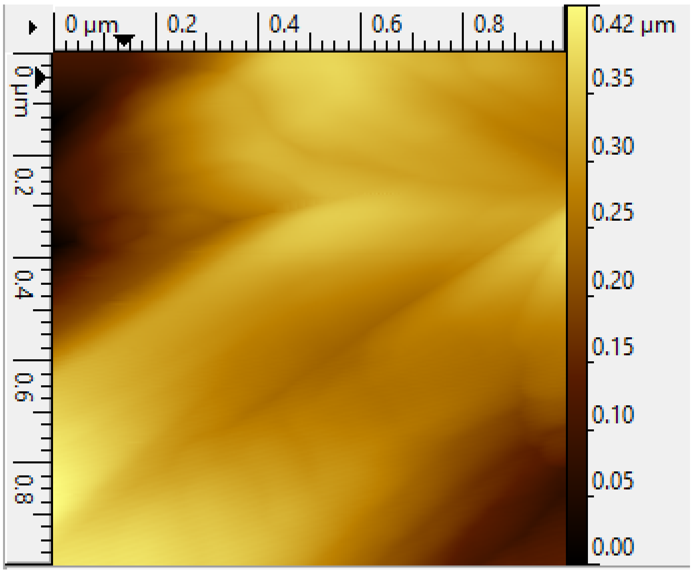

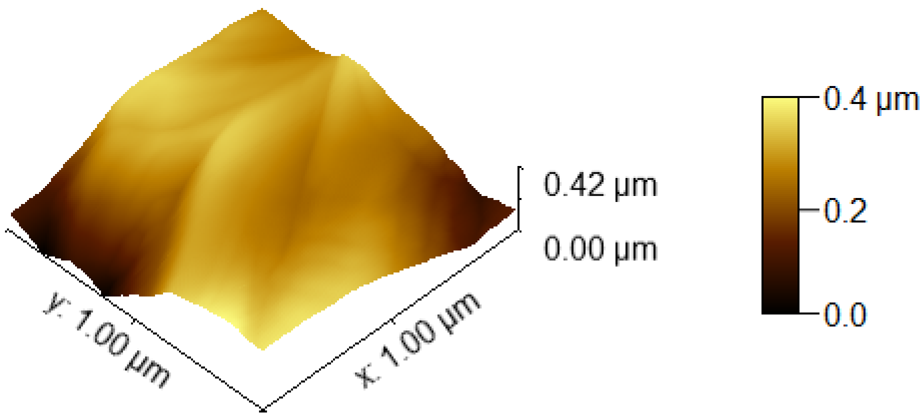

Statistical Quantities

Average value: 274.252 nm

RMS roughness (Sq): 73.6122 nm

RMS (grain-wise): 73.6122 nm

Mean roughness (Sa): 56.5023 nm

Skew (Ssk): -1.05178

Kurtosis: 0.720727

4) Zn_0.4T_N2

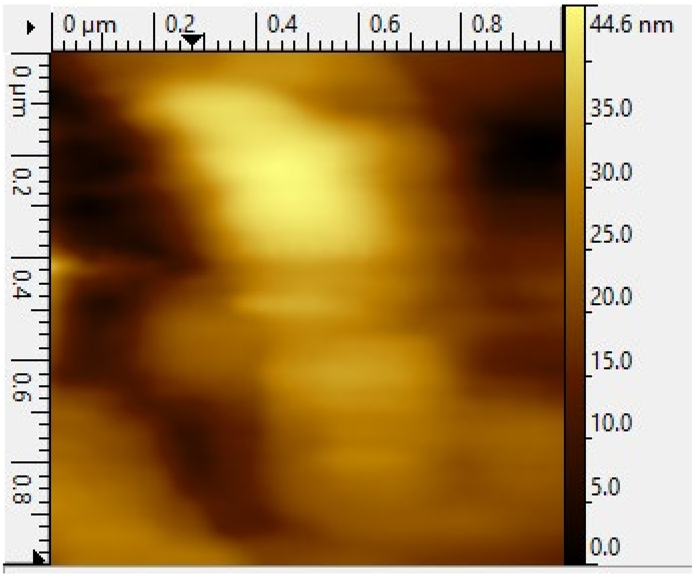

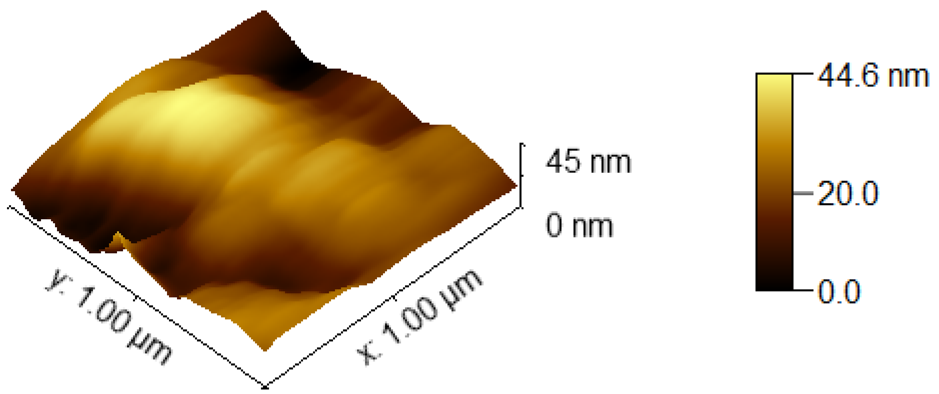

Statistical Quantities

Average value: 22.1103 nm

RMS roughness (Sq): 9.34226 nm

RMS (grain-wise): 9.34226 nm

Mean roughness (Sa): 7.48186 nm

Skew (Ssk): 0.0710322

Kurtosis: -0.345749

5) Zn_0.4T_CO2

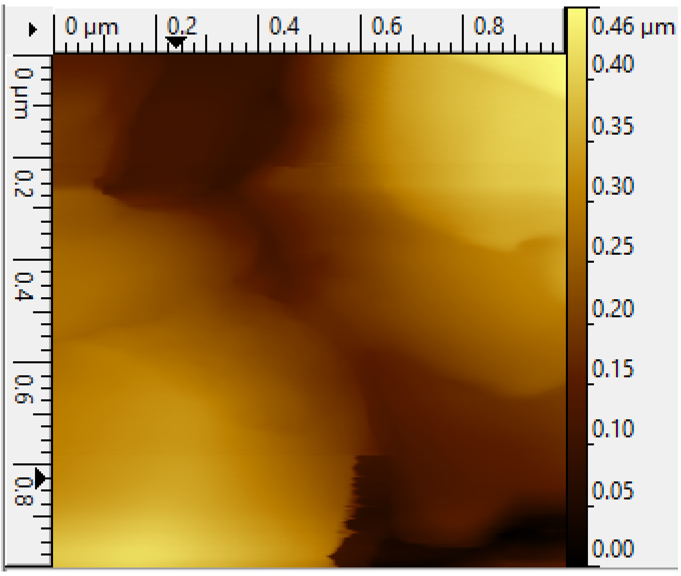

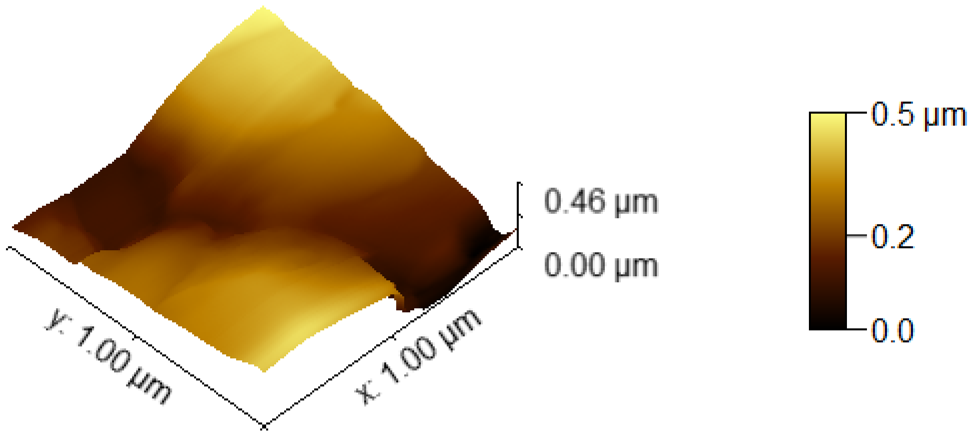

Statistical Quantities

Average value: 232.204 nm

RMS roughness (Sq): 97.1808 nm

RMS (grain-wise): 97.1808 nm

Mean roughness (Sa): 82.6234 nm

Skew (Ssk): 0.104139

Kurtosis: -0.894700

6) Zn_0.4T_CO2_Quartz_XRD

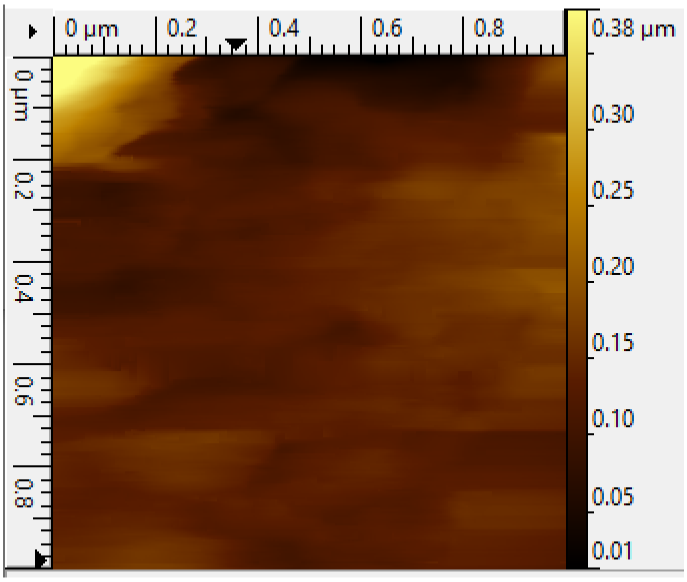

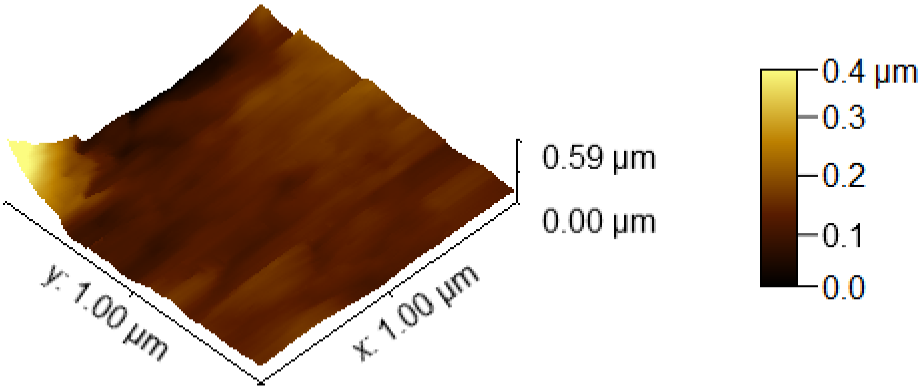

Statistical Quantities

Average value: 136.673 nm

RMS roughness (Sq): 40.4048 nm

RMS (grain-wise): 40.4048 nm

Mean roughness (Sa): 24.5386 nm

Skew (Ssk): 2.86225

Kurtosis: 19.3779

7) Zn_0.2T_N2_XRD

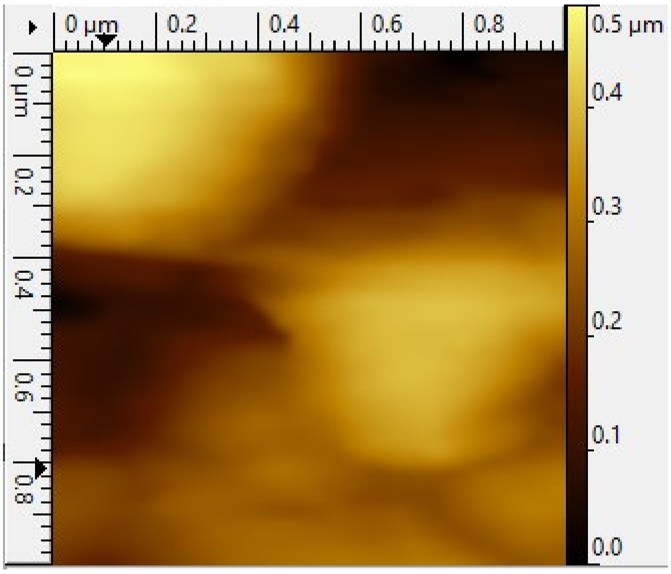

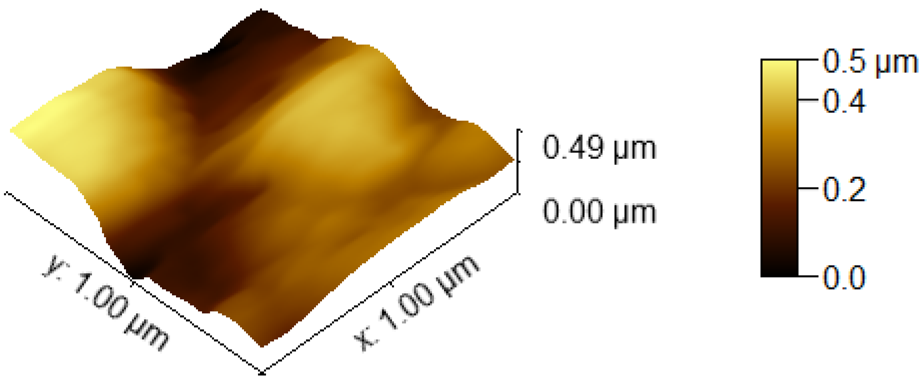

Statistical Quantities

Average value: 260.411 nm

RMS roughness (Sq): 107.752 nm

RMS (grain-wise): 107.752 nm

Mean roughness (Sa): 87.033 nm

Skew (Ssk): 81.0374×10^{-6}

Kurtosis: -0.701182

8) Zn_0.2T_CO2_Quartz

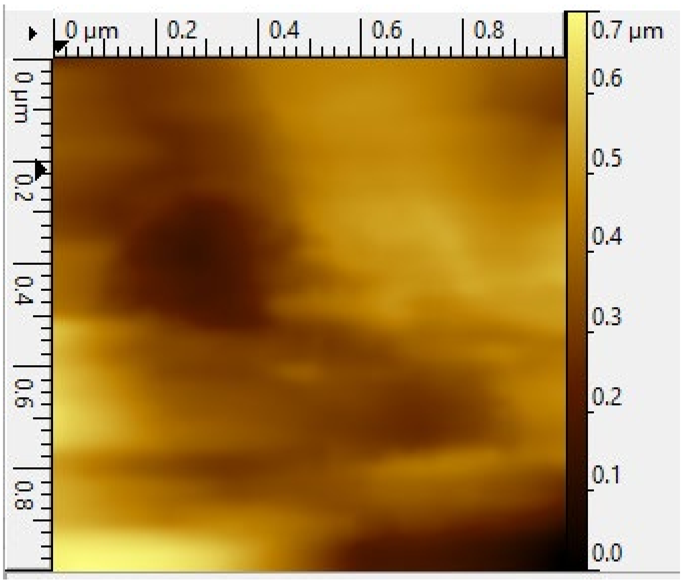

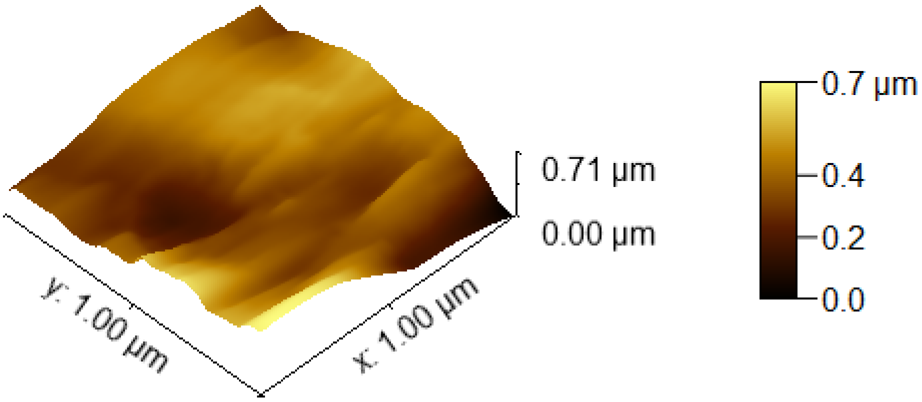

Statistical Quantities

Average value: 385.479 nm

RMS roughness (Sq): 110.594 nm

RMS (grain-wise): 110.594 nm

Mean roughness (Sa): 90.453 nm

Skew (Ssk): -6.03154×10^{-3}

Kurtosis: 0.0558848

9) Unknown_1

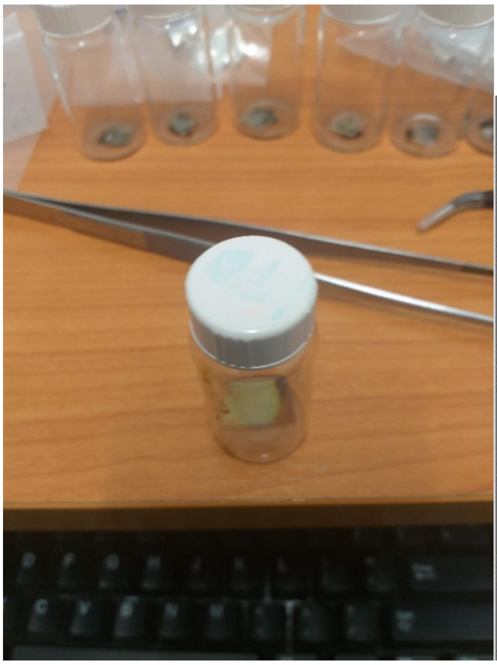

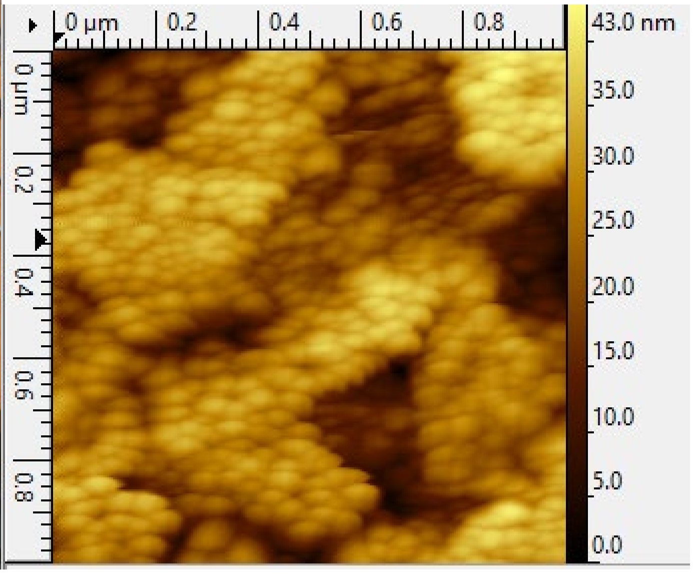

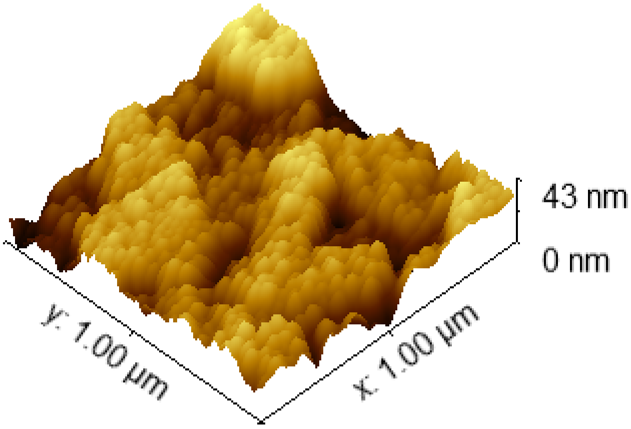

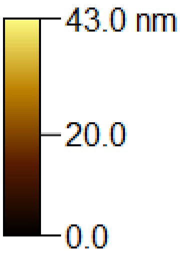

Statistical Quantities

Average value: 24.2175 nm

RMS roughness (Sq): 7.76204 nm

RMS (grain-wise): 7.76204 nm

Mean roughness (Sa): 6.37091 nm

Skew (Ssk): -0.374778

Kurtosis: -0.435304

10) Unknown_2

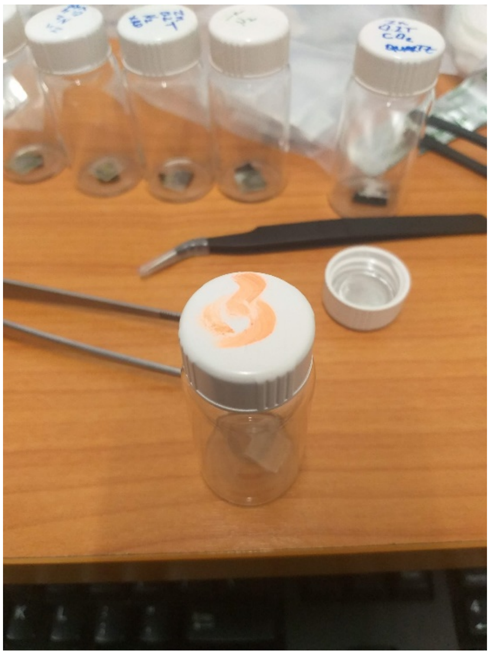

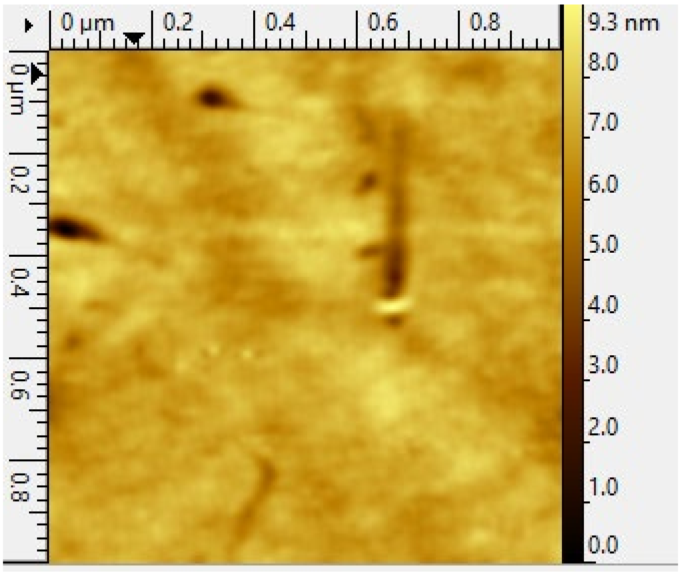

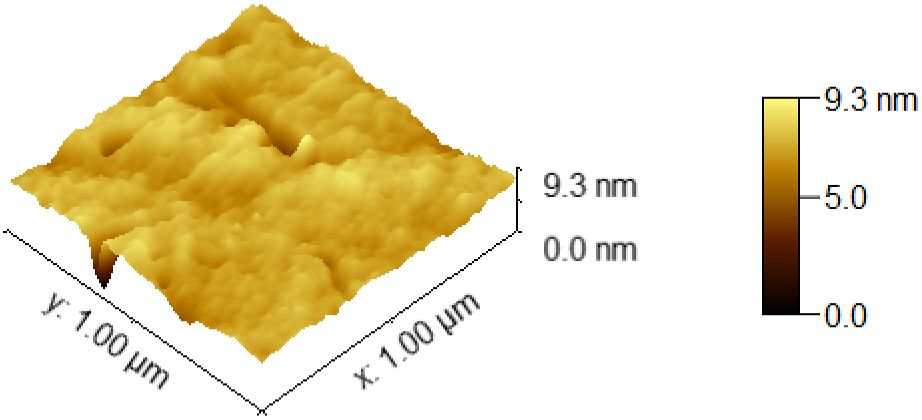

Statistical Quantities

Average value: 7.06336 nm

RMS roughness (Sq): 649.442 pm

RMS (grain-wise): 649.442 pm

Mean roughness (Sa): 456.815 pm

Skew (Ssk): -1.88099

Kurtosis: 11.5278
