# Supplementary figures and images for: Influence of the magnetic field on bandgap and chemical composition of zinc thin films prepared by sparking discharge process
Source: Sci Rep. 2020 Jan 29;10:1388. doi: 10.1038/s41598-020-58183-4 (PMC6989455; doi:10.1038/s41598-020-58183-4)

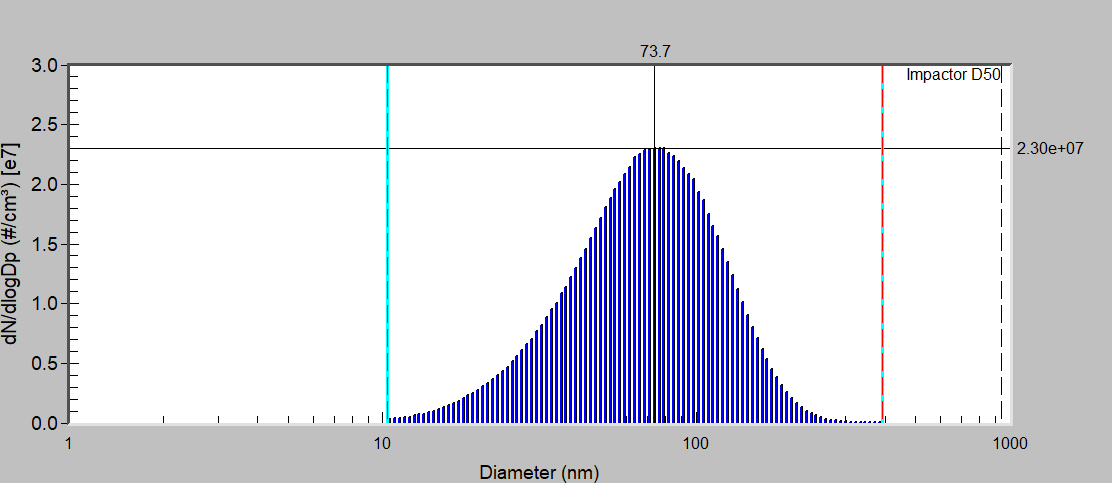

Supplement: Supplementary file 2 — Related Manuscript File. [file 41598_2020_58183_MOESM2_ESM.zip › thumbnail_pastedImage (1).png]

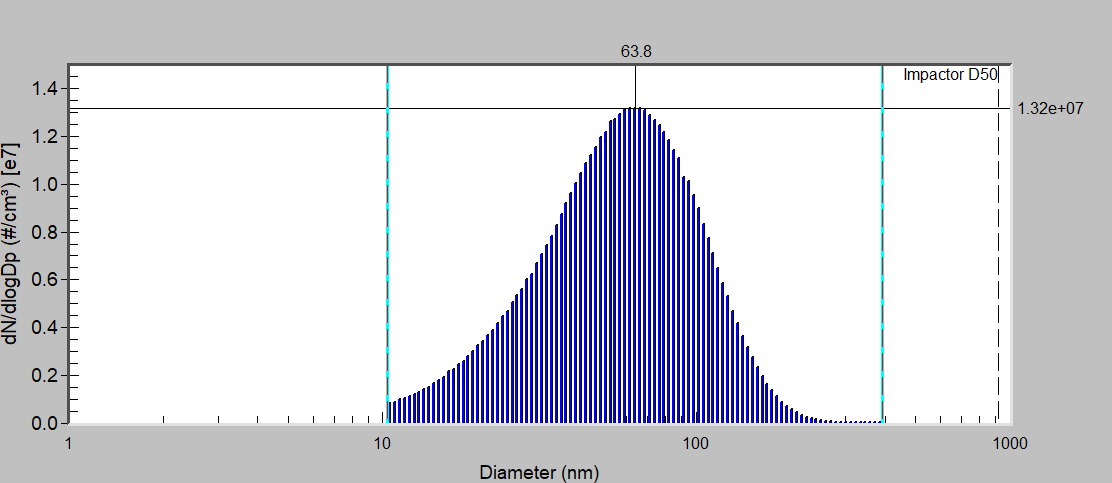

Supplement: Supplementary file 2 — Related Manuscript File. [file 41598_2020_58183_MOESM2_ESM.zip › thumbnail_pastedImage (10).png]

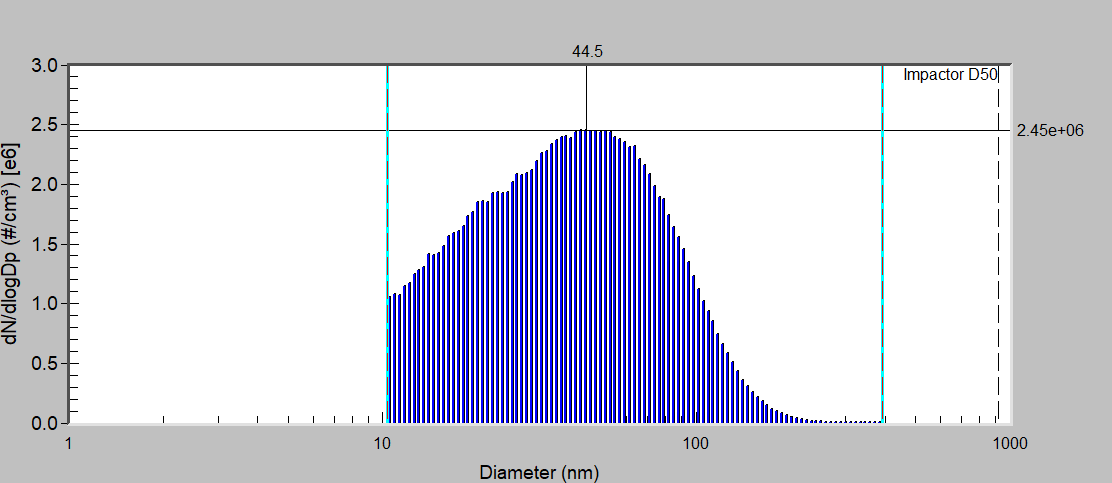

Supplement: Supplementary file 2 — Related Manuscript File. [file 41598_2020_58183_MOESM2_ESM.zip › thumbnail_pastedImage (11).png]

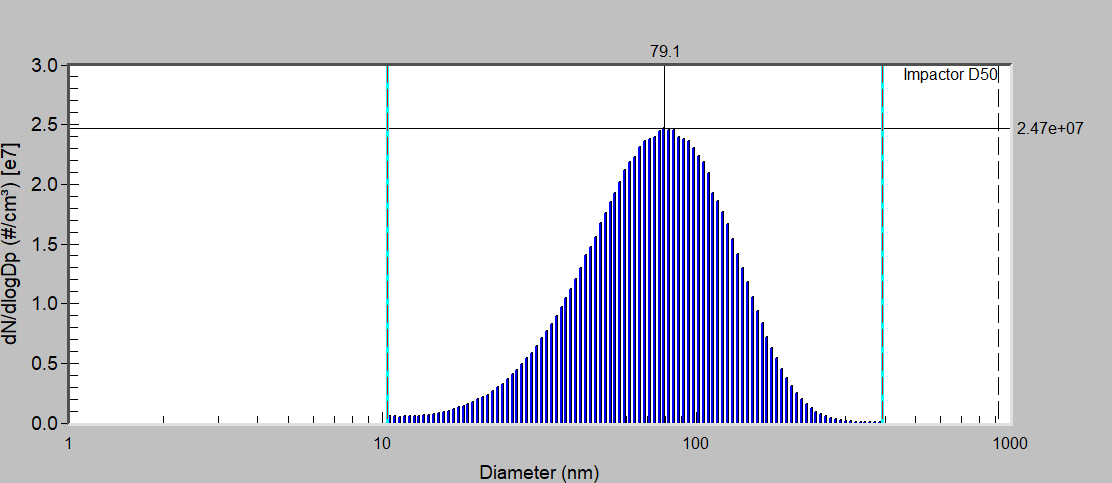

Supplement: Supplementary file 2 — Related Manuscript File. [file 41598_2020_58183_MOESM2_ESM.zip › thumbnail_pastedImage (12).png]

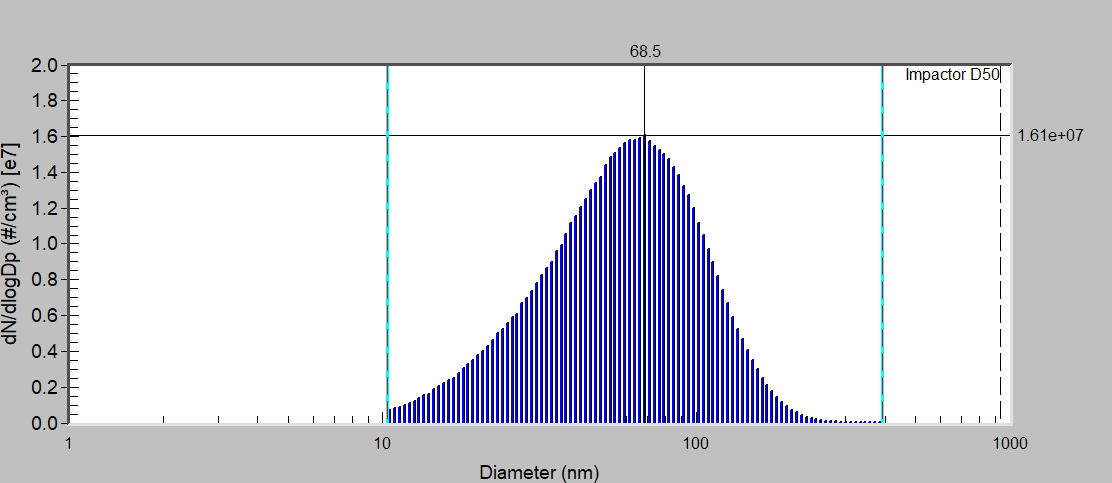

Supplement: Supplementary file 2 — Related Manuscript File. [file 41598_2020_58183_MOESM2_ESM.zip › thumbnail_pastedImage (2).png]

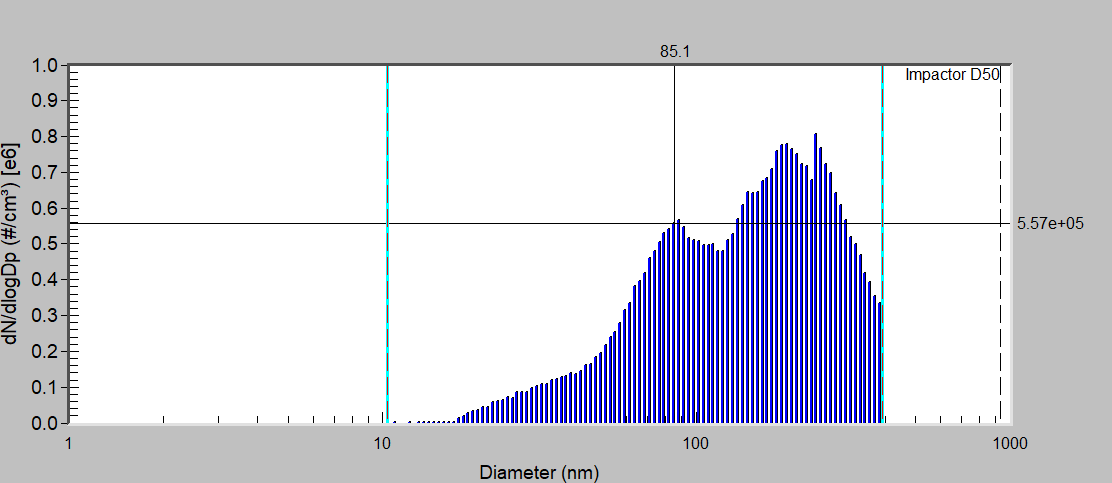

Supplement: Supplementary file 2 — Related Manuscript File. [file 41598_2020_58183_MOESM2_ESM.zip › thumbnail_pastedImage (3).png]

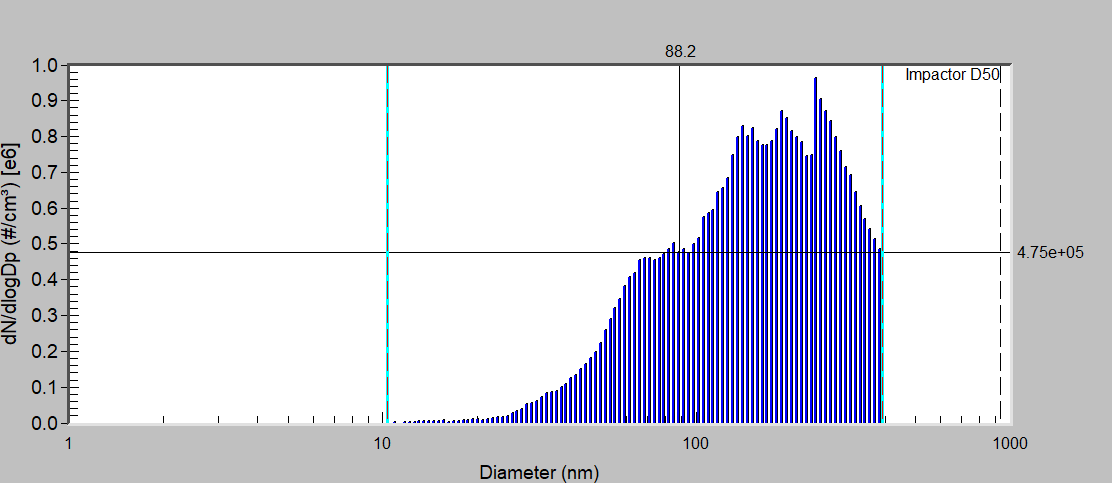

Supplement: Supplementary file 2 — Related Manuscript File. [file 41598_2020_58183_MOESM2_ESM.zip › thumbnail_pastedImage (4).png]

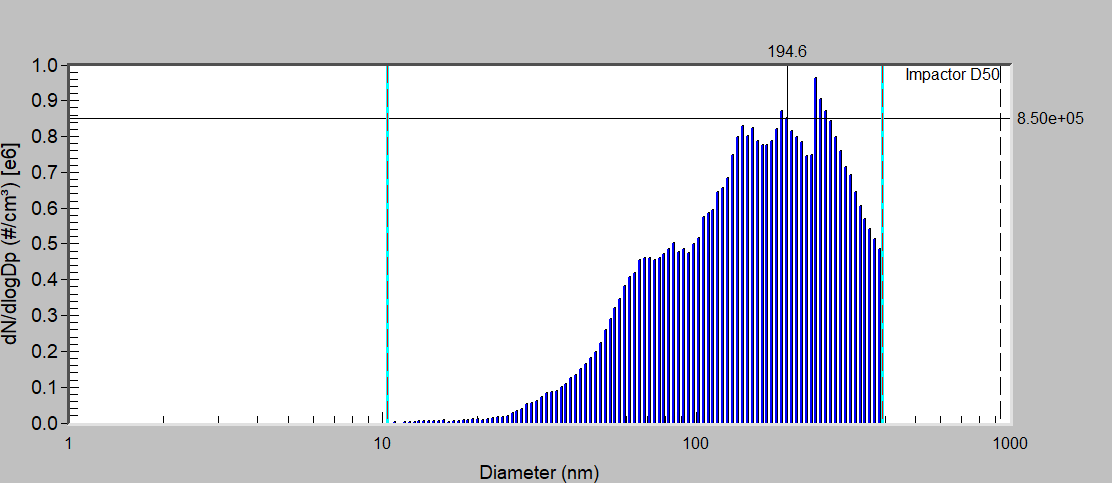

Supplement: Supplementary file 2 — Related Manuscript File. [file 41598_2020_58183_MOESM2_ESM.zip › thumbnail_pastedImage (5).png]

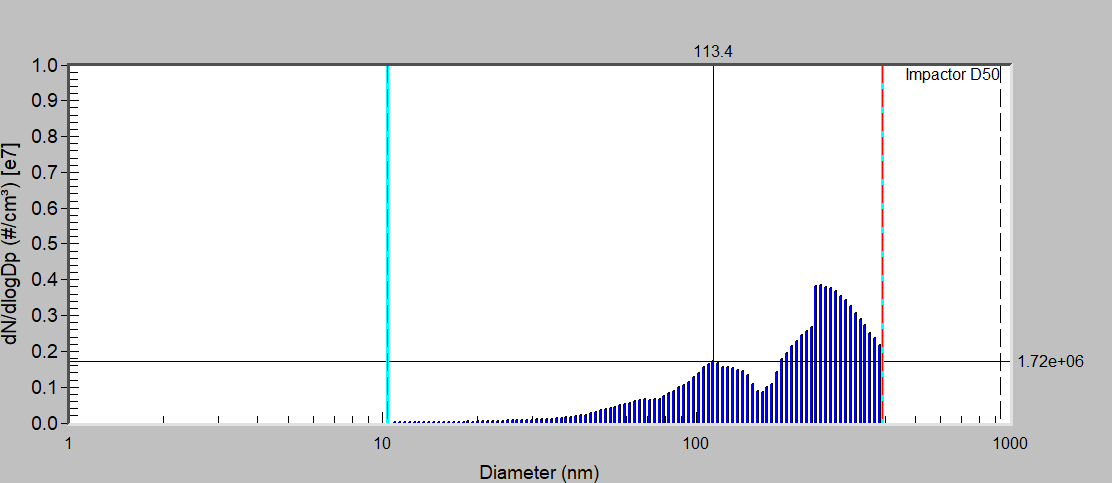

Supplement: Supplementary file 2 — Related Manuscript File. [file 41598_2020_58183_MOESM2_ESM.zip › thumbnail_pastedImage (6).png]

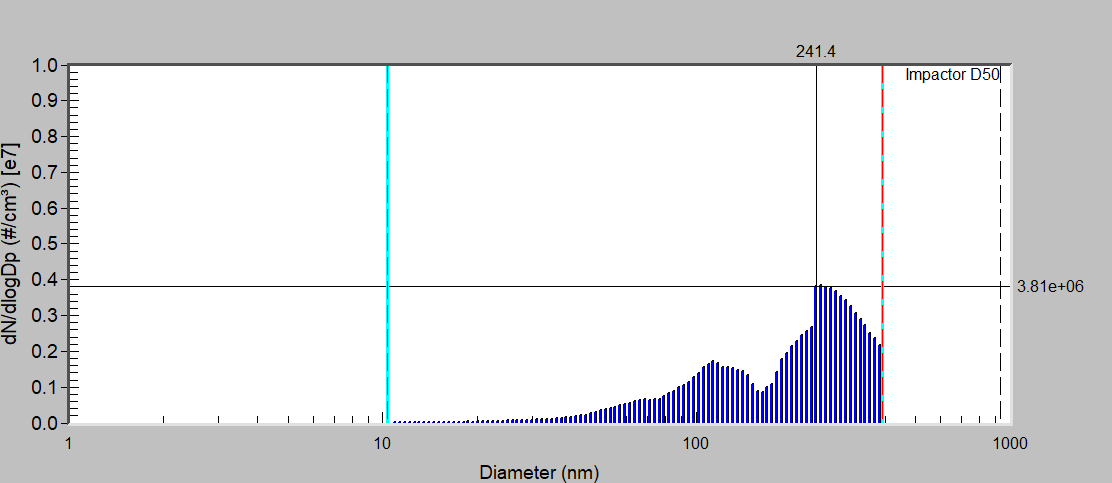

Supplement: Supplementary file 2 — Related Manuscript File. [file 41598_2020_58183_MOESM2_ESM.zip › thumbnail_pastedImage (7).png]

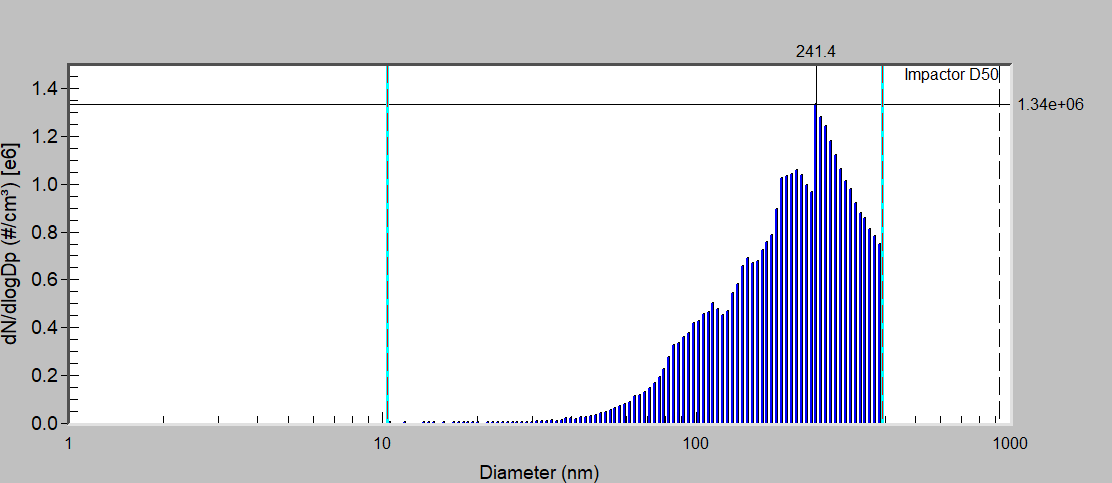

Supplement: Supplementary file 2 — Related Manuscript File. [file 41598_2020_58183_MOESM2_ESM.zip › thumbnail_pastedImage (8).png]

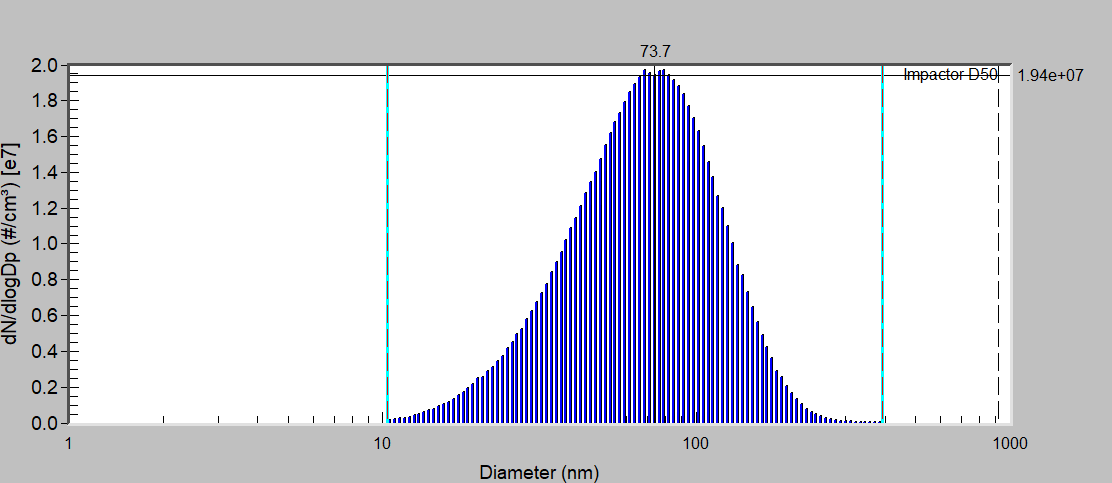

Supplement: Supplementary file 2 — Related Manuscript File. [file 41598_2020_58183_MOESM2_ESM.zip › thumbnail_pastedImage (9).png]

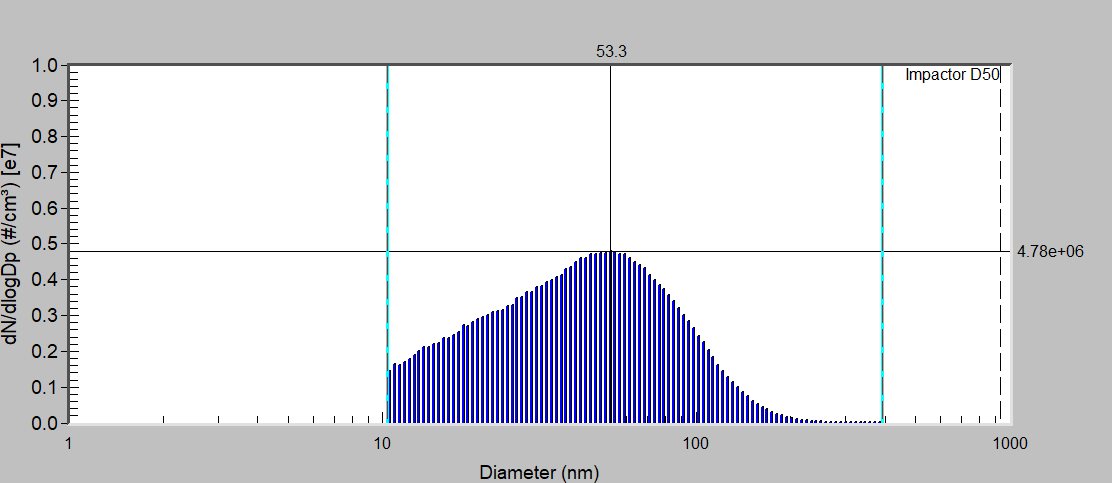

Supplement: Supplementary file 2 — Related Manuscript File. [file 41598_2020_58183_MOESM2_ESM.zip › thumbnail_pastedImage.png]

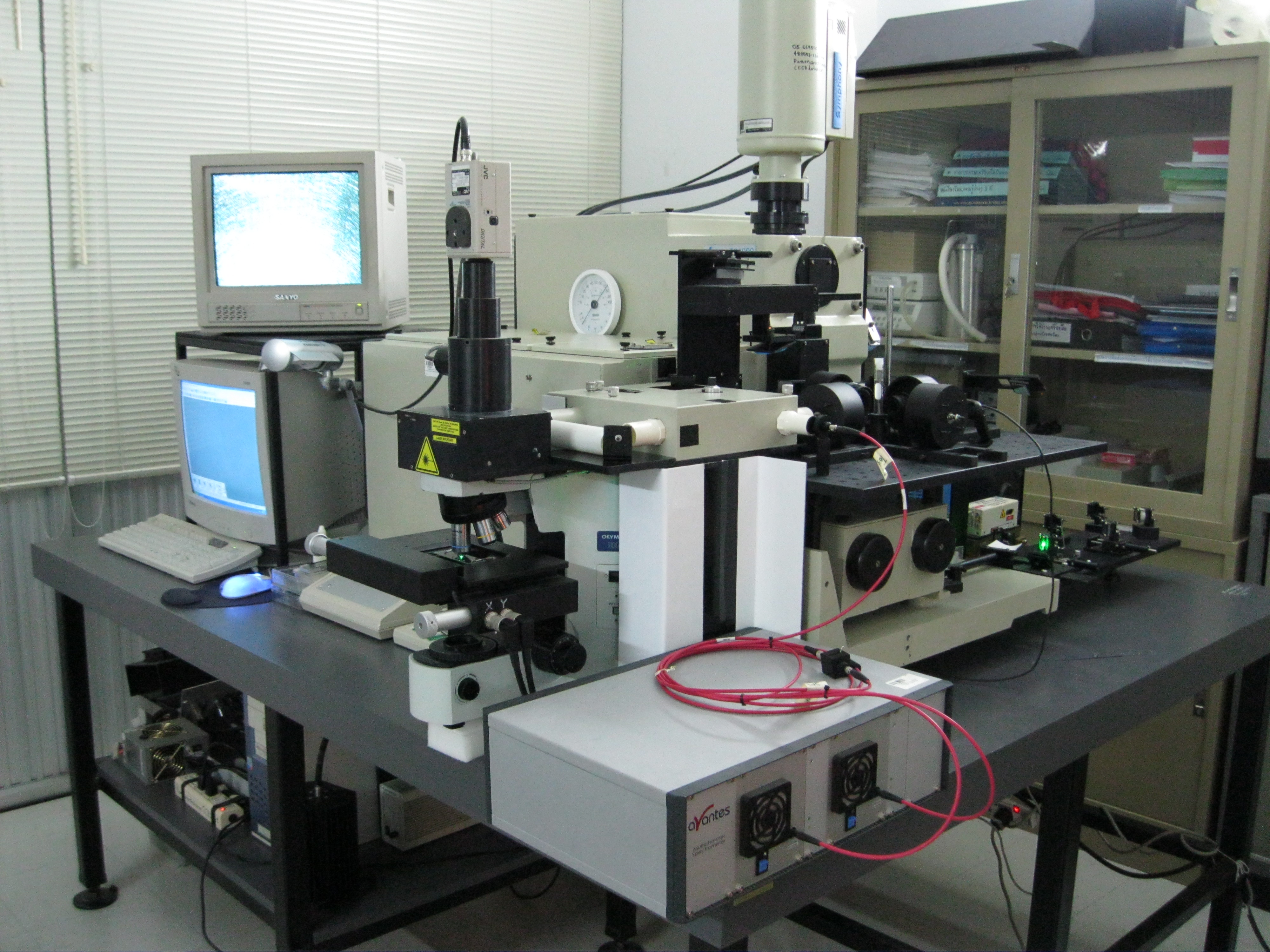

Supplement: Supplementary file 4 — Related Manuscript File. [file 41598_2020_58183_MOESM4_ESM.zip › RAMAN SPECTROMETER.JPG]
