# Supplementary material for: Influence of the magnetic field on bandgap and chemical composition of zinc thin films prepared by sparking discharge process
Source: Sci Rep. 2020 Jan 29;10:1388. doi: 10.1038/s41598-020-58183-4 (PMC6989455; doi:10.1038/s41598-020-58183-4)
Supplement: Supplementary file 5 — Related Manuscript File. [file 41598_2020_58183_MOESM5_ESM.zip › XPS_7-3-2019_Stefan/XPS_7-3-2019/1_ZnN2-0.2T_ITO/component.pdf]

Zn 2p:3(7-3-2019)

XPS Spectrum Lens Mode:Hybrid Resolution:Pass energy 20 Iris(Aper):slot(Slot)

Acqn. Time(s): 180 Sweeps: 3 Anode:Mono(Al (Mono))(150 W) Step(meV): 100.0

Dwell Time(ms): 140 Charge Neutraliser :On Acquired On :19/03/07 09:26:28

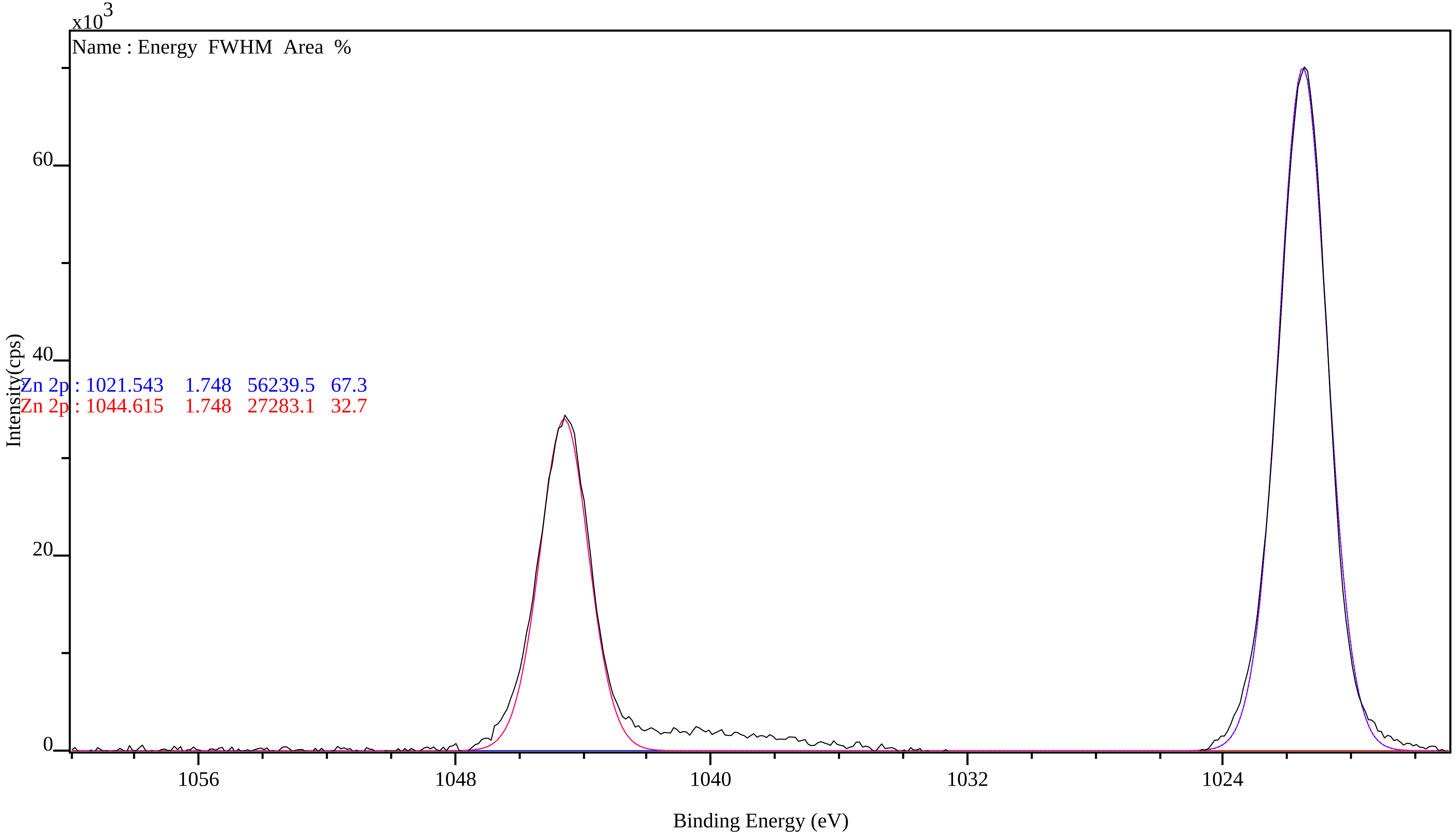

Ni 2p:4(7-3-2019)

XPS Spectrum Lens Mode:Hybrid Resolution:Pass energy 20 Iris(Aper):slot(Slot)

Acqn. Time(s): 180 Sweeps: 3 Anode:Mono(Al (Mono))(150 W) Step(meV): 100.0

Dwell Time(ms): 154 Charge Neutraliser :On Acquired On :19/03/07 09:26:28

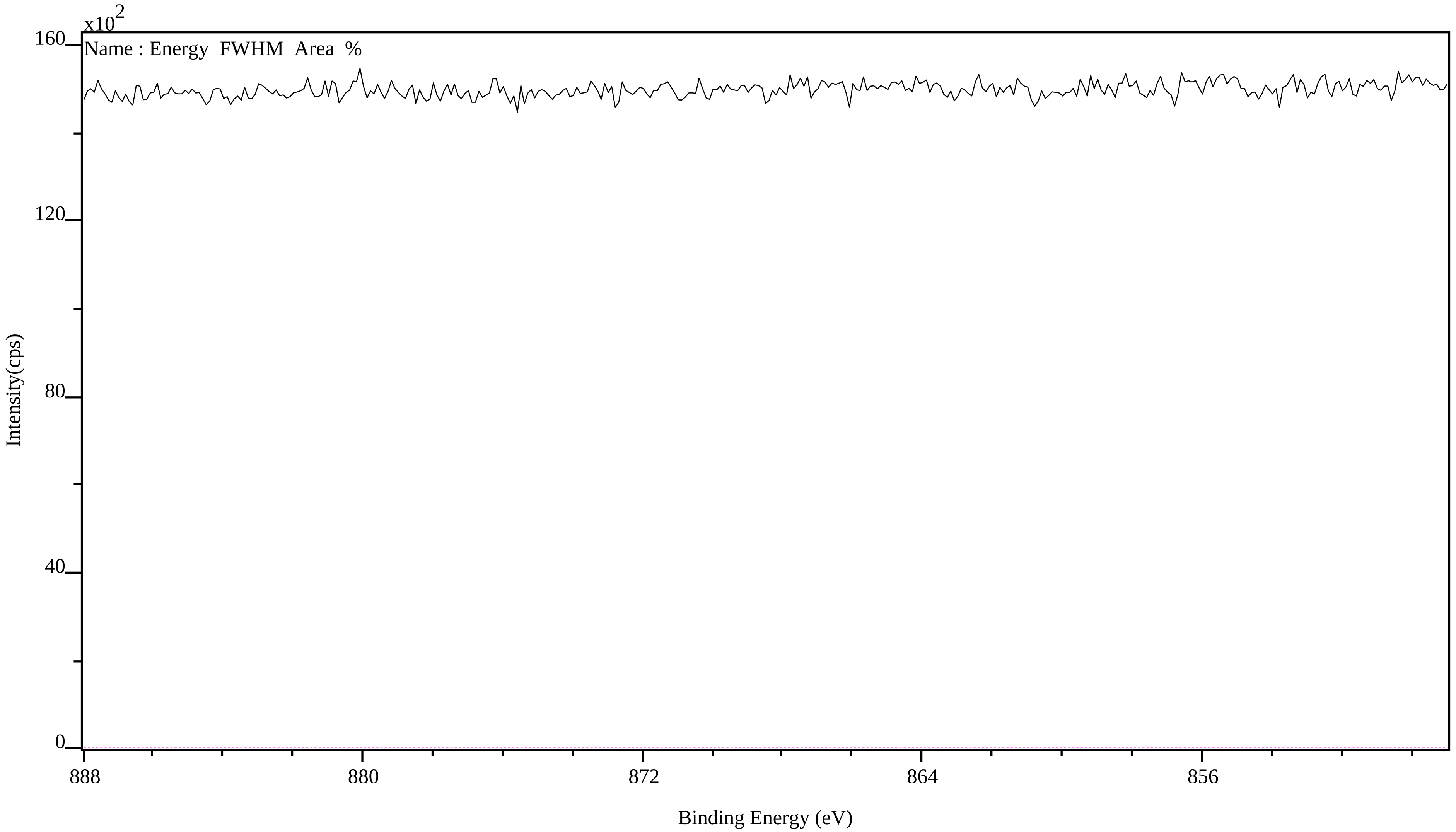

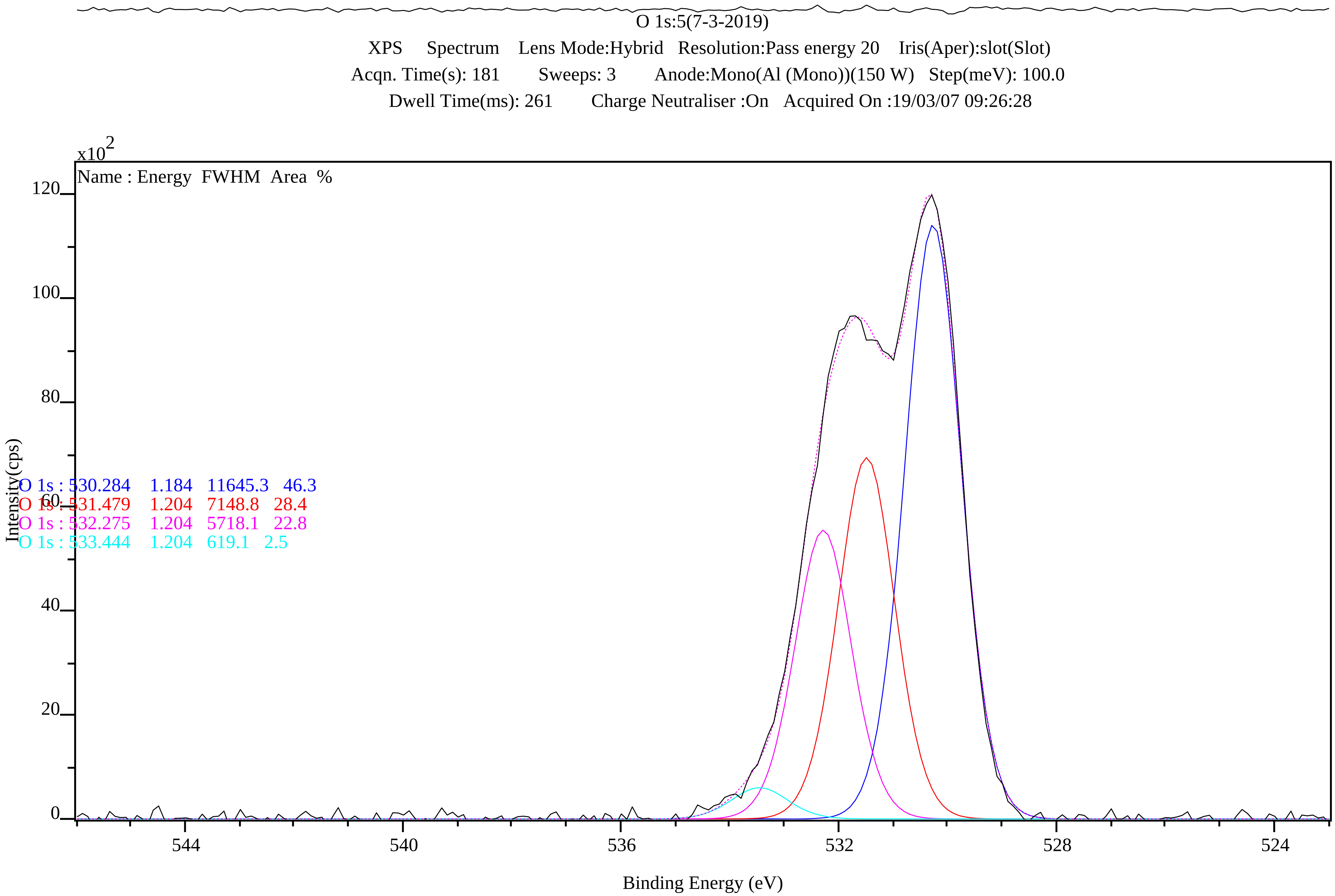

O 1s:5(7-3-2019)
XPS Spectrum Lens Mode:Hybrid Resolution:Pass energy 20 Iris(Aper):slot(Slot)
Acqn. Time(s): 181 Sweeps: 3 Anode:Mono(Al (Mono))(150 W) Step(meV): 100.0
Dwell Time(ms): 261 Charge Neutraliser :On Acquired On :19/03/07 09:26:28
x10^2
Name : Energy FWHM Area %
O 1s : 530.284 1.184 11645.3 46.3
O 1s : 531.479 1.204 7148.8 28.4
O 1s : 532.275 1.204 5718.1 22.8
O 1s : 533.444 1.204 619.1 2.5
Intensity(cps)
120
100
80
60
40
20
0
544
540
536
532
528
524
Binding Energy (eV)

N 1s:6(7-3-2019)

XPS Spectrum Lens Mode:Hybrid Resolution:Pass energy 20 Iris(Aper):slot(Slot)

Acqn. Time(s): 181 Sweeps: 3 Anode:Mono(Al (Mono))(150 W) Step(meV): 100.0

Dwell Time(ms): 286 Charge Neutraliser :On Acquired On :19/03/07 09:26:28

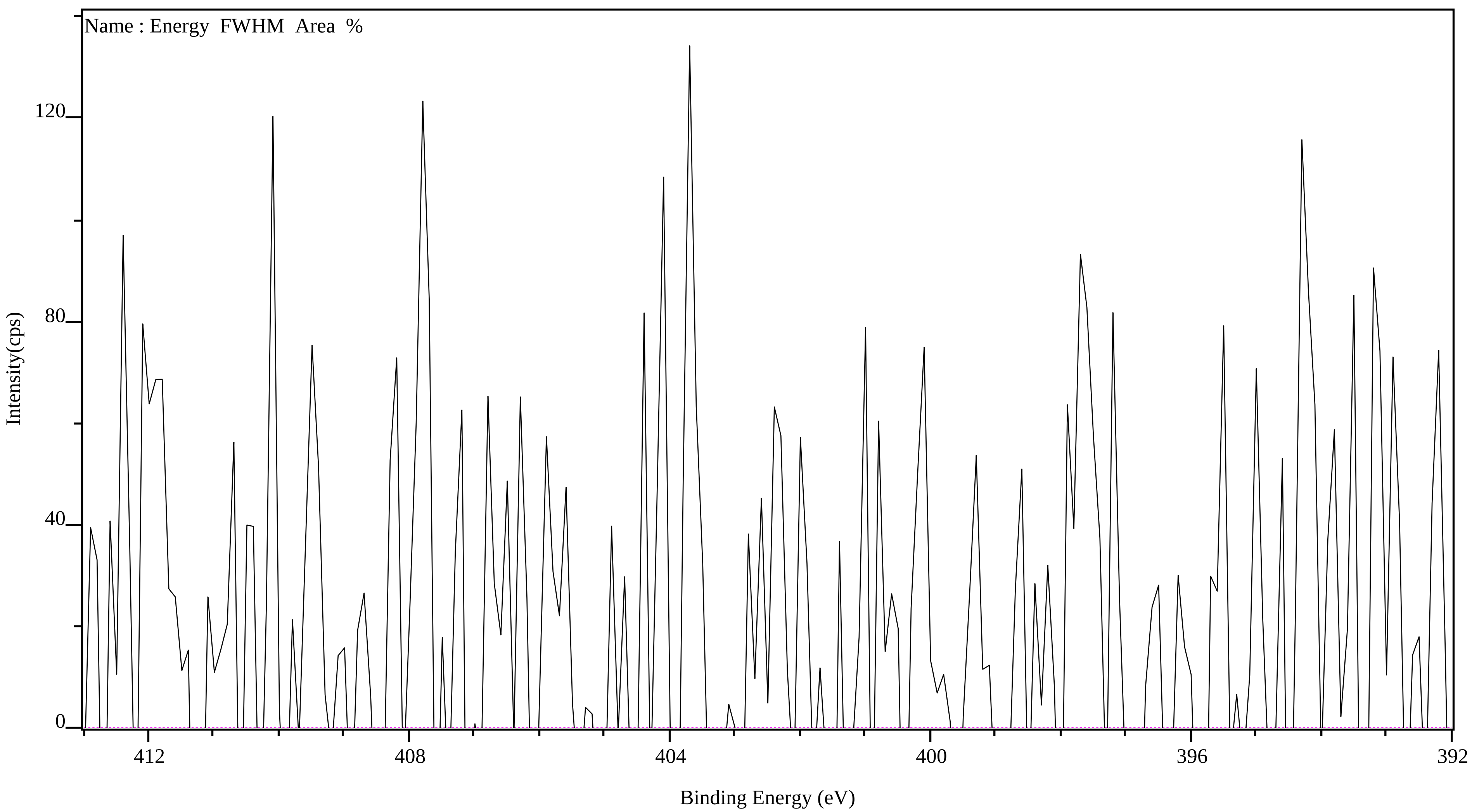

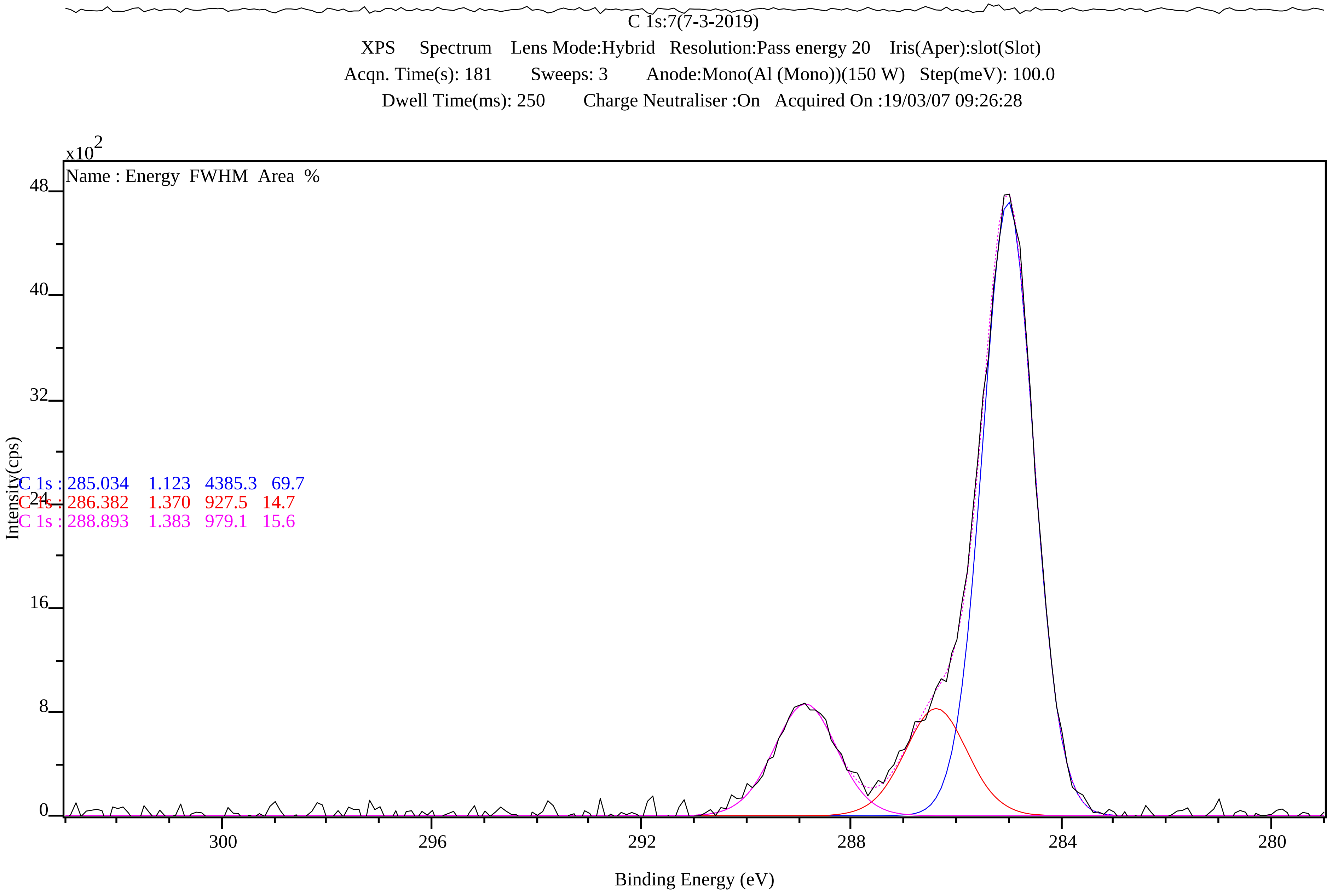
C 1s:7(7-3-2019)
XPS Spectrum Lens Mode:Hybrid Resolution:Pass energy 20 Iris(Aper):slot(Slot)
Acqn. Time(s): 181 Sweeps: 3 Anode:Mono(Al (Mono))(150 W) Step(meV): 100.0
Dwell Time(ms): 250 Charge Neutraliser :On Acquired On :19/03/07 09:26:28
x10^2
Name : Energy FWHM Area %
C 1s : 285.034 1.123 4385.3 69.7
C 1s : 286.382 1.370 927.5 14.7
C 1s : 288.893 1.383 979.1 15.6
Intensity(cps)
48
40
32
24
16
8
0
300
296
292
288
284
280
Binding Energy (eV)
