# Supplementary material for: Influence of the magnetic field on bandgap and chemical composition of zinc thin films prepared by sparking discharge process
Source: Sci Rep. 2020 Jan 29;10:1388. doi: 10.1038/s41598-020-58183-4 (PMC6989455; doi:10.1038/s41598-020-58183-4)
Supplement: Supplementary file 5 — Related Manuscript File. [file 41598_2020_58183_MOESM5_ESM.zip › XPS_7-3-2019_Stefan/XPS_7-3-2019/1_ZnN2-0.2T_ITO/report.pdf]

Quantification Report

/C=/data/7-3-2019/7-3-2019.dset Thu Mar 7 15:57:44 2019

State : Angle Name : Position 1

Peak	Type	Position BE (eV)	FWHM (eV)	Raw Area (cps eV)	RSF	Atomic Mass	Atomic Conc %	Mass Conc %
Zn 2p	Reg	1021.000	2.389	1424839.9	5.589	65.387	28.36	64.70
O 1s	Reg	531.000	3.284	242580.0	0.780	15.999	37.94	21.18
C 1s	Reg	285.000	2.169	71530.0	0.278	12.011	33.70	14.12
