# Supplementary material for: Influence of the magnetic field on bandgap and chemical composition of zinc thin films prepared by sparking discharge process
Source: Sci Rep. 2020 Jan 29;10:1388. doi: 10.1038/s41598-020-58183-4 (PMC6989455; doi:10.1038/s41598-020-58183-4)
Supplement: Supplementary file 5 — Related Manuscript File. [file 41598_2020_58183_MOESM5_ESM.zip › XPS_7-3-2019_Stefan/XPS_7-3-2019/2_ZnCO2-0.2T_ITO/component.pdf]

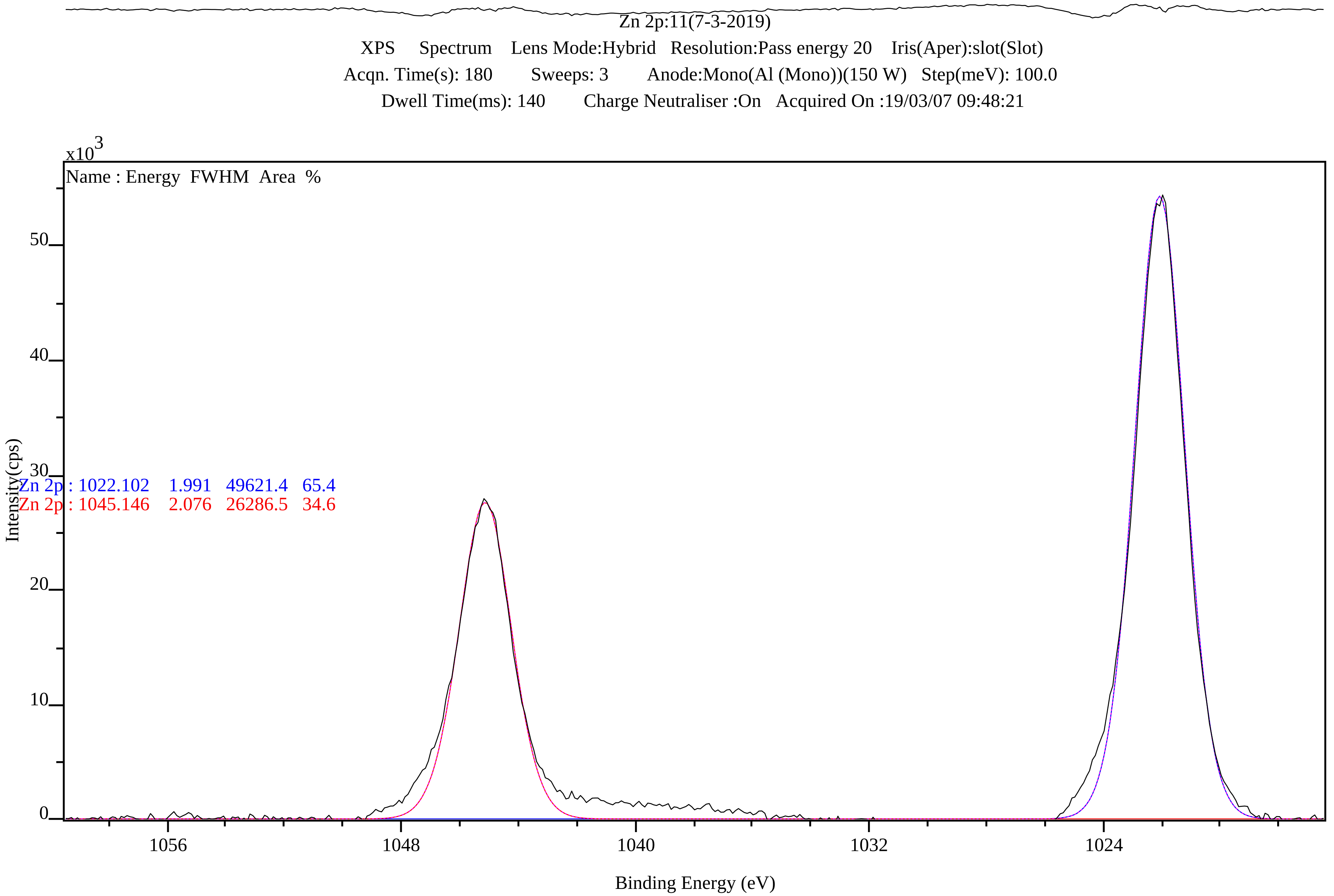
Zn 2p:11(7-3-2019)
XPS Spectrum Lens Mode:Hybrid Resolution:Pass energy 20 Iris(Aper):slot(Slot)
Acqn. Time(s): 180 Sweeps: 3 Anode:Mono(Al (Mono))(150 W) Step(meV): 100.0
Dwell Time(ms): 140 Charge Neutraliser :On Acquired On :19/03/07 09:48:21
x10^3
Name : Energy FWHM Area %
Zn 2p : 1022.102 1.991 49621.4 65.4
Zn 2p : 1045.146 2.076 26286.5 34.6
Intensity(cps)
50
40
30
20
10
0
1056
1048
1040
1032
1024
Binding Energy (eV)

Ni 2p:12(7-3-2019)

XPS Spectrum Lens Mode:Hybrid Resolution:Pass energy 20 Iris(Aper):slot(Slot)

Acqn. Time(s): 180 Sweeps: 3 Anode:Mono(Al (Mono))(150 W) Step(meV): 100.0

Dwell Time(ms): 154 Charge Neutraliser :On Acquired On :19/03/07 09:48:21

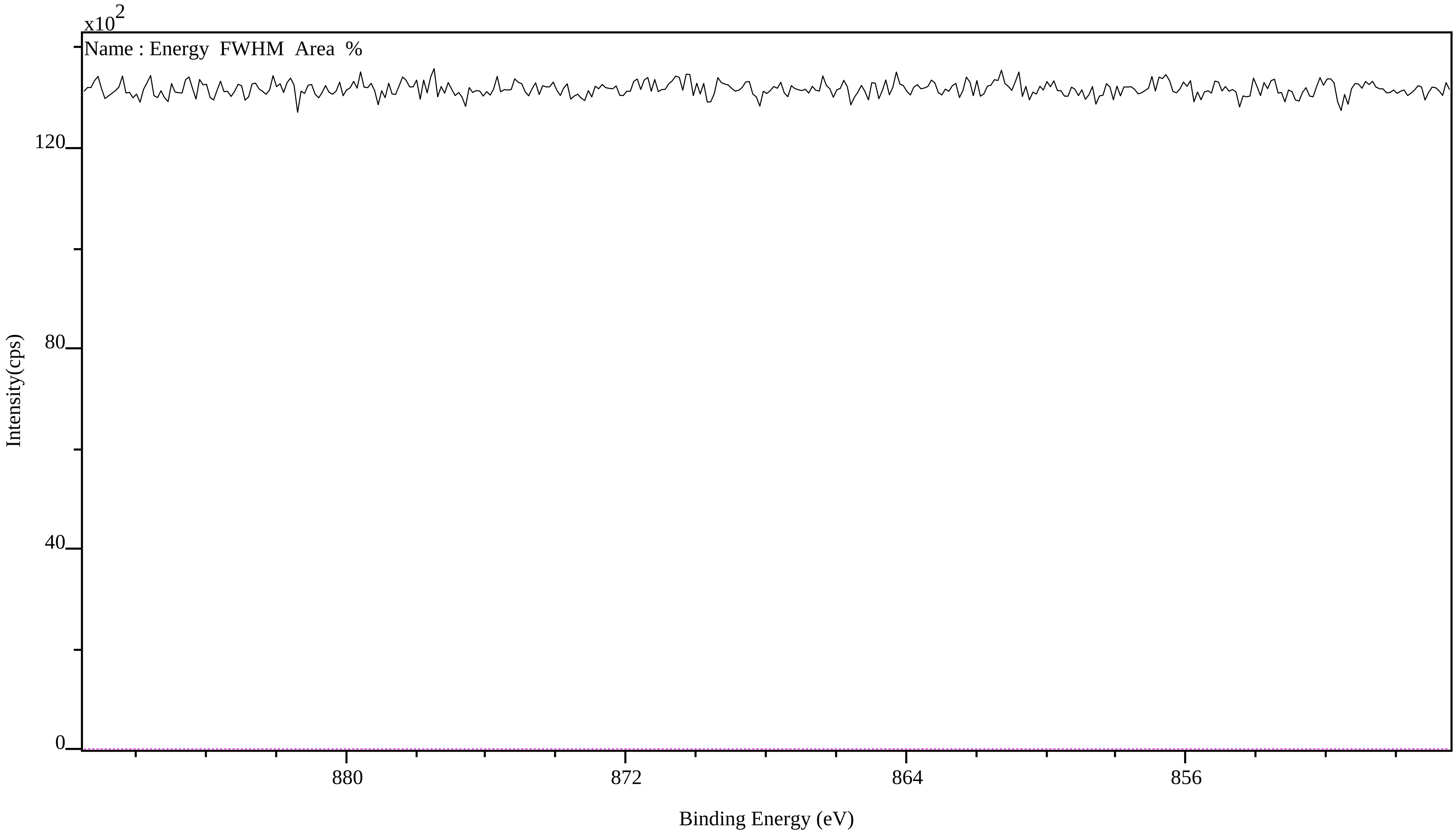

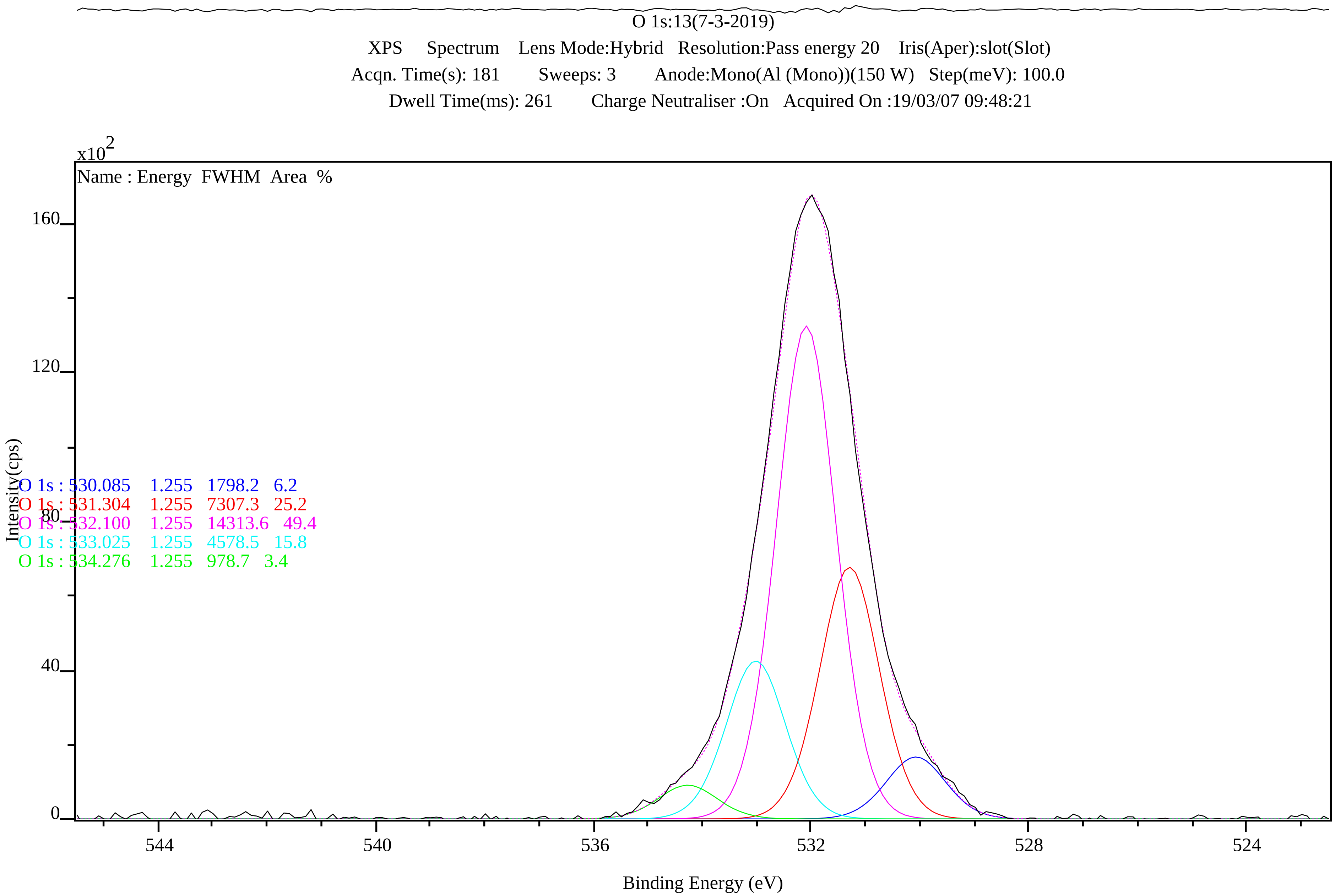

O 1s:13(7-3-2019)
XPS Spectrum Lens Mode:Hybrid Resolution:Pass energy 20 Iris(Aper):slot(Slot)
Acqn. Time(s): 181 Sweeps: 3 Anode:Mono(Al (Mono))(150 W) Step(meV): 100.0
Dwell Time(ms): 261 Charge Neutraliser :On Acquired On :19/03/07 09:48:21
x10^2
Name : Energy FWHM Area %
O 1s : 530.085 1.255 1798.2 6.2
O 1s : 531.304 1.255 7307.3 25.2
O 1s : 532.100 1.255 14313.6 49.4
O 1s : 533.025 1.255 4578.5 15.8
O 1s : 534.276 1.255 978.7 3.4
Intensity(cps)
160
120
80
40
0
544
540
536
532
528
524
Binding Energy (eV)

N 1s:14(7-3-2019)

XPS Spectrum Lens Mode:Hybrid Resolution:Pass energy 20 Iris(Aper):slot(Slot)

Acqn. Time(s): 181 Sweeps: 3 Anode:Mono(Al (Mono))(150 W) Step(meV): 100.0

Dwell Time(ms): 286 Charge Neutraliser :On Acquired On :19/03/07 09:48:21

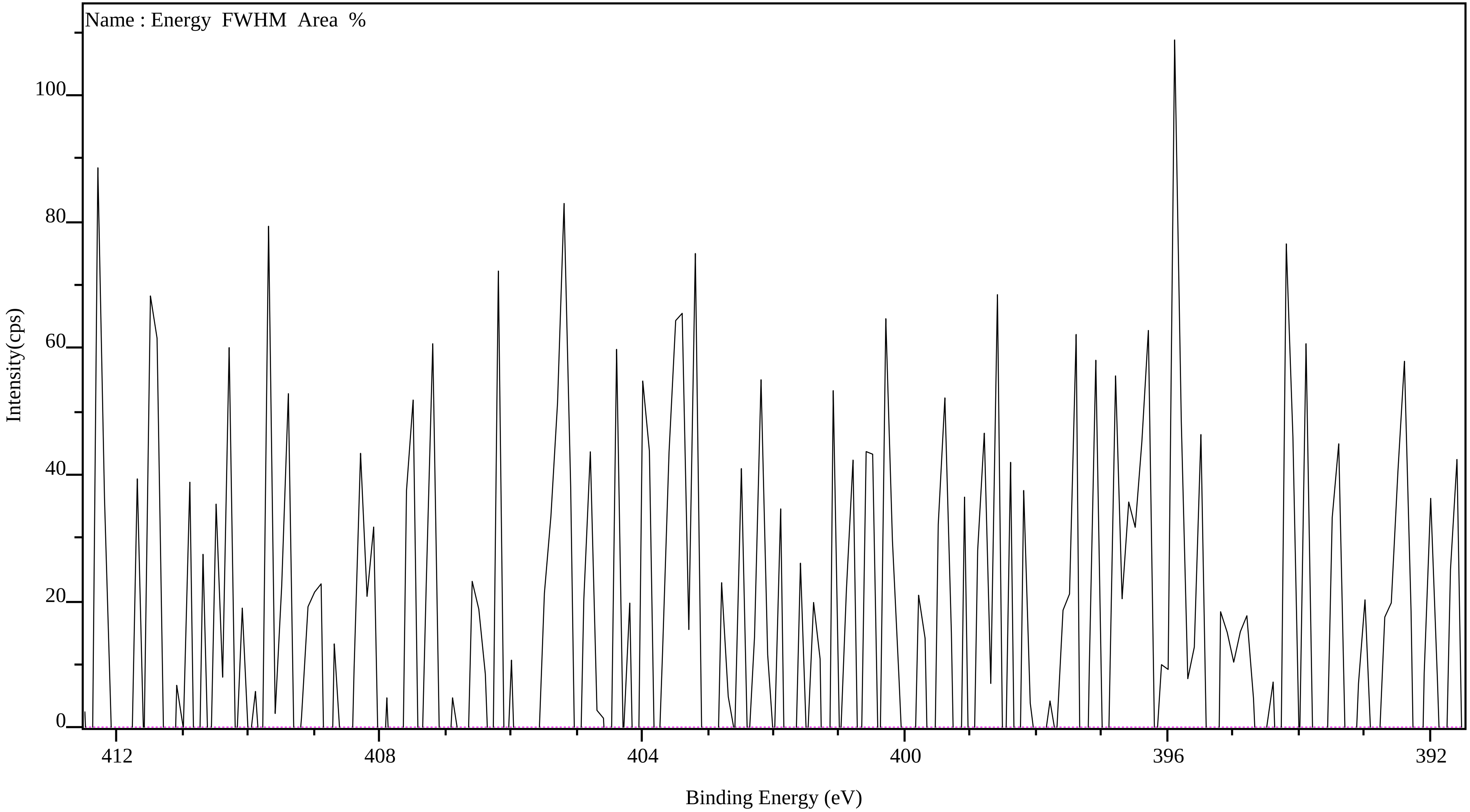

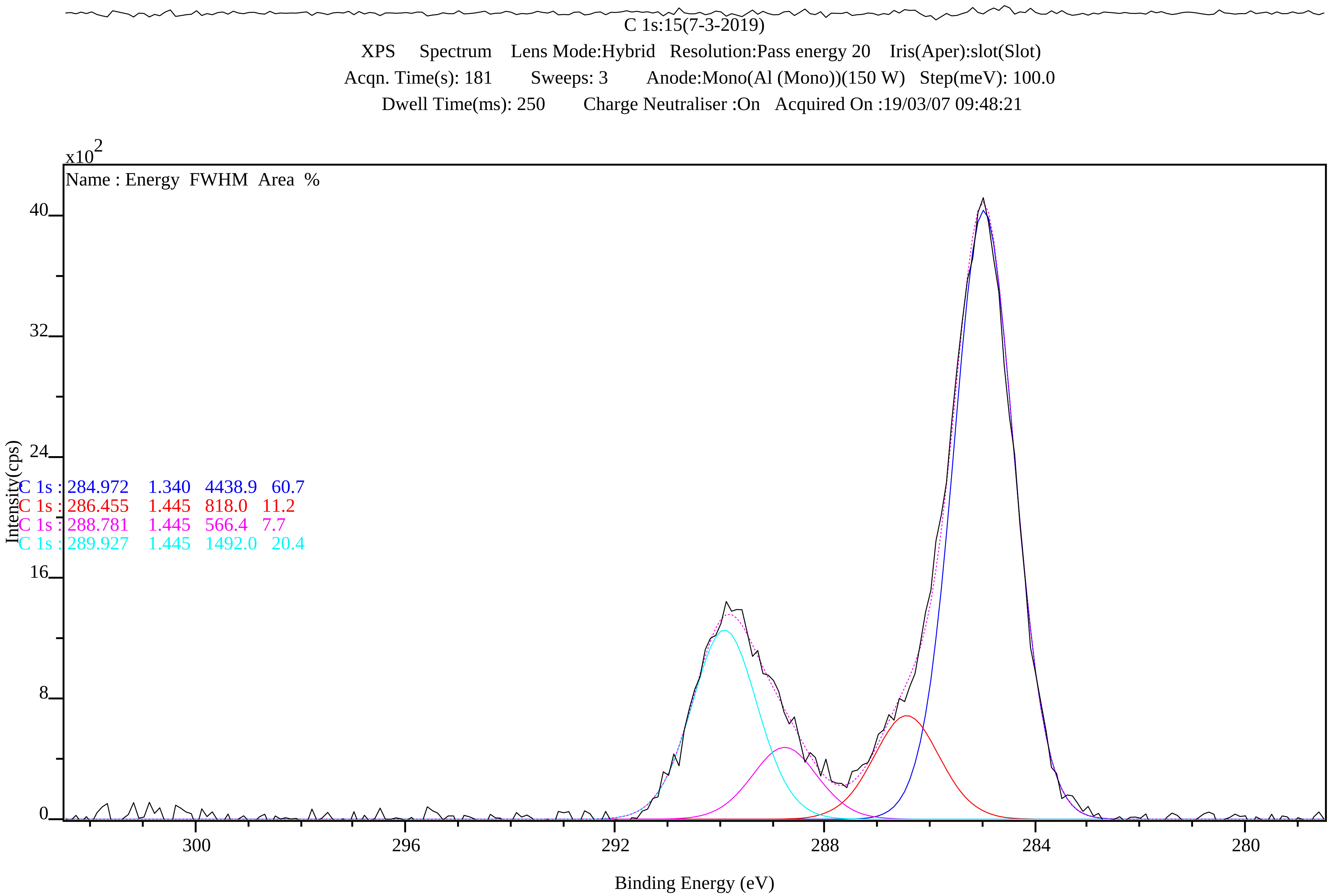
C 1s:15(7-3-2019)
XPS Spectrum Lens Mode:Hybrid Resolution:Pass energy 20 Iris(Aper):slot(Slot)
Acqn. Time(s): 181 Sweeps: 3 Anode:Mono(Al (Mono))(150 W) Step(meV): 100.0
Dwell Time(ms): 250 Charge Neutraliser :On Acquired On :19/03/07 09:48:21
x10^2
Name : Energy FWHM Area %
C 1s : 284.972 1.340 4438.9 60.7
C 1s : 286.455 1.445 818.0 11.2
C 1s : 288.781 1.445 566.4 7.7
C 1s : 289.927 1.445 1492.0 20.4
Intensity(cps)
40
32
24
16
8
0
300
296
292
288
284
280
Binding Energy (eV)
