# Supplementary material for: Influence of the magnetic field on bandgap and chemical composition of zinc thin films prepared by sparking discharge process
Source: Sci Rep. 2020 Jan 29;10:1388. doi: 10.1038/s41598-020-58183-4 (PMC6989455; doi:10.1038/s41598-020-58183-4)
Supplement: Supplementary file 5 — Related Manuscript File. [file 41598_2020_58183_MOESM5_ESM.zip › XPS_7-3-2019_Stefan/XPS_7-3-2019/3_ZnCO2-0.4T_ITO/component.pdf]

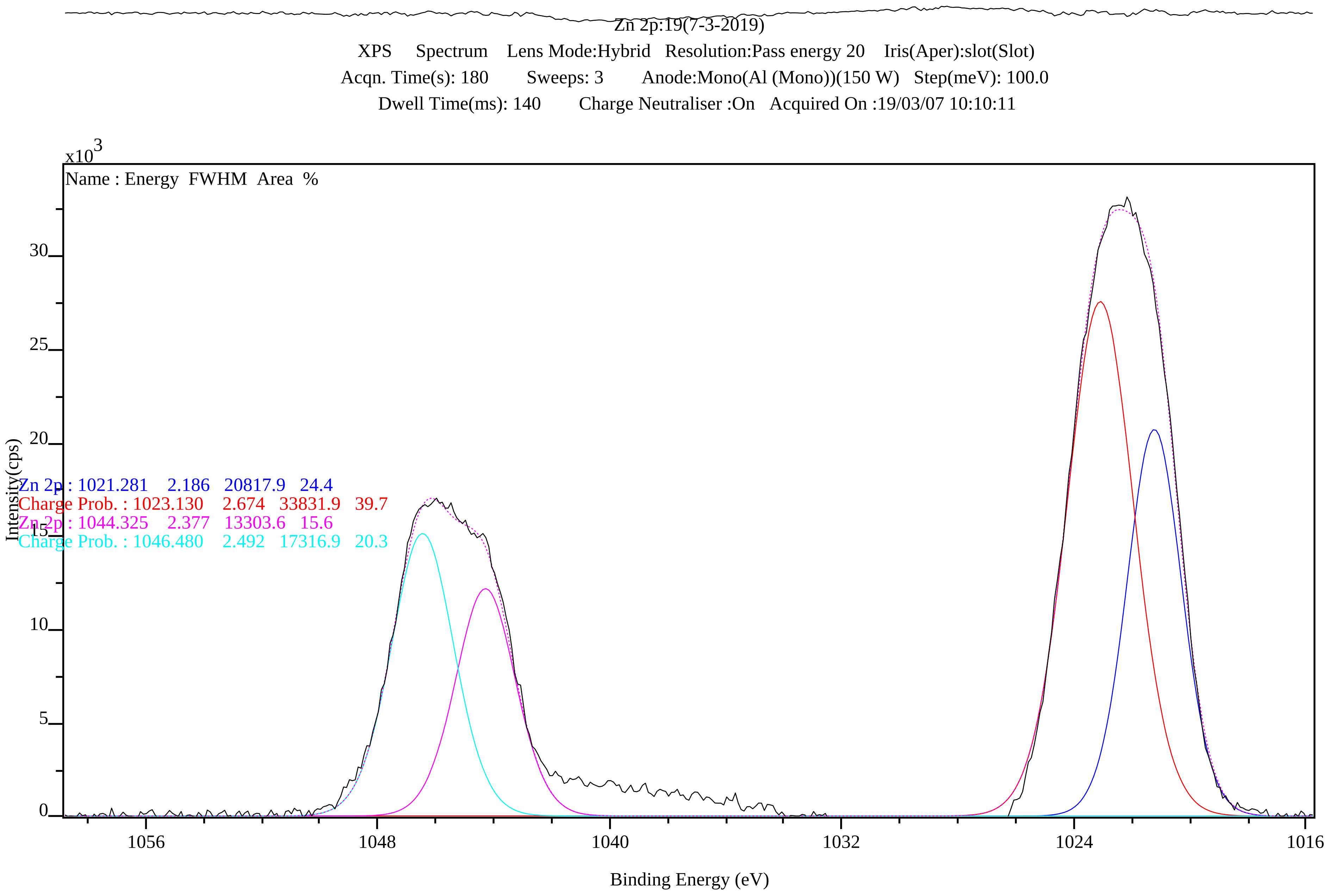

Zn 2p:19(7-3-2019)
XPS Spectrum Lens Mode:Hybrid Resolution:Pass energy 20 Iris(Aper):slot(Slot)
Acqn. Time(s): 180 Sweeps: 3 Anode:Mono(Al (Mono))(150 W) Step(meV): 100.0
Dwell Time(ms): 140 Charge Neutraliser :On Acquired On :19/03/07 10:10:11
x10^3
Name : Energy FWHM Area %
Zn 2p : 1021.281 2.186 20817.9 24.4
Charge Prob. : 1023.130 2.674 33831.9 39.7
Zn 2p : 1044.325 2.377 13303.6 15.6
Charge Prob. : 1046.480 2.492 17316.9 20.3
Intensity(cps)
30
25
20
15
10
5
0
1056
1048
1040
1032
1024
1016
Binding Energy (eV)

Ni 2p:20(7-3-2019)

XPS Spectrum Lens Mode:Hybrid Resolution:Pass energy 20 Iris(Aper):slot(Slot)

Acqn. Time(s): 180 Sweeps: 3 Anode:Mono(Al (Mono))(150 W) Step(meV): 100.0

Dwell Time(ms): 154 Charge Neutraliser :On Acquired On :19/03/07 10:10:11

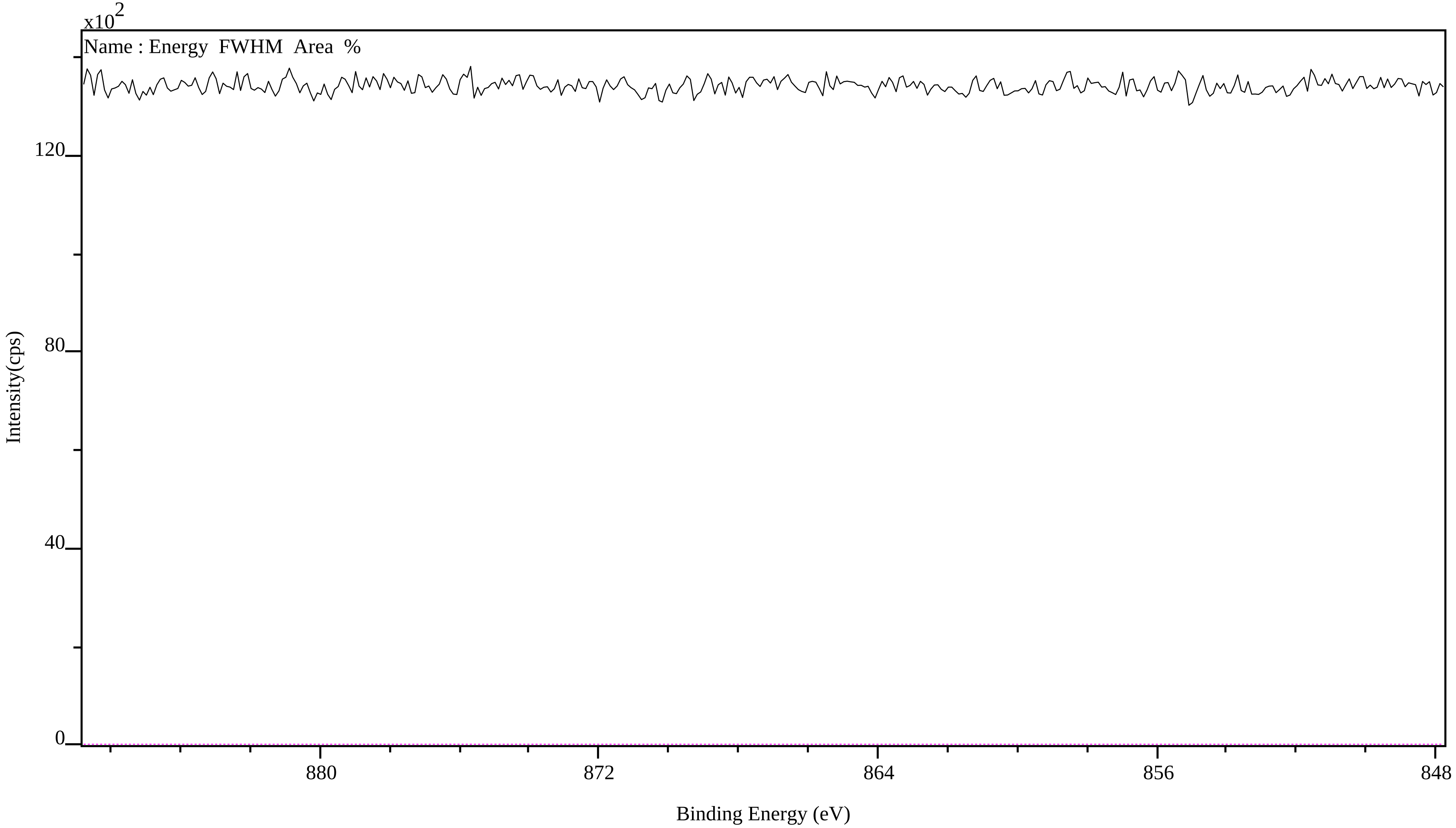

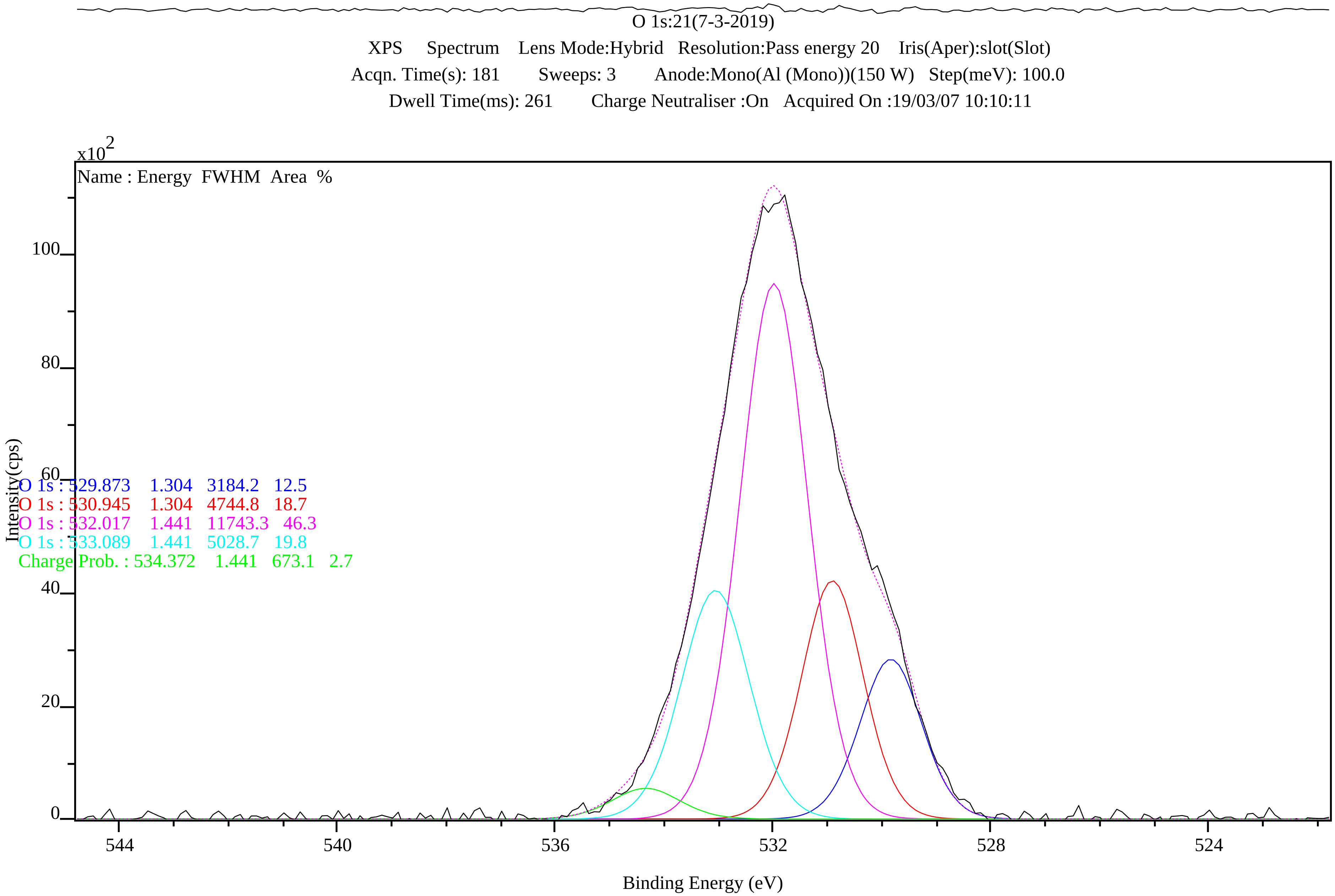
O 1s:21(7-3-2019)
XPS Spectrum Lens Mode:Hybrid Resolution:Pass energy 20 Iris(Aper):slot(Slot)
Acqn. Time(s): 181 Sweeps: 3 Anode:Mono(Al (Mono))(150 W) Step(meV): 100.0
Dwell Time(ms): 261 Charge Neutraliser :On Acquired On :19/03/07 10:10:11
x10^2
Name : Energy FWHM Area %
O 1s : 529.873 1.304 3184.2 12.5
O 1s : 530.945 1.304 4744.8 18.7
O 1s : 532.017 1.441 11743.3 46.3
O 1s : 533.089 1.441 5028.7 19.8
Charge Prob. : 534.372 1.441 673.1 2.7
Intensity(cps)
100
80
60
40
20
0
544
540
536
532
528
524
Binding Energy (eV)

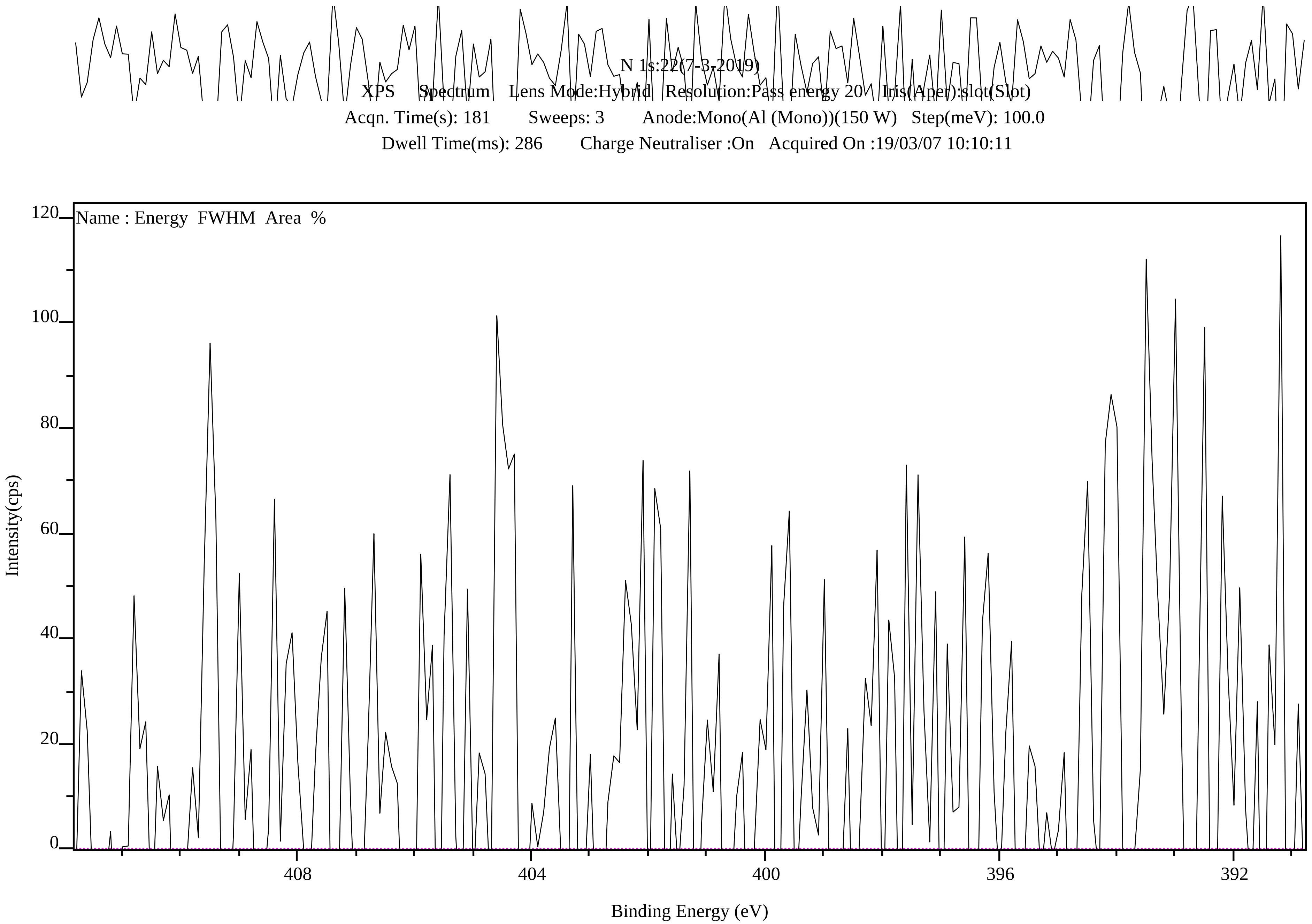
N 1s:22(7-3-2019)
XPS Spectrum Lens Mode:Hybrid Resolution:Pass energy 20 Iris(Aper):slot(Slot)
Acqn. Time(s): 181 Sweeps: 3 Anode:Mono(Al (Mono))(150 W) Step(meV): 100.0
Dwell Time(ms): 286 Charge Neutraliser :On Acquired On :19/03/07 10:10:11
Name : Energy FWHM Area %
Intensity(cps)
120
100
80
60
40
20
0
408
404
400
396
392
Binding Energy (eV)

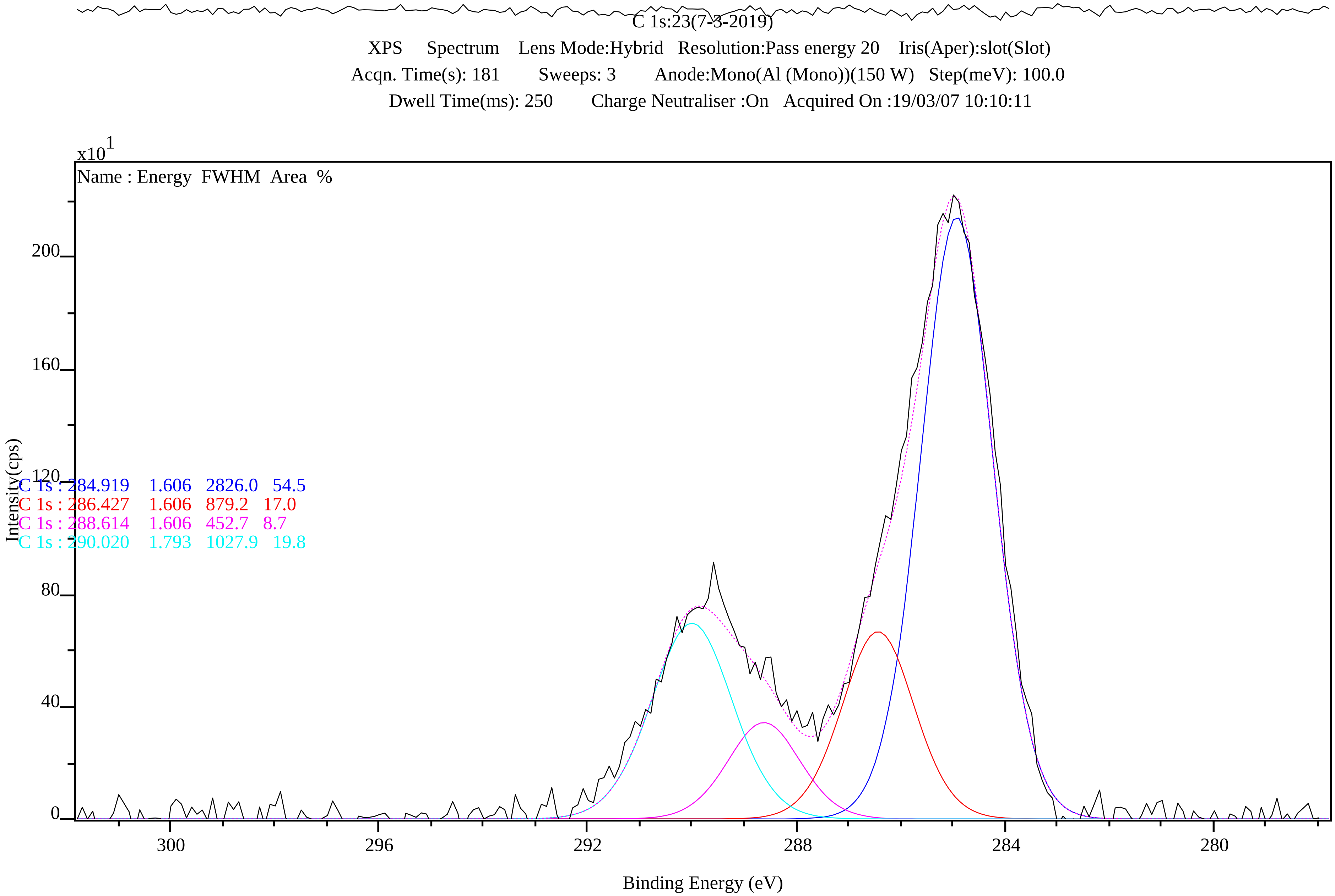

C 1s:23(7-3-2019)
XPS Spectrum Lens Mode:Hybrid Resolution:Pass energy 20 Iris(Aper):slot(Slot)
Acqn. Time(s): 181 Sweeps: 3 Anode:Mono(Al (Mono))(150 W) Step(meV): 100.0
Dwell Time(ms): 250 Charge Neutraliser :On Acquired On :19/03/07 10:10:11
x10^1
Name : Energy FWHM Area %
C 1s : 284.919 1.606 2826.0 54.5
C 1s : 286.427 1.606 879.2 17.0
C 1s : 288.614 1.606 452.7 8.7
C 1s : 290.020 1.793 1027.9 19.8
Intensity(cps)
0
40
80
120
160
200
300
296
292
288
284
280
Binding Energy (eV)
