# Supplementary material for: Influence of the magnetic field on bandgap and chemical composition of zinc thin films prepared by sparking discharge process
Source: Sci Rep. 2020 Jan 29;10:1388. doi: 10.1038/s41598-020-58183-4 (PMC6989455; doi:10.1038/s41598-020-58183-4)

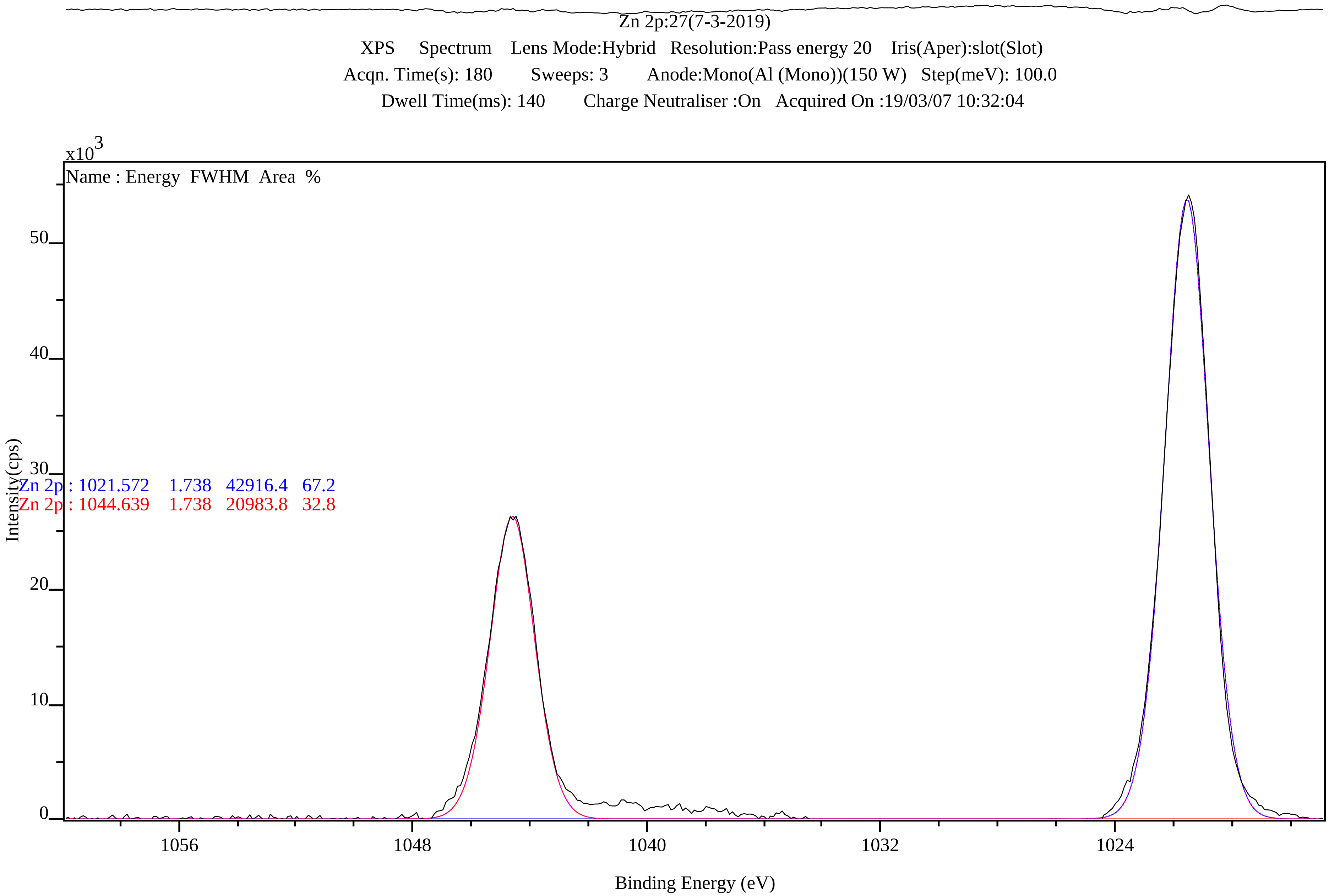
Zn 2p:27(7-3-2019)
XPS Spectrum Lens Mode:Hybrid Resolution:Pass energy 20 Iris(Aper):slot(Slot)
Acqn. Time(s): 180 Sweeps: 3 Anode:Mono(Al (Mono))(150 W) Step(meV): 100.0
Dwell Time(ms): 140 Charge Neutraliser :On Acquired On :19/03/07 10:32:04
x10^3
Name : Energy FWHM Area %
Zn 2p : 1021.572 1.738 42916.4 67.2
Zn 2p : 1044.639 1.738 20983.8 32.8
Intensity(cps)
0
10
20
30
40
50
1056
1048
1040
1032
1024
Binding Energy (eV)

Ni 2p:28(7-3-2019)

XPS Spectrum Lens Mode:Hybrid Resolution:Pass energy 20 Iris(Aper):slot(Slot)

Acqn. Time(s): 180 Sweeps: 3 Anode:Mono(Al (Mono))(150 W) Step(meV): 100.0

Dwell Time(ms): 154 Charge Neutraliser :On Acquired On :19/03/07 10:32:04

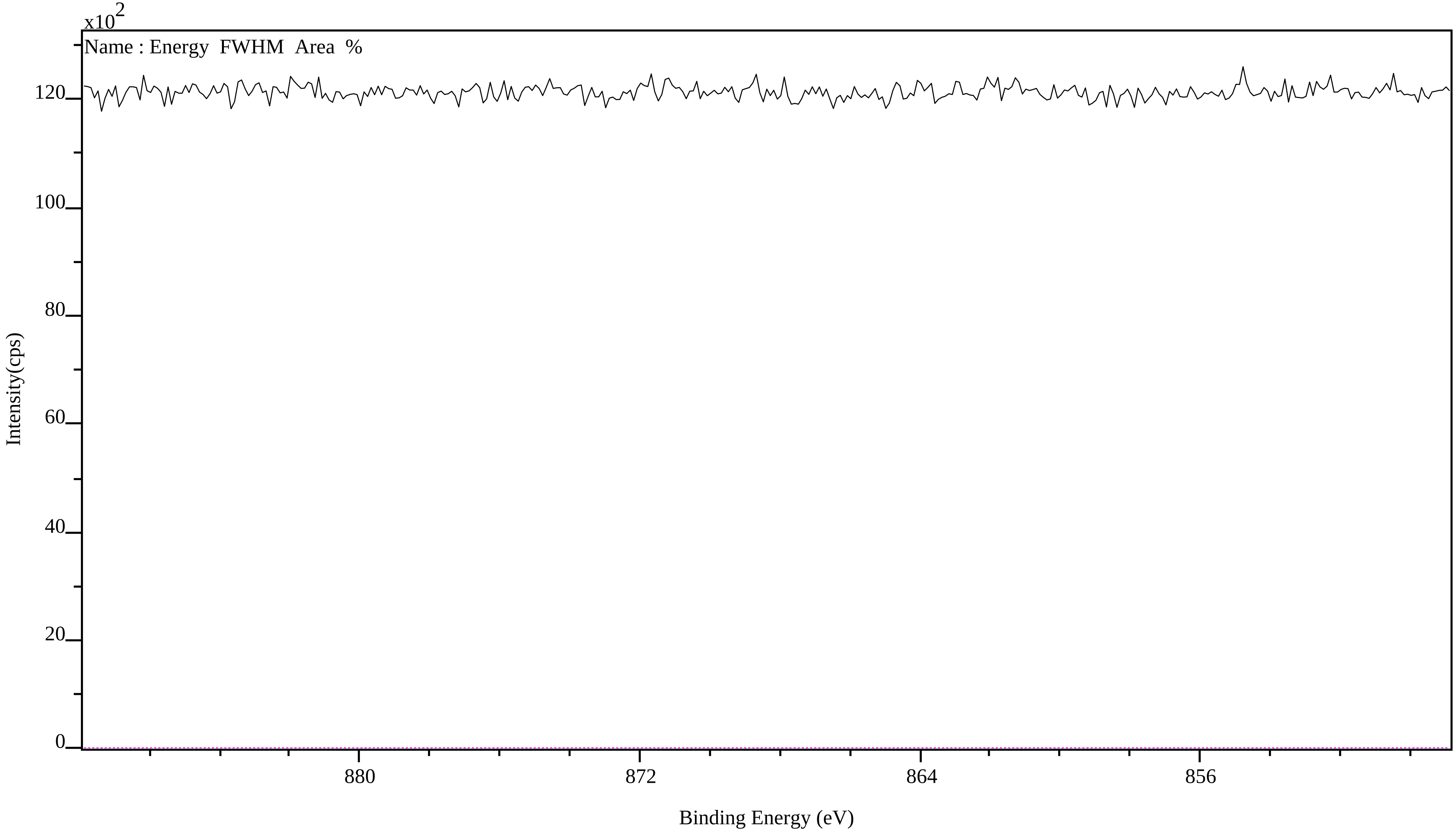

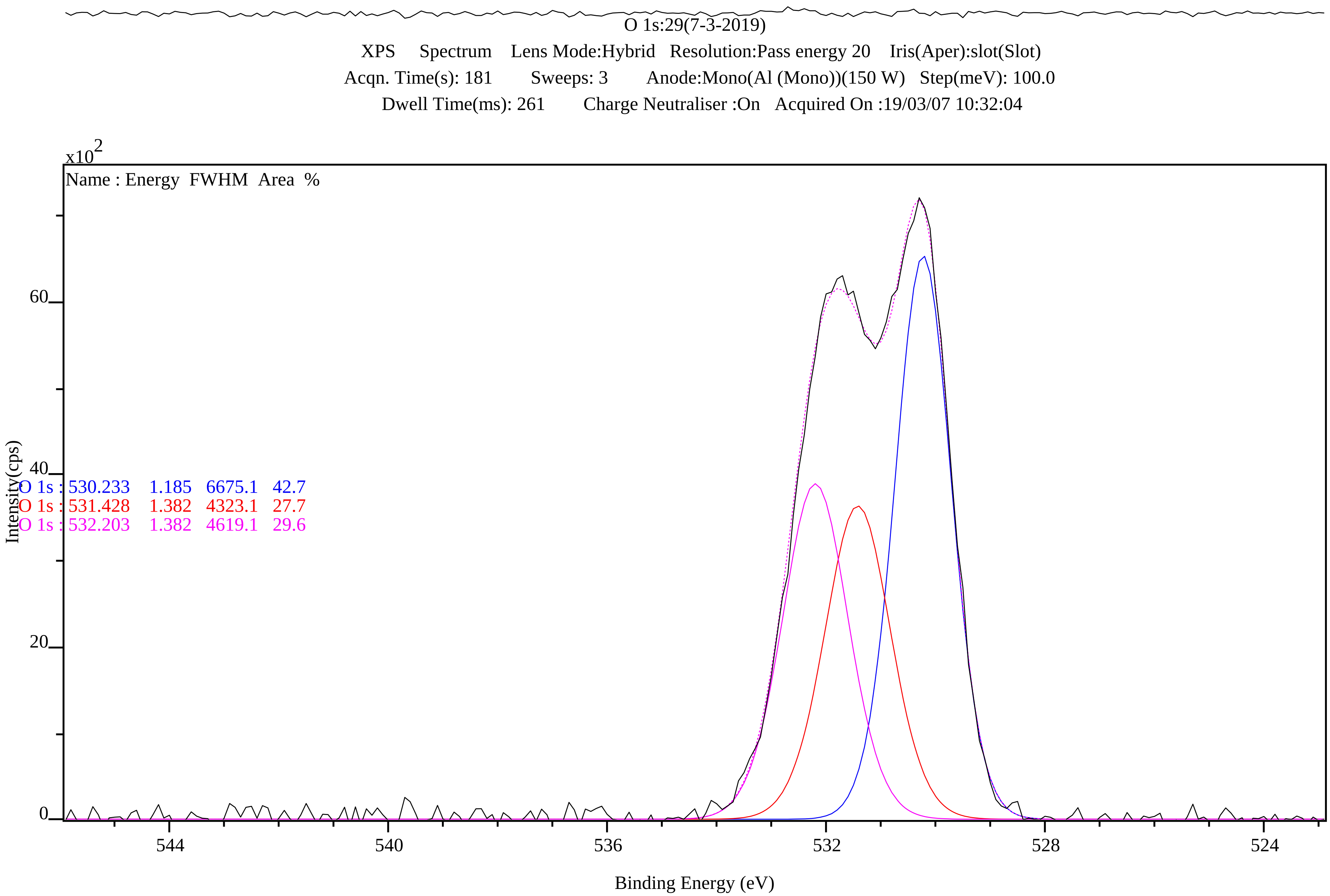
O 1s:29(7-3-2019)
XPS Spectrum Lens Mode:Hybrid Resolution:Pass energy 20 Iris(Aper):slot(Slot)
Acqn. Time(s): 181 Sweeps: 3 Anode:Mono(Al (Mono))(150 W) Step(meV): 100.0
Dwell Time(ms): 261 Charge Neutraliser :On Acquired On :19/03/07 10:32:04
x10^2
Name : Energy FWHM Area %
O 1s : 530.233 1.185 6675.1 42.7
O 1s : 531.428 1.382 4323.1 27.7
O 1s : 532.203 1.382 4619.1 29.6
Intensity(cps)
60
40
20
0
544
540
536
532
528
524
Binding Energy (eV)

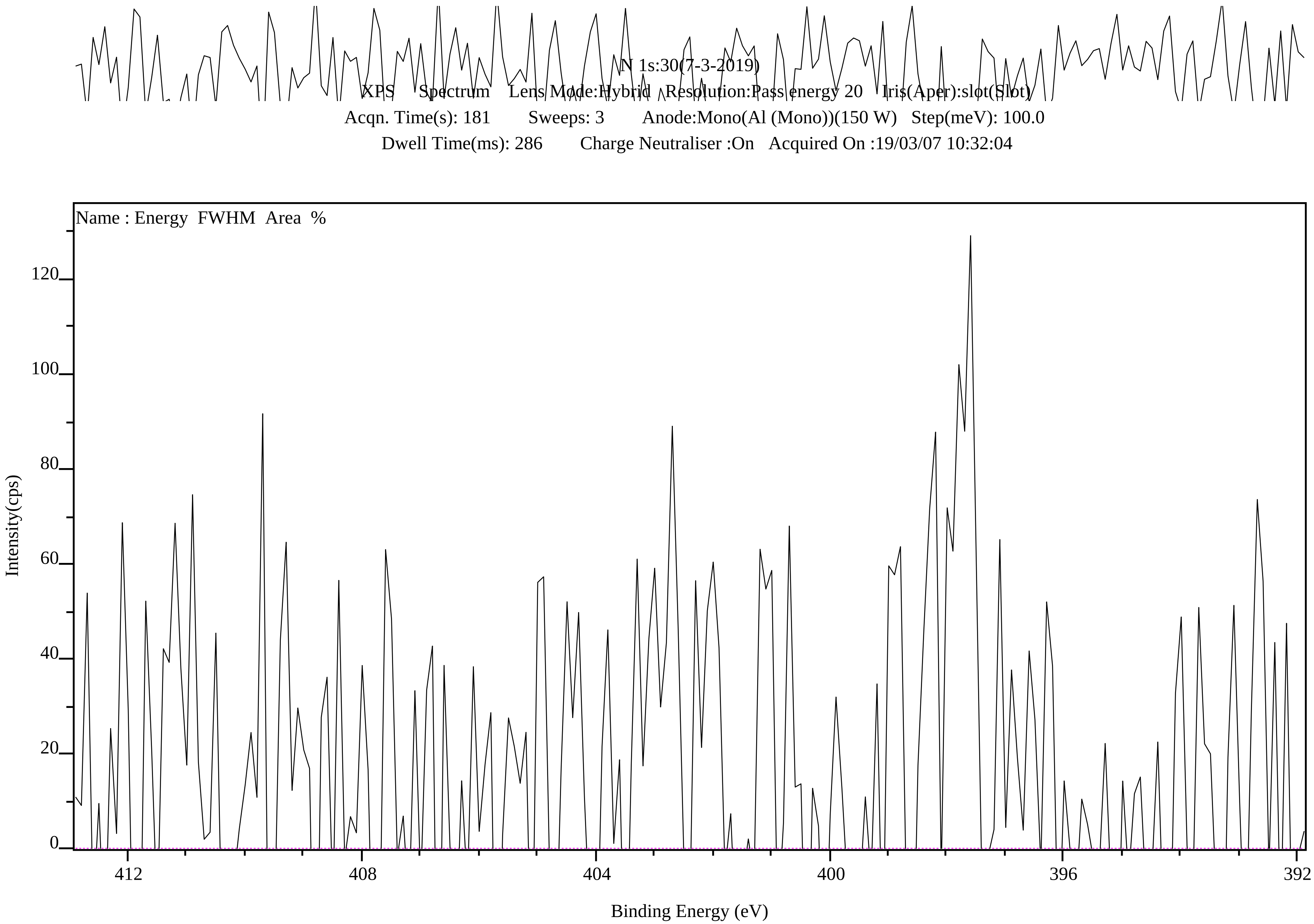
N 1s:30(7-3-2019)
XPS Spectrum Lens Mode:Hybrid Resolution:Pass energy 20 Iris(Aper):slot(Slot)
Acqn. Time(s): 181 Sweeps: 3 Anode:Mono(Al (Mono))(150 W) Step(meV): 100.0
Dwell Time(ms): 286 Charge Neutraliser :On Acquired On :19/03/07 10:32:04
Name : Energy FWHM Area %
Intensity(cps)
0
20
40
60
80
100
120
412
408
404
400
396
392
Binding Energy (eV)

C 1s:31(7-3-2019)

XPS Spectrum Lens Mode:Hybrid Resolution:Pass energy 20 Iris(Aper):slot(Slot)

Acqn. Time(s): 181 Sweeps: 3 Anode:Mono(Al (Mono))(150 W) Step(meV): 100.0

Dwell Time(ms): 250 Charge Neutraliser :On Acquired On :19/03/07 10:32:04

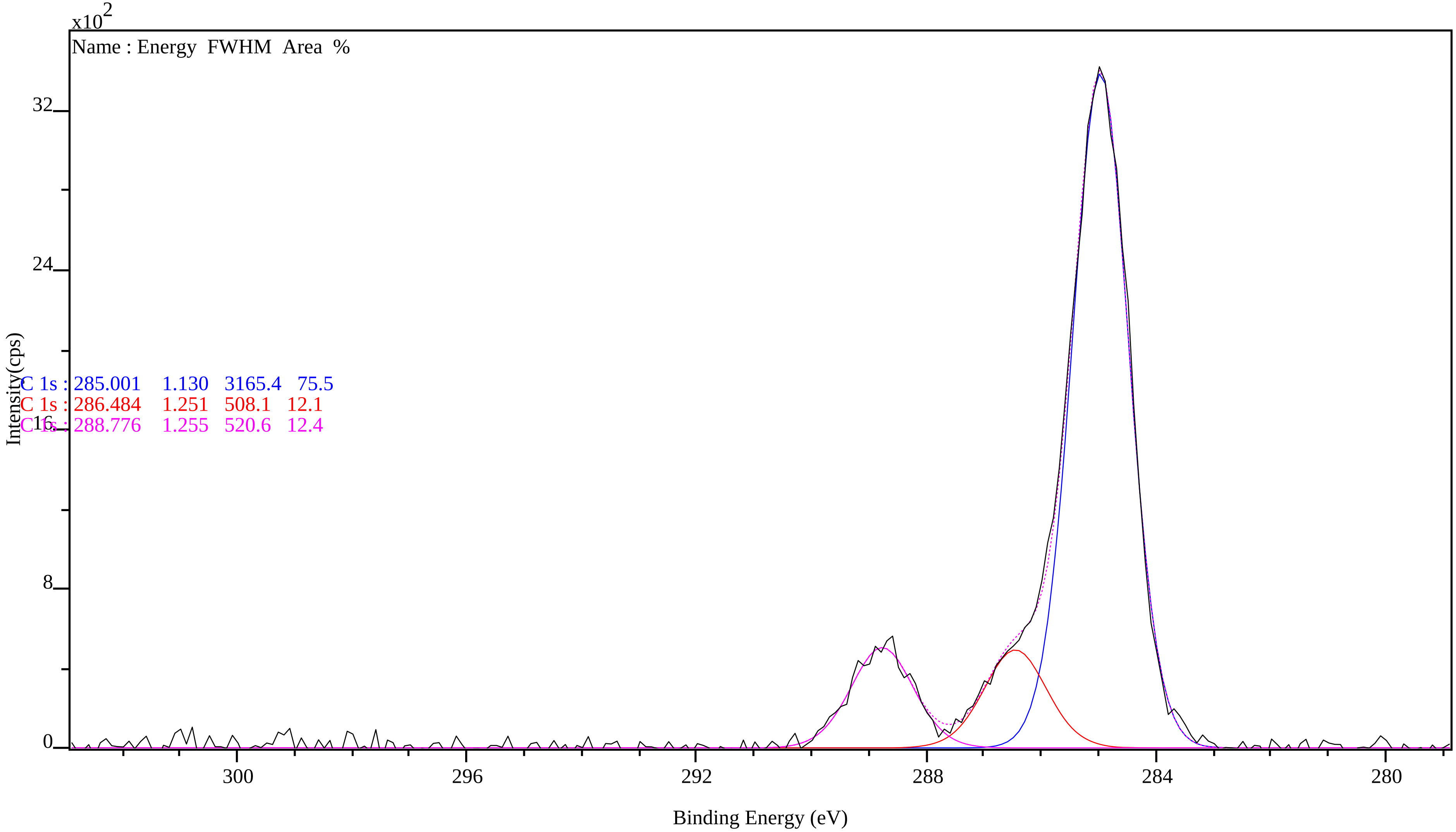

Supplement: Supplementary file 5 — Related Manuscript File. [file 41598_2020_58183_MOESM5_ESM.zip › XPS_7-3-2019_Stefan/XPS_7-3-2019/4_ZnN2-0.4T_ITO/component.pdf]
