# Supplementary material for: Influence of the magnetic field on bandgap and chemical composition of zinc thin films prepared by sparking discharge process
Source: Sci Rep. 2020 Jan 29;10:1388. doi: 10.1038/s41598-020-58183-4 (PMC6989455; doi:10.1038/s41598-020-58183-4)

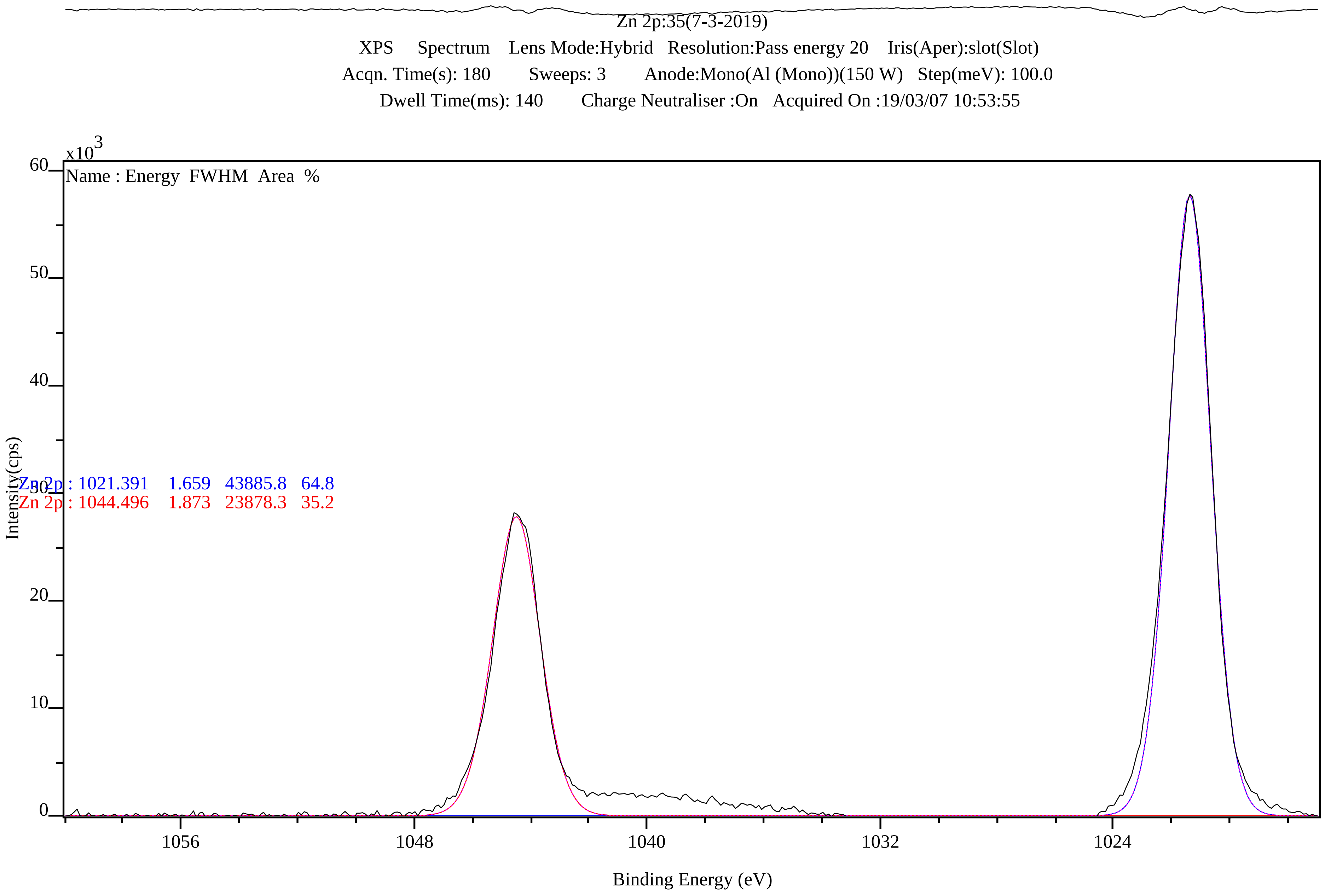

Zn 2p:35(7-3-2019)
XPS Spectrum Lens Mode:Hybrid Resolution:Pass energy 20 Iris(Aper):slot(Slot)
Acqn. Time(s): 180 Sweeps: 3 Anode:Mono(Al (Mono))(150 W) Step(meV): 100.0
Dwell Time(ms): 140 Charge Neutraliser :On Acquired On :19/03/07 10:53:55
x10^3
Name : Energy FWHM Area %
Zn 2p : 1021.391 1.659 43885.8 64.8
Zn 2p : 1044.496 1.873 23878.3 35.2
Intensity(cps)
60
50
40
30
20
10
0
1056
1048
1040
1032
1024
Binding Energy (eV)

Ni 2p:36(7-3-2019)

XPS Spectrum Lens Mode:Hybrid Resolution:Pass energy 20 Iris(Aper):slot(Slot)

Acqn. Time(s): 180 Sweeps: 3 Anode:Mono(Al (Mono))(150 W) Step(meV): 100.0

Dwell Time(ms): 154 Charge Neutraliser :On Acquired On :19/03/07 10:53:55

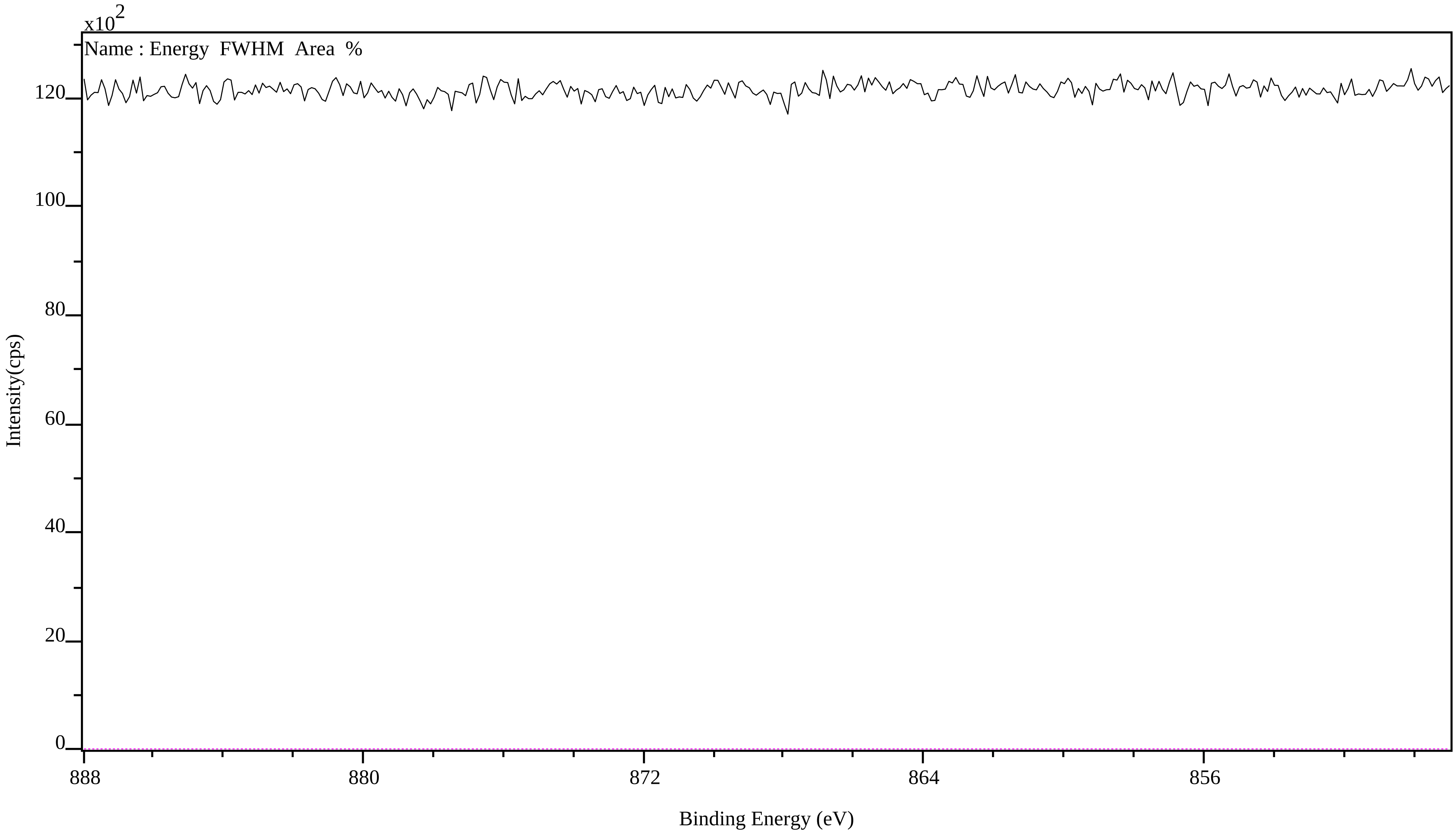

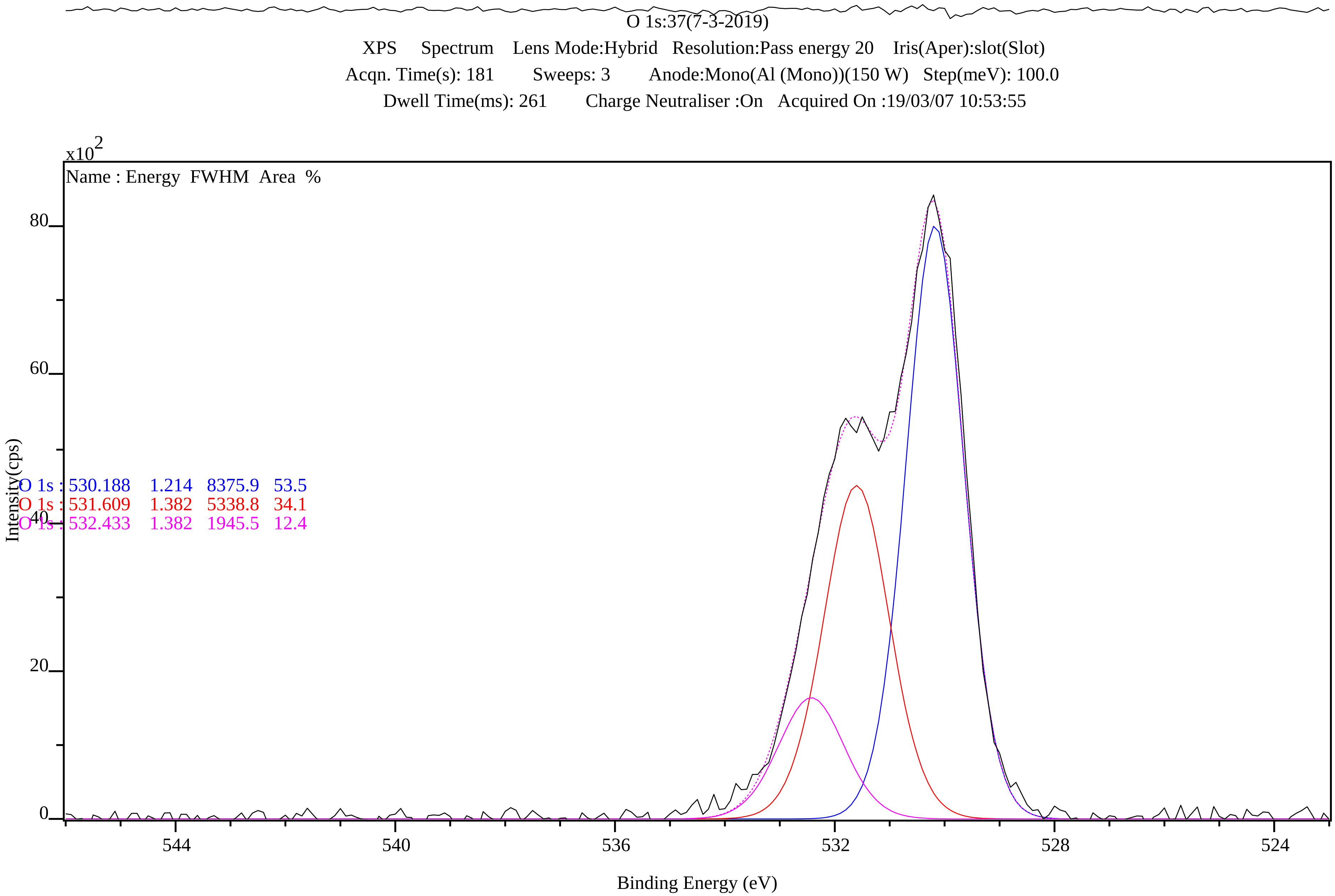

O 1s:37(7-3-2019)
XPS Spectrum Lens Mode:Hybrid Resolution:Pass energy 20 Iris(Aper):slot(Slot)
Acqn. Time(s): 181 Sweeps: 3 Anode:Mono(Al (Mono))(150 W) Step(meV): 100.0
Dwell Time(ms): 261 Charge Neutraliser :On Acquired On :19/03/07 10:53:55
x10^2
Name : Energy FWHM Area %
O 1s : 530.188 1.214 8375.9 53.5
O 1s : 531.609 1.382 5338.8 34.1
O 1s : 532.433 1.382 1945.5 12.4
Intensity(cps)
0
20
40
60
80
544
540
536
532
528
524
Binding Energy (eV)

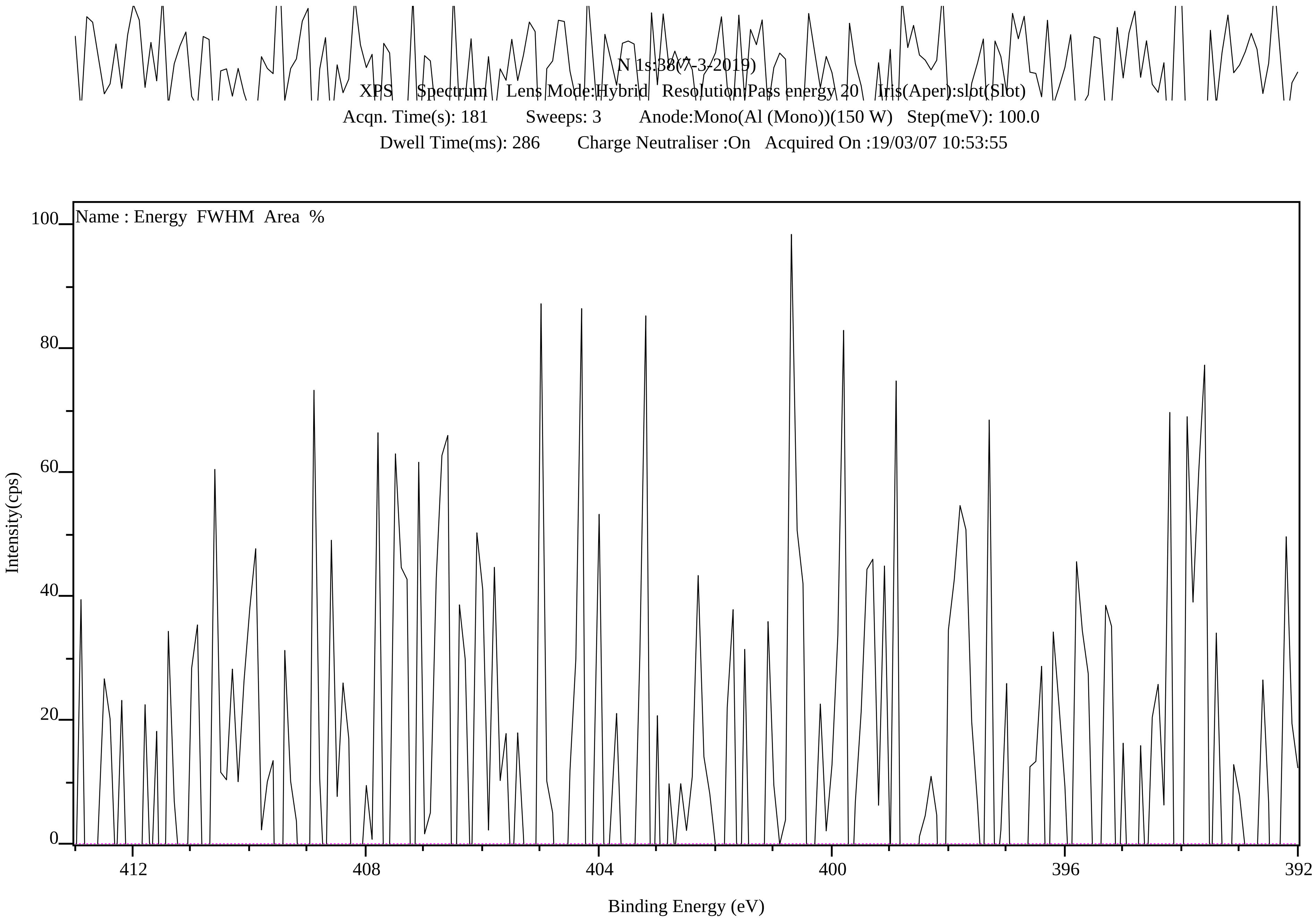
N 1s:38(7-3-2019)
XPS Spectrum Lens Mode:Hybrid Resolution:Pass energy 20 Iris(Aper):slot(Slot)
Acqn. Time(s): 181 Sweeps: 3 Anode:Mono(Al (Mono))(150 W) Step(meV): 100.0
Dwell Time(ms): 286 Charge Neutraliser :On Acquired On :19/03/07 10:53:55
Name : Energy FWHM Area %
Intensity(cps)
100
80
60
40
20
0
412
408
404
400
396
392
Binding Energy (eV)

C 1s:39(7-3-2019)

XPS Spectrum Lens Mode:Hybrid Resolution:Pass energy 20 Iris(Aper):slot(Slot)

Acqn. Time(s): 181 Sweeps: 3 Anode:Mono(Al (Mono))(150 W) Step(meV): 100.0

Dwell Time(ms): 250 Charge Neutraliser :On Acquired On :19/03/07 10:53:55

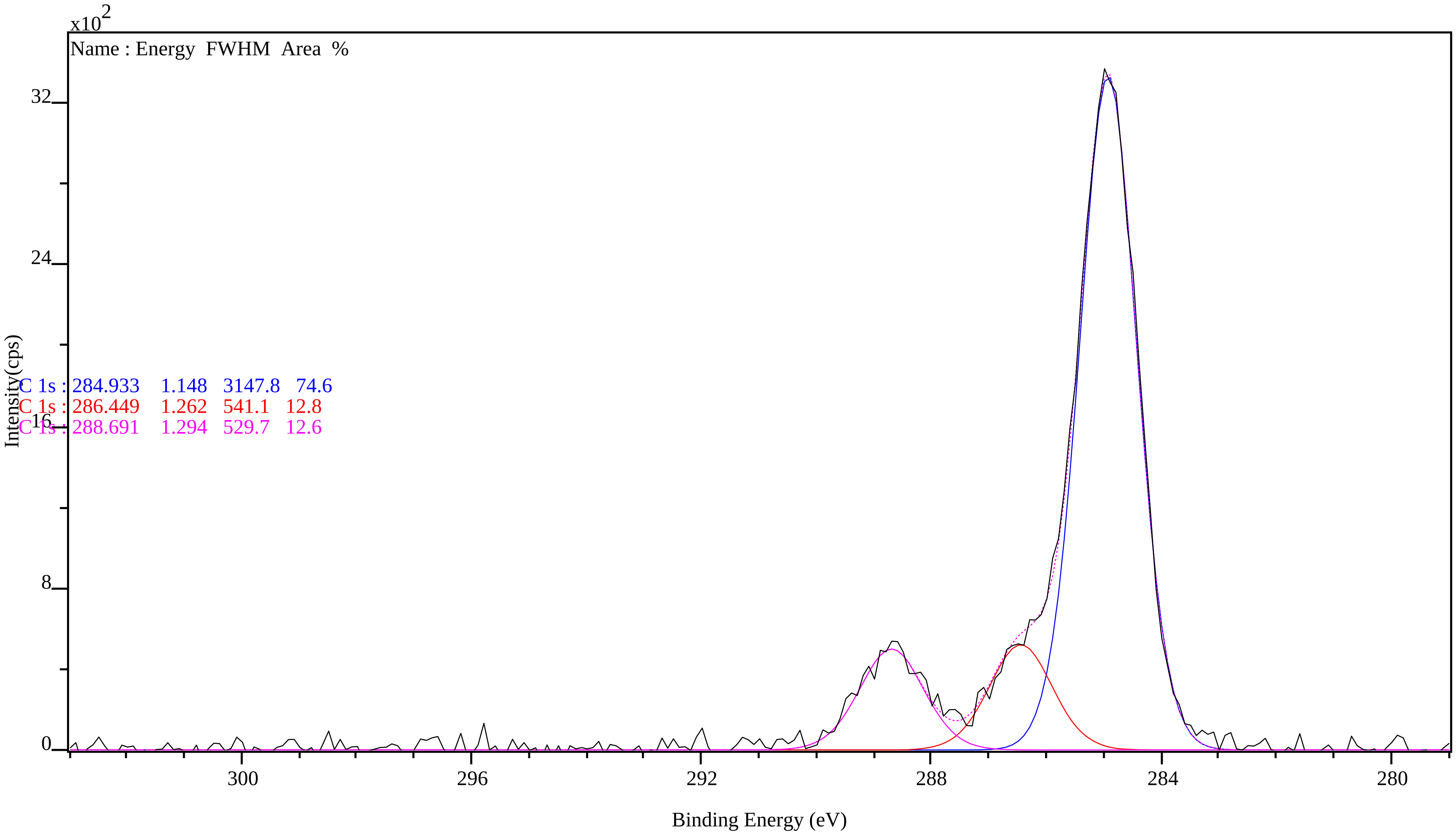

Supplement: Supplementary file 5 — Related Manuscript File. [file 41598_2020_58183_MOESM5_ESM.zip › XPS_7-3-2019_Stefan/XPS_7-3-2019/5_ZnN2-0.4_Quartz/component.pdf]
