# Supplementary material for: Influence of the magnetic field on bandgap and chemical composition of zinc thin films prepared by sparking discharge process
Source: Sci Rep. 2020 Jan 29;10:1388. doi: 10.1038/s41598-020-58183-4 (PMC6989455; doi:10.1038/s41598-020-58183-4)

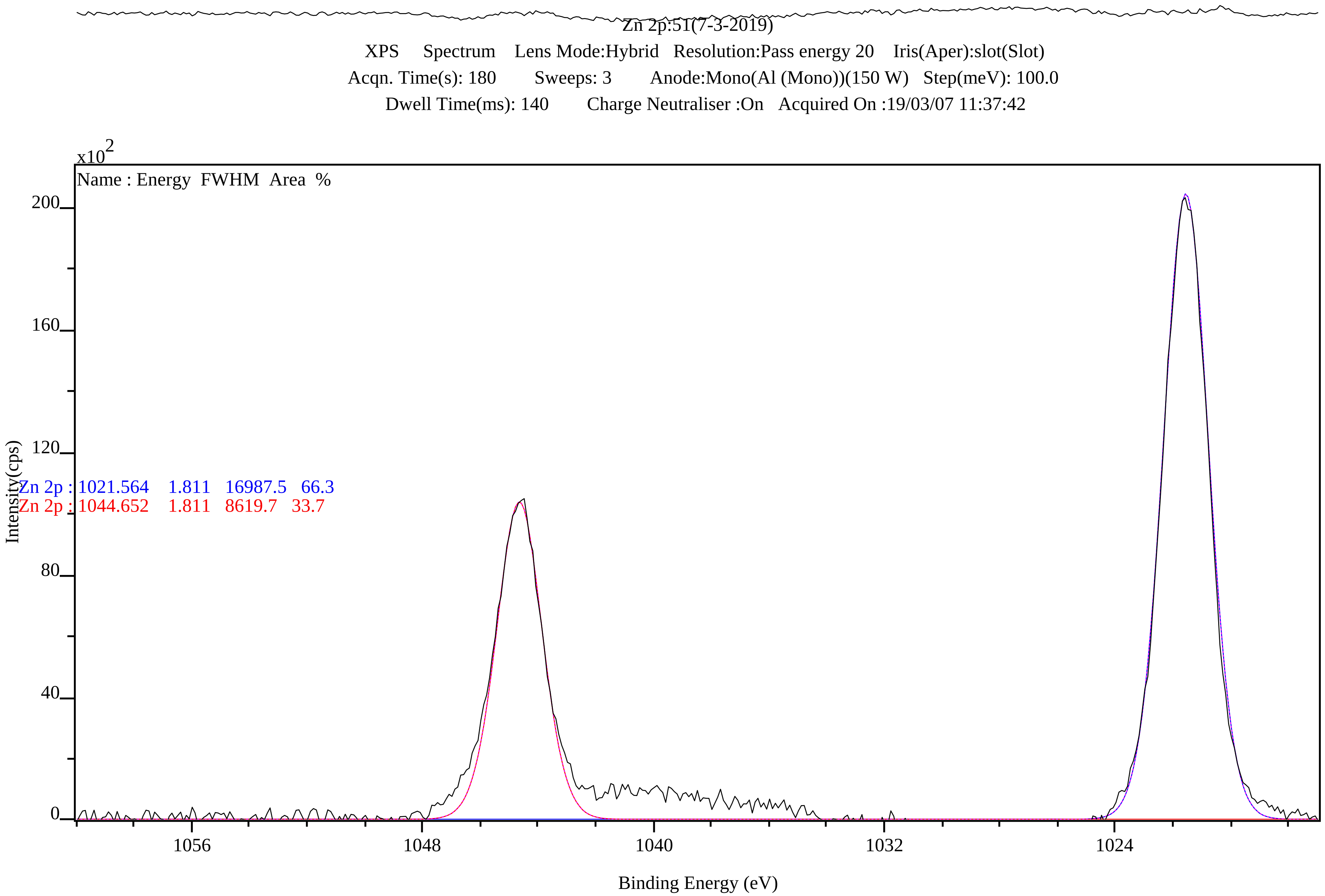

Zn 2p:51(7-3-2019)
XPS Spectrum Lens Mode:Hybrid Resolution:Pass energy 20 Iris(Aper):slot(Slot)
Acqn. Time(s): 180 Sweeps: 3 Anode:Mono(Al (Mono))(150 W) Step(meV): 100.0
Dwell Time(ms): 140 Charge Neutraliser :On Acquired On :19/03/07 11:37:42
x10^2
Name : Energy FWHM Area %
Zn 2p : 1021.564 1.811 16987.5 66.3
Zn 2p : 1044.652 1.811 8619.7 33.7
Intensity(cps)
200
160
120
80
40
0
1056
1048
1040
1032
1024
Binding Energy (eV)

Ni 2p:52(7-3-2019)

XPS Spectrum Lens Mode:Hybrid Resolution:Pass energy 20 Iris(Aper):slot(Slot)

Acqn. Time(s): 180 Sweeps: 3 Anode:Mono(Al (Mono))(150 W) Step(meV): 100.0

Dwell Time(ms): 154 Charge Neutraliser :On Acquired On :19/03/07 11:37:42

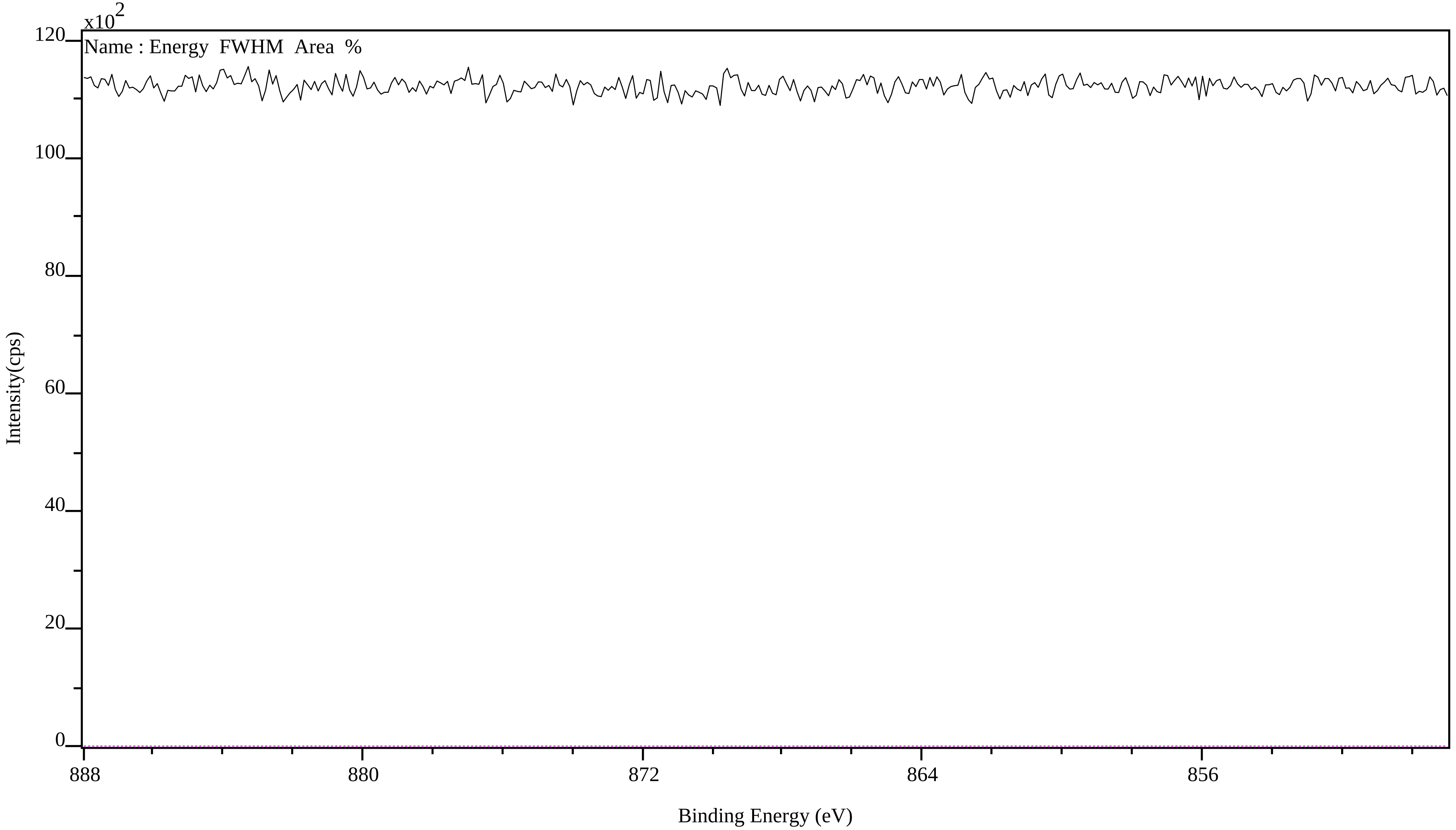

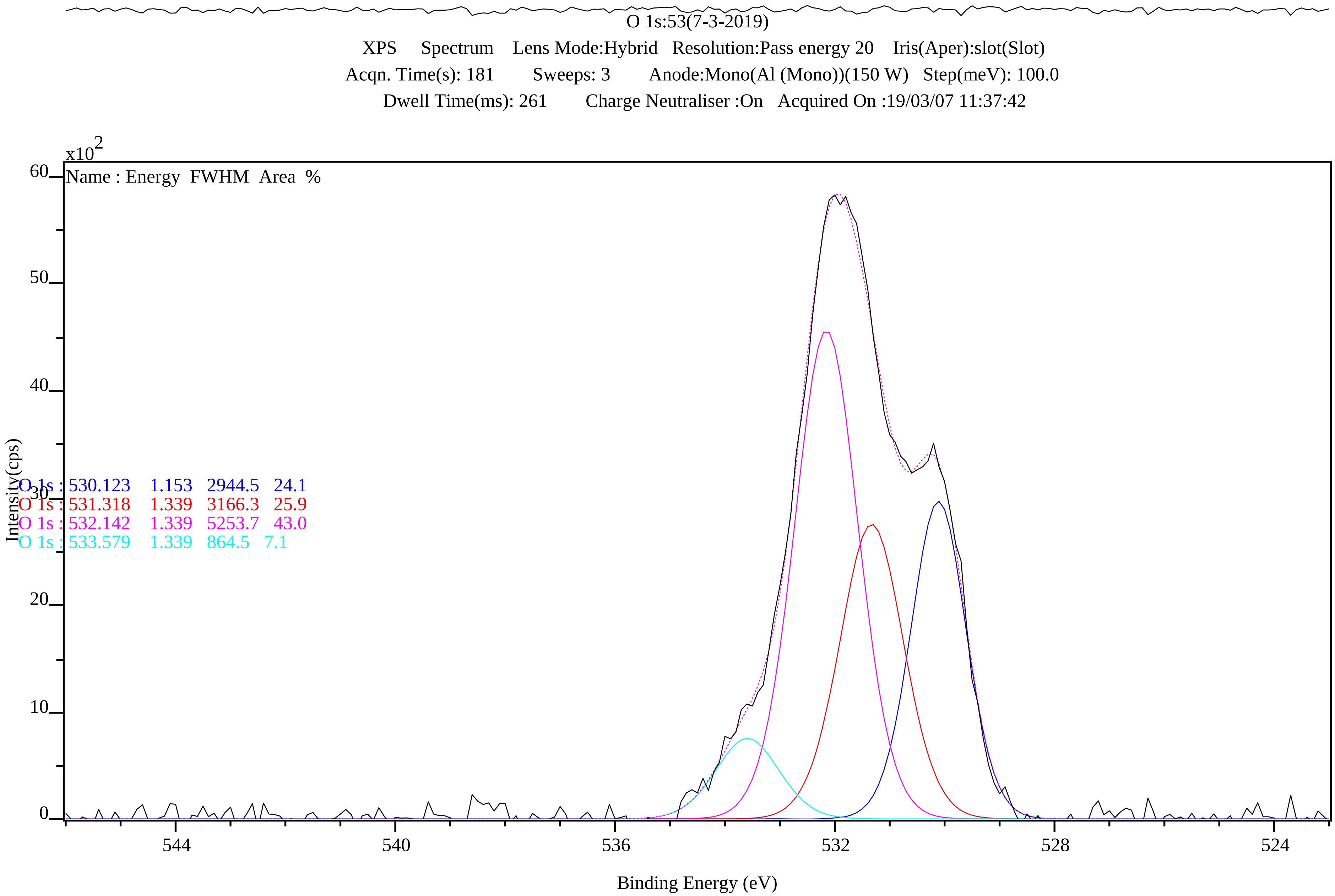

O 1s:53(7-3-2019)
XPS Spectrum Lens Mode:Hybrid Resolution:Pass energy 20 Iris(Aper):slot(Slot)
Acqn. Time(s): 181 Sweeps: 3 Anode:Mono(Al (Mono))(150 W) Step(meV): 100.0
Dwell Time(ms): 261 Charge Neutraliser :On Acquired On :19/03/07 11:37:42
x10^2
Name : Energy FWHM Area %
O 1s : 530.123 1.153 2944.5 24.1
O 1s : 531.318 1.339 3166.3 25.9
O 1s : 532.142 1.339 5253.7 43.0
O 1s : 533.579 1.339 864.5 7.1
Intensity(cps)
60
50
40
30
20
10
0
544
540
536
532
528
524
Binding Energy (eV)

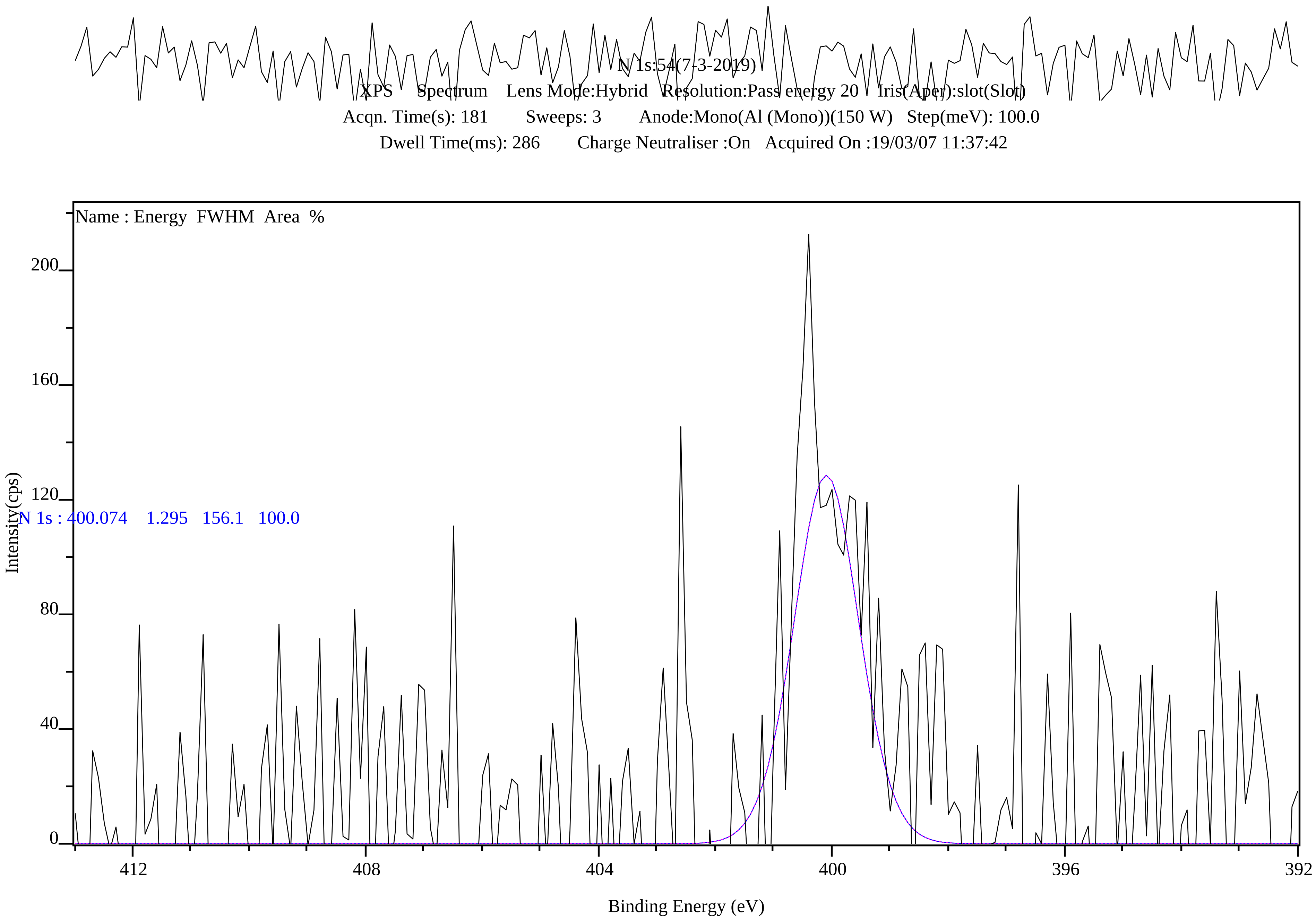
N 1s:54(7-3-2019)
XPS Spectrum Lens Mode:Hybrid Resolution:Pass energy 20 Iris(Aper):slot(Slot)
Acqn. Time(s): 181 Sweeps: 3 Anode:Mono(Al (Mono))(150 W) Step(meV): 100.0
Dwell Time(ms): 286 Charge Neutraliser :On Acquired On :19/03/07 11:37:42
Name : Energy FWHM Area %
N 1s : 400.074 1.295 156.1 100.0
Intensity(cps)
200
160
120
80
40
0
412
408
404
400
396
392
Binding Energy (eV)

C 1s:55(7-3-2019)

XPS Spectrum Lens Mode:Hybrid Resolution:Pass energy 20 Iris(Aper):slot(Slot)

Acqn. Time(s): 181 Sweeps: 3 Anode:Mono(Al (Mono))(150 W) Step(meV): 100.0

Dwell Time(ms): 250 Charge Neutraliser :On Acquired On :19/03/07 11:37:42

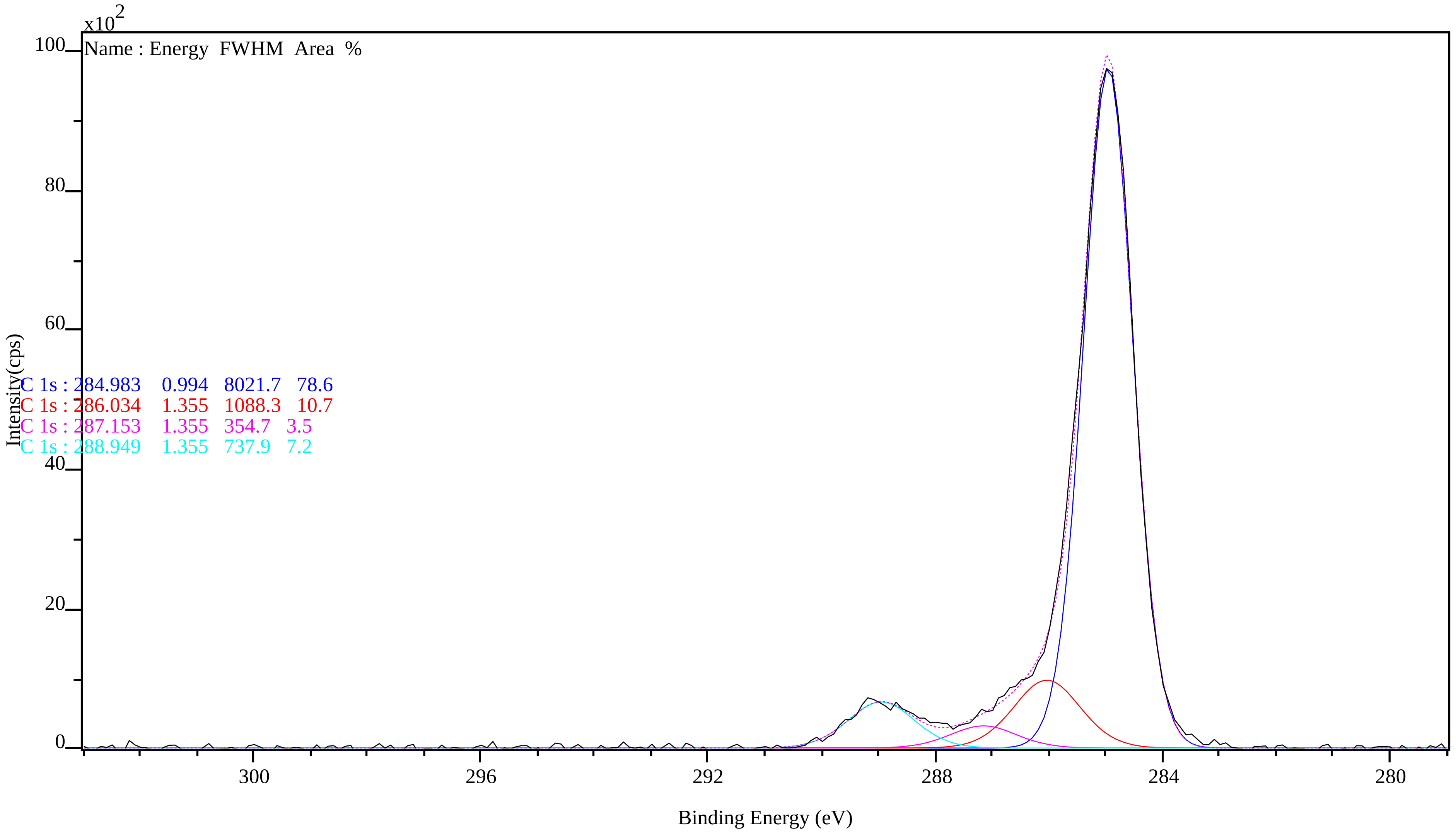

Supplement: Supplementary file 5 — Related Manuscript File. [file 41598_2020_58183_MOESM5_ESM.zip › XPS_7-3-2019_Stefan/XPS_7-3-2019/7_ZnN2-0.2_Quartz/component.pdf]
