# Supplementary material for: Influence of the magnetic field on bandgap and chemical composition of zinc thin films prepared by sparking discharge process
Source: Sci Rep. 2020 Jan 29;10:1388. doi: 10.1038/s41598-020-58183-4 (PMC6989455; doi:10.1038/s41598-020-58183-4)

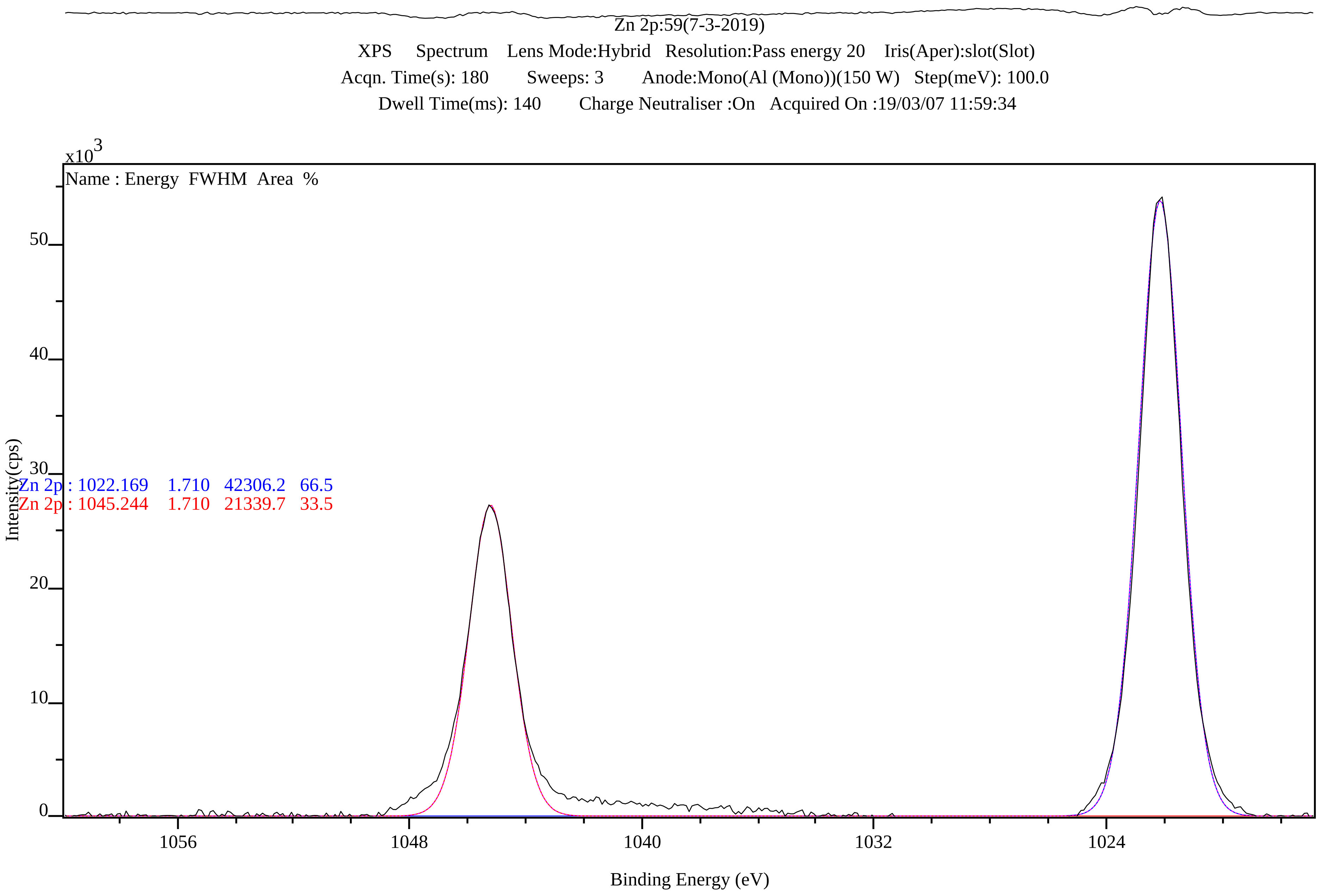
Zn 2p:59(7-3-2019)
XPS Spectrum Lens Mode:Hybrid Resolution:Pass energy 20 Iris(Aper):slot(Slot)
Acqn. Time(s): 180 Sweeps: 3 Anode:Mono(Al (Mono))(150 W) Step(meV): 100.0
Dwell Time(ms): 140 Charge Neutraliser :On Acquired On :19/03/07 11:59:34
x10^3
Name : Energy FWHM Area %
Zn 2p : 1022.169 1.710 42306.2 66.5
Zn 2p : 1045.244 1.710 21339.7 33.5
Intensity(cps)
50
40
30
20
10
0
1056
1048
1040
1032
1024
Binding Energy (eV)

Ni 2p:60(7-3-2019)

XPS Spectrum Lens Mode:Hybrid Resolution:Pass energy 20 Iris(Aper):slot(Slot)

Acqn. Time(s): 180 Sweeps: 3 Anode:Mono(Al (Mono))(150 W) Step(meV): 100.0

Dwell Time(ms): 154 Charge Neutraliser :On Acquired On :19/03/07 11:59:34

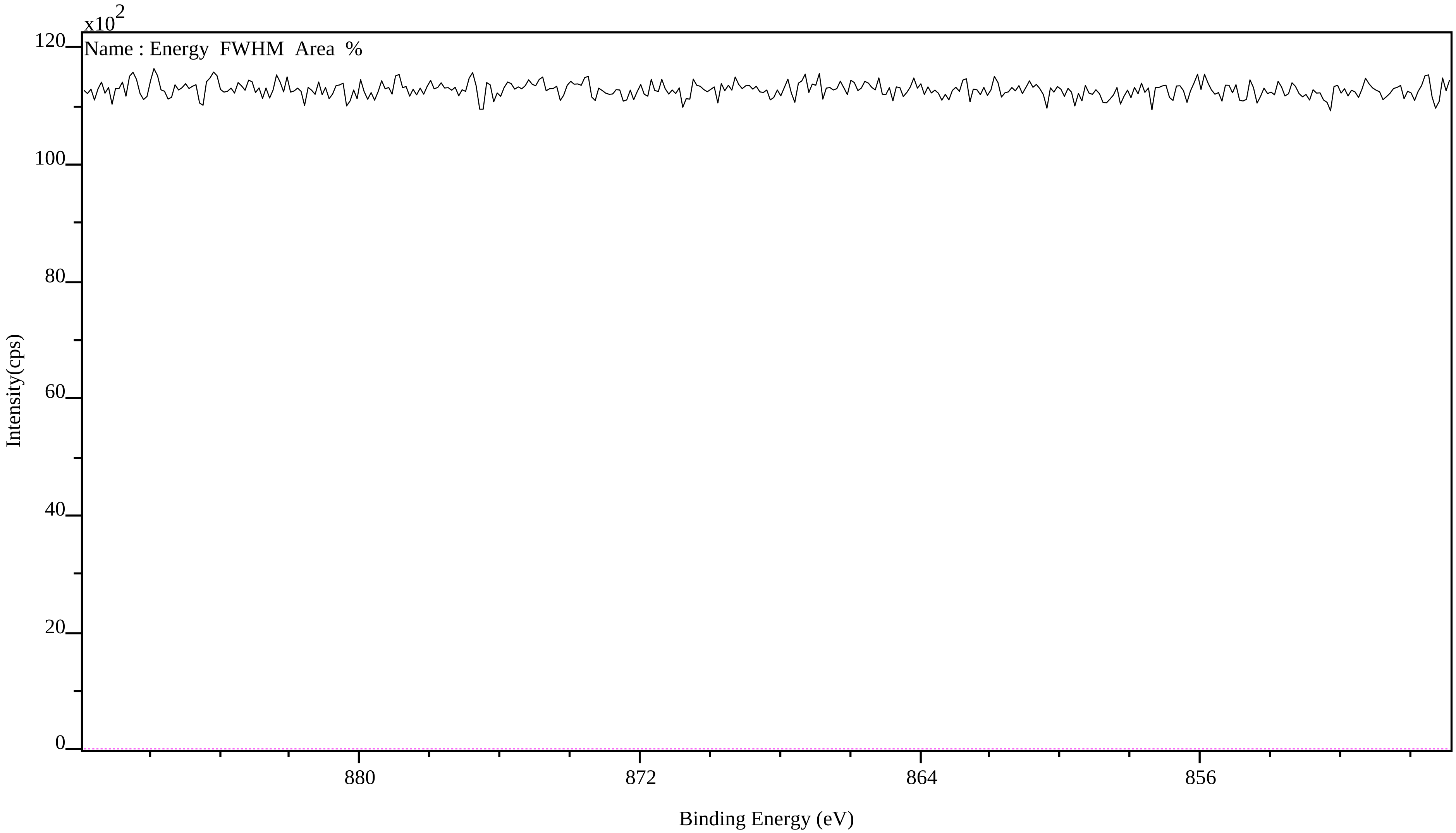

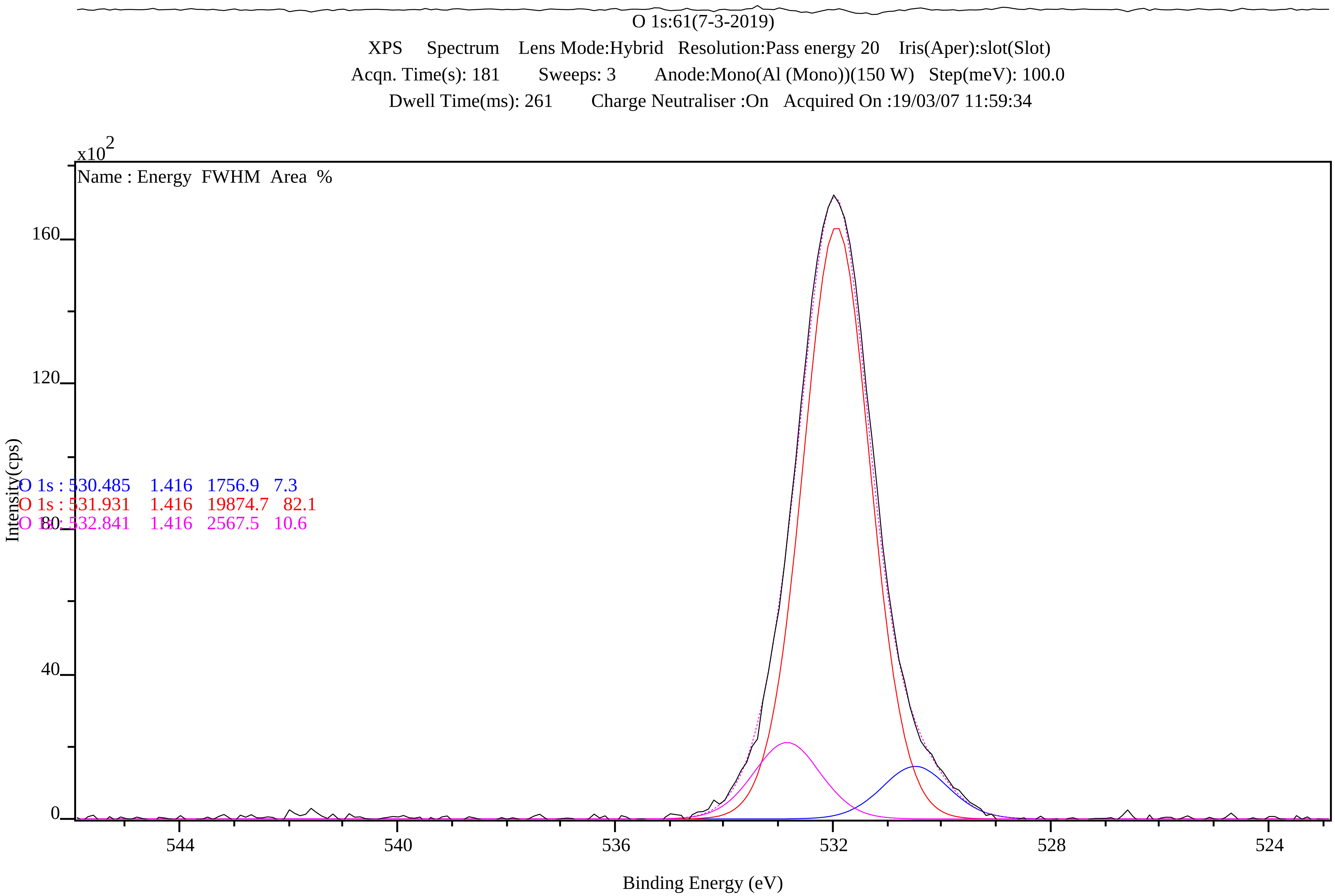

O 1s:61(7-3-2019)
XPS Spectrum Lens Mode:Hybrid Resolution:Pass energy 20 Iris(Aper):slot(Slot)
Acqn. Time(s): 181 Sweeps: 3 Anode:Mono(Al (Mono))(150 W) Step(meV): 100.0
Dwell Time(ms): 261 Charge Neutraliser :On Acquired On :19/03/07 11:59:34
x10^2
Name : Energy FWHM Area %
O 1s : 530.485 1.416 1756.9 7.3
O 1s : 531.931 1.416 19874.7 82.1
O 1s : 532.841 1.416 2567.5 10.6
Intensity(cps)
160
120
80
40
0
544
540
536
532
528
524
Binding Energy (eV)

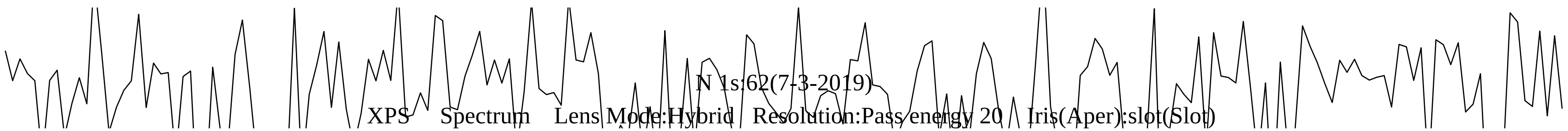

N 1s:62(7-3-2019)

XPS Spectrum Lens Mode:Hybrid Resolution:Pass energy 20 Iris(Aper):slot(Slot)

Acqn. Time(s): 181 Sweeps: 3 Anode:Mono(Al (Mono))(150 W) Step(meV): 100.0

Dwell Time(ms): 286 Charge Neutraliser :On Acquired On :19/03/07 11:59:34

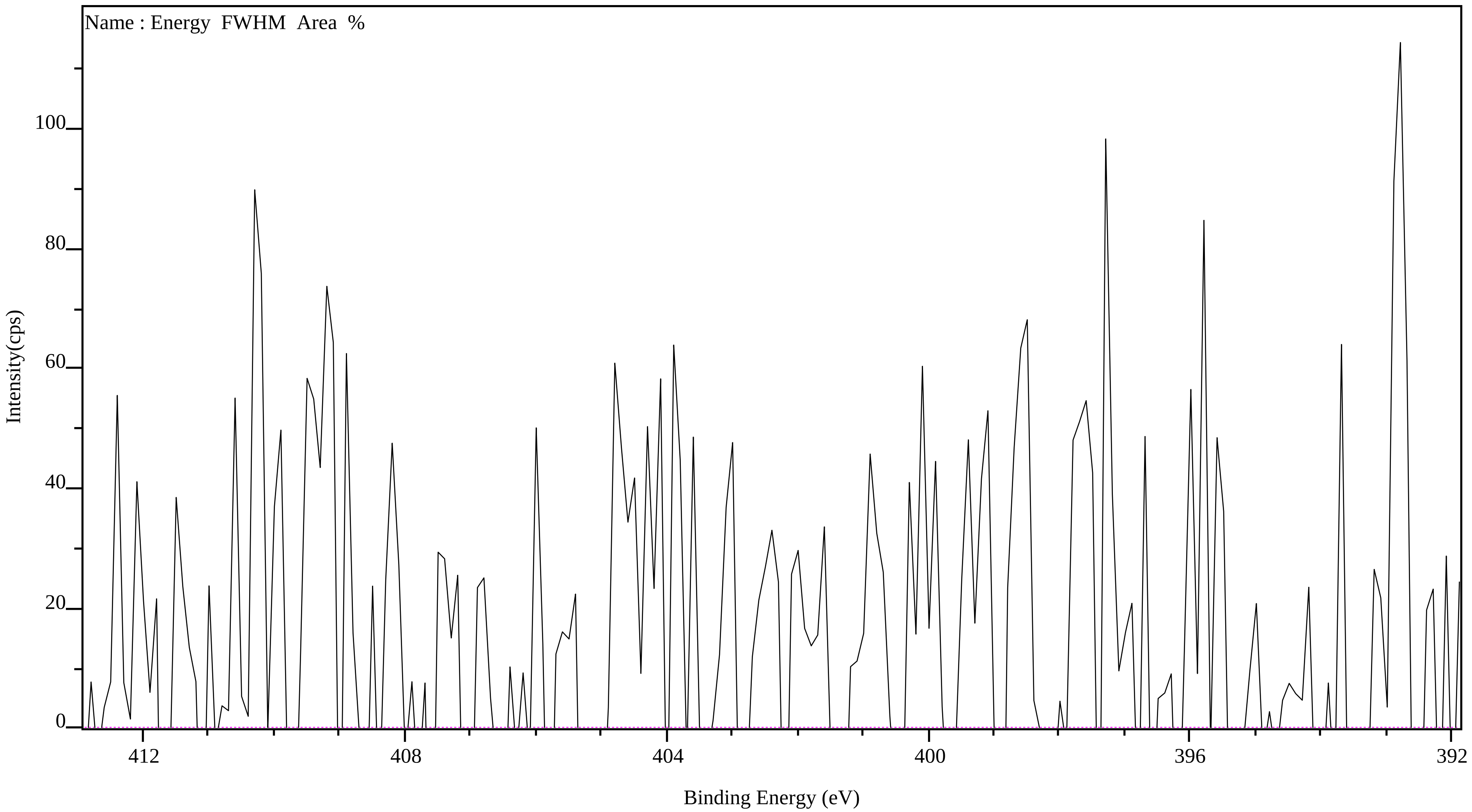

C 1s:63(7-3-2019)

XPS Spectrum Lens Mode:Hybrid Resolution:Pass energy 20 Iris(Aper):slot(Slot)

Acqn. Time(s): 181 Sweeps: 3 Anode:Mono(Al (Mono))(150 W) Step(meV): 100.0

Dwell Time(ms): 250 Charge Neutraliser :On Acquired On :19/03/07 11:59:34

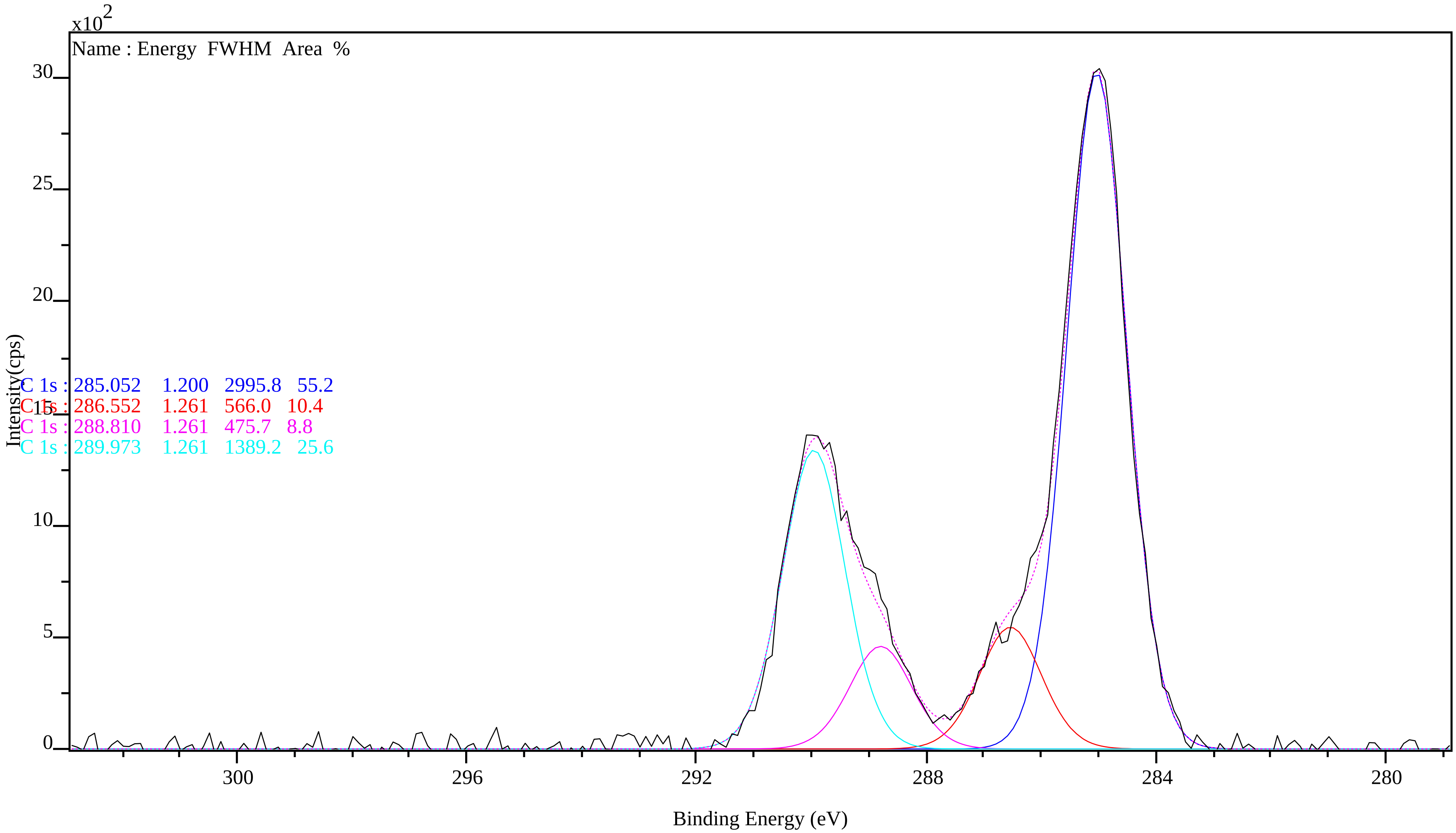

Supplement: Supplementary file 5 — Related Manuscript File. [file 41598_2020_58183_MOESM5_ESM.zip › XPS_7-3-2019_Stefan/XPS_7-3-2019/8_ZnCO2-0.2_Quartz/component.pdf]
