# Supplementary material for: Influence of the magnetic field on bandgap and chemical composition of zinc thin films prepared by sparking discharge process
Source: Sci Rep. 2020 Jan 29;10:1388. doi: 10.1038/s41598-020-58183-4 (PMC6989455; doi:10.1038/s41598-020-58183-4)
Supplement: Supplementary file 5 — Related Manuscript File. [file 41598_2020_58183_MOESM5_ESM.zip › XPS_7-3-2019_Stefan/XPS_7-3-2019_E4/4_ZnN2-0.4T_ITO_Etch/component.pdf]

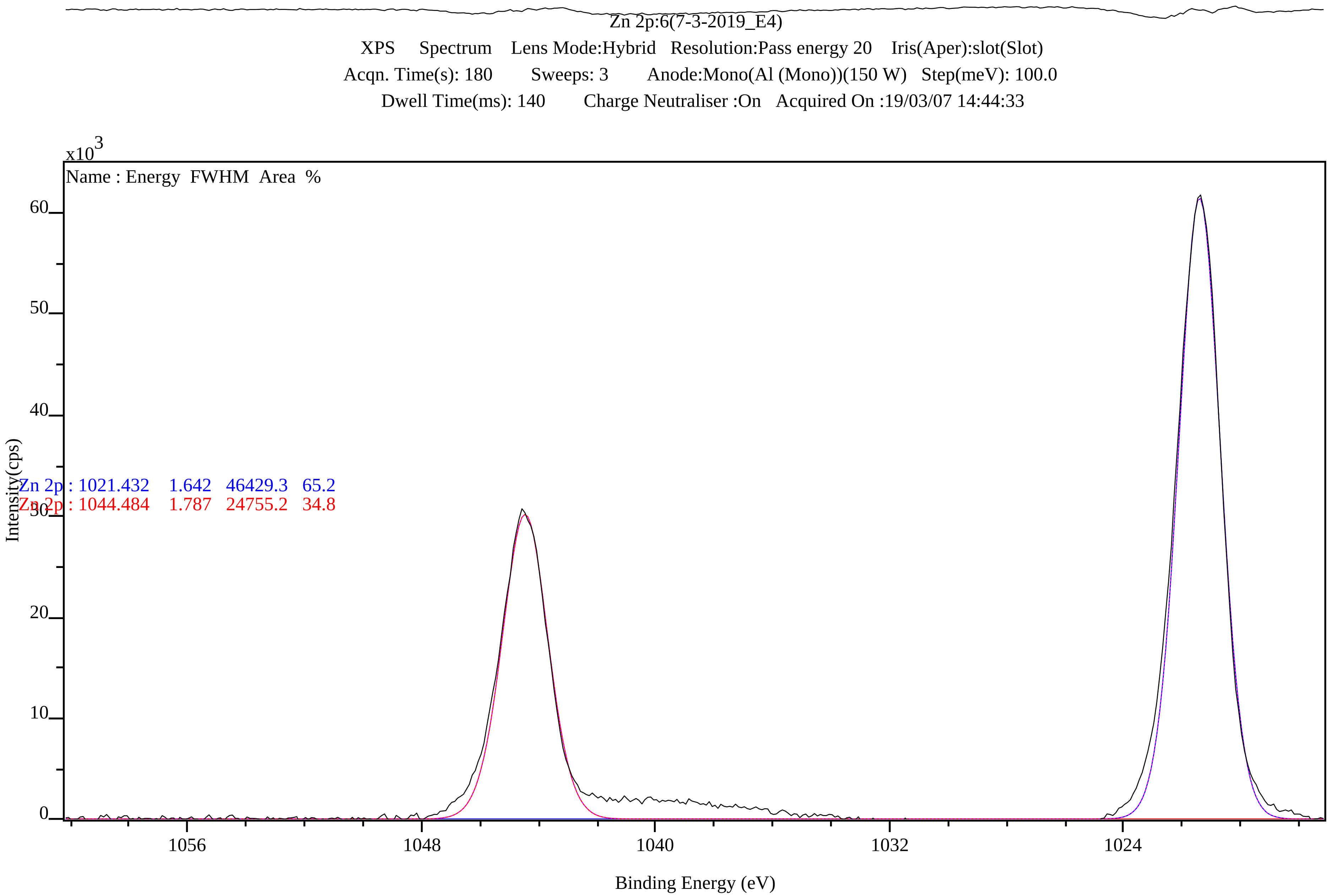

Zn 2p:6(7-3-2019_E4)
XPS Spectrum Lens Mode:Hybrid Resolution:Pass energy 20 Iris(Aper):slot(Slot)
Acqn. Time(s): 180 Sweeps: 3 Anode:Mono(Al (Mono))(150 W) Step(meV): 100.0
Dwell Time(ms): 140 Charge Neutraliser :On Acquired On :19/03/07 14:44:33
x10^3
Name : Energy FWHM Area %
Zn 2p : 1021.432 1.642 46429.3 65.2
Zn 2p : 1044.484 1.787 24755.2 34.8
Intensity(cps)
60
50
40
30
20
10
0
1056
1048
1040
1032
1024
Binding Energy (eV)

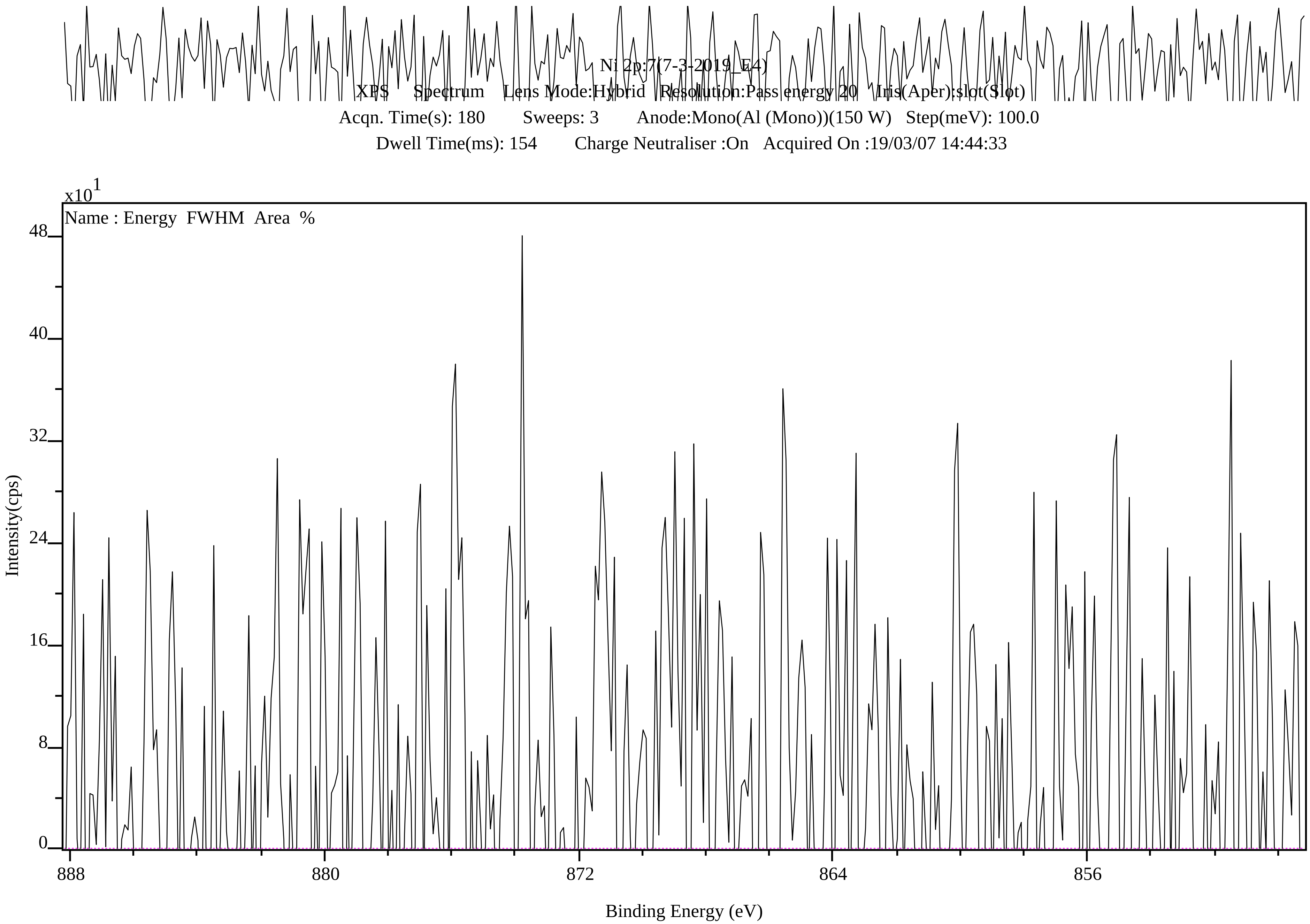

Ni 2p:7(7-3-2019_E4)
XPS Spectrum Lens Mode:Hybrid Resolution:Pass energy 20 Iris(Aper):slot(Slot)
Acqn. Time(s): 180 Sweeps: 3 Anode:Mono(Al (Mono))(150 W) Step(meV): 100.0
Dwell Time(ms): 154 Charge Neutraliser :On Acquired On :19/03/07 14:44:33
x10^1
Name : Energy FWHM Area %
48
40
32
24
16
8
0
Intensity(cps)
888
880
872
864
856
Binding Energy (eV)

O 1s:8(7-3-2019_E4)

XPS Spectrum Lens Mode:Hybrid Resolution:Pass energy 20 Iris(Aper):slot(Slot)

Acqn. Time(s): 181 Sweeps: 3 Anode:Mono(Al (Mono))(150 W) Step(meV): 100.0

Dwell Time(ms): 261 Charge Neutraliser :On Acquired On :19/03/07 14:44:33

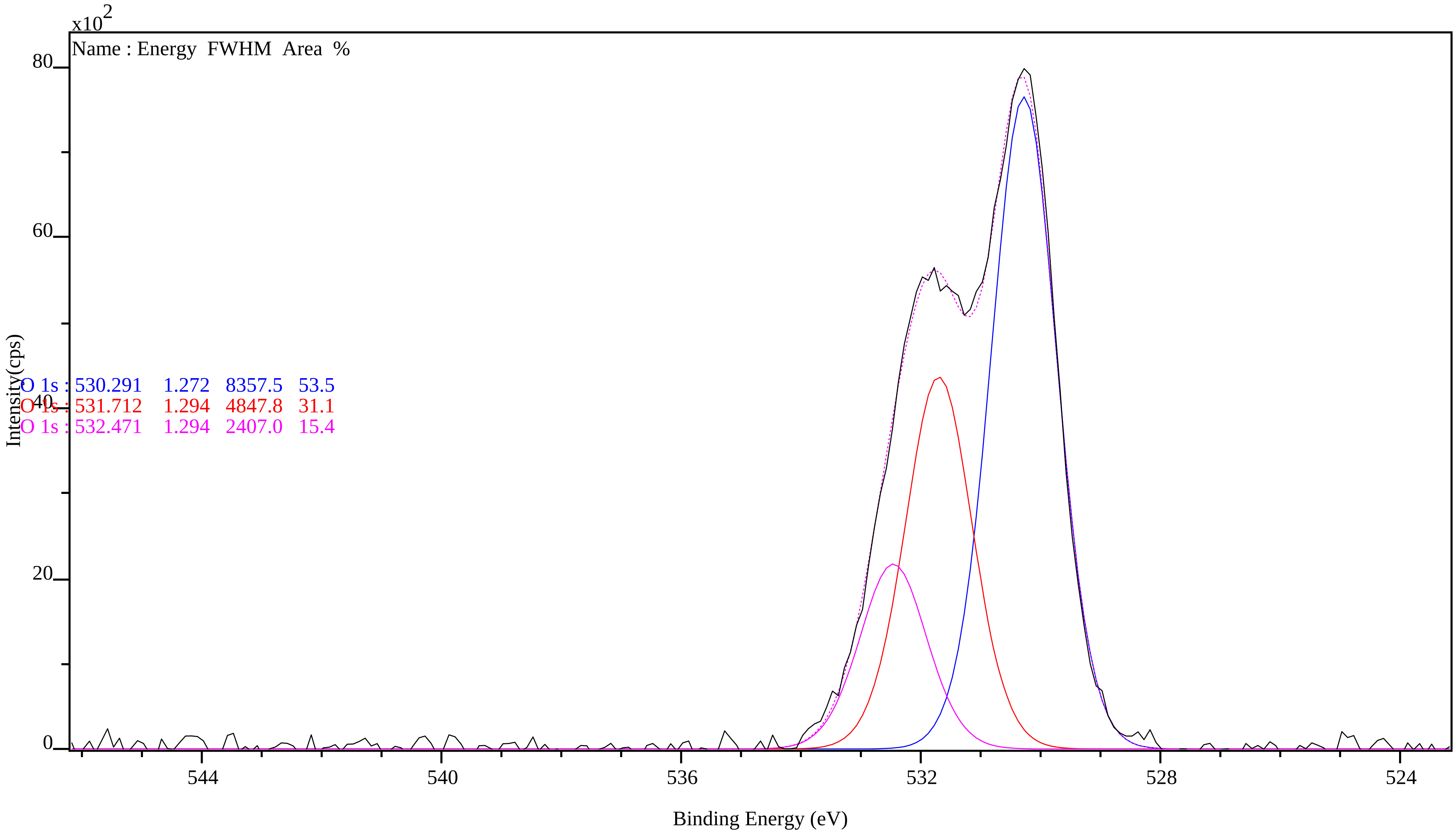

N 1s:9(7-3-2019_E4)

XPS Spectrum Lens Mode:Hybrid Resolution:Pass energy 20 Iris(Aper):slot(Slot)

Acqn. Time(s): 181 Sweeps: 3 Anode:Mono(Al (Mono))(150 W) Step(meV): 100.0

Dwell Time(ms): 286 Charge Neutraliser :On Acquired On :19/03/07 14:44:33

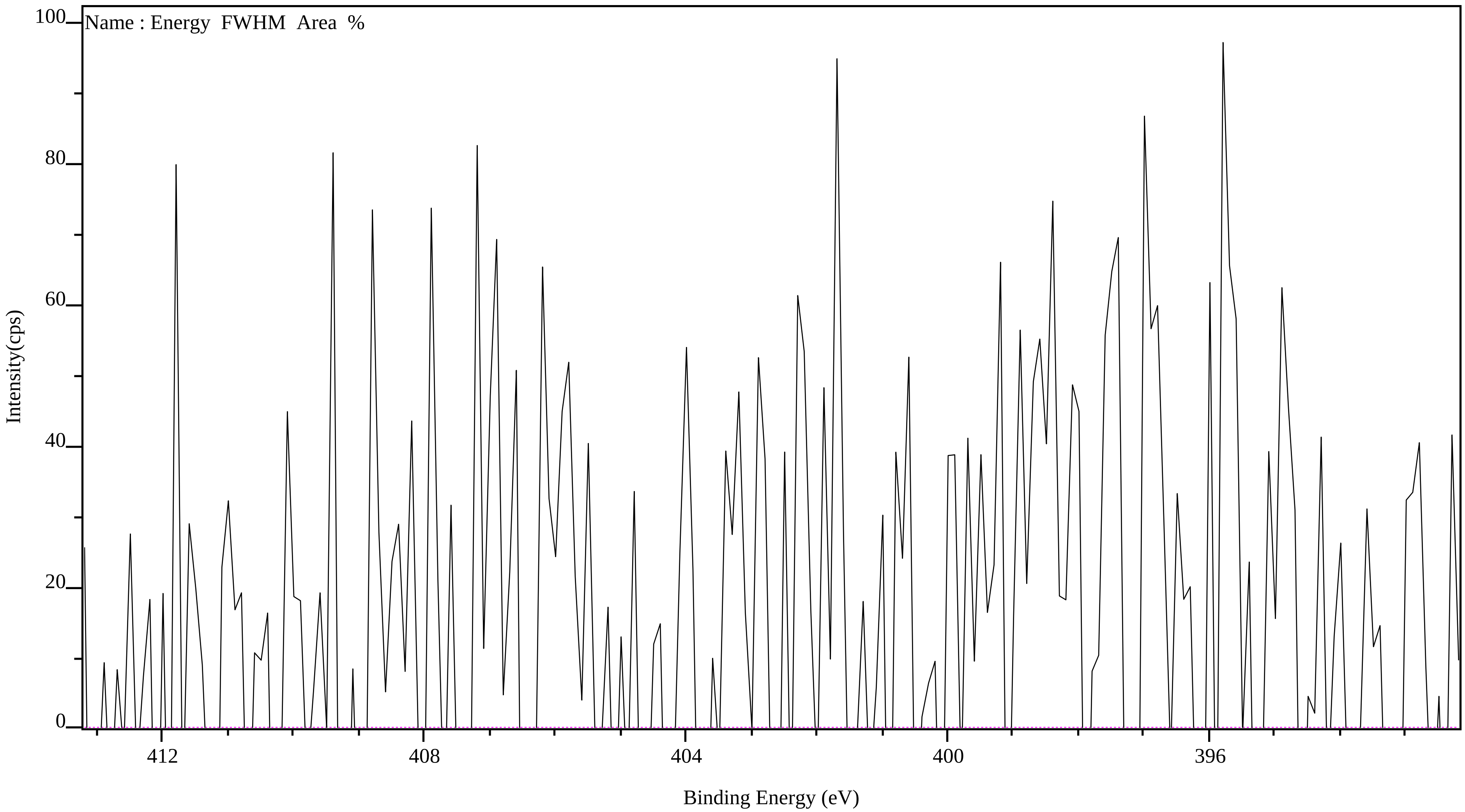

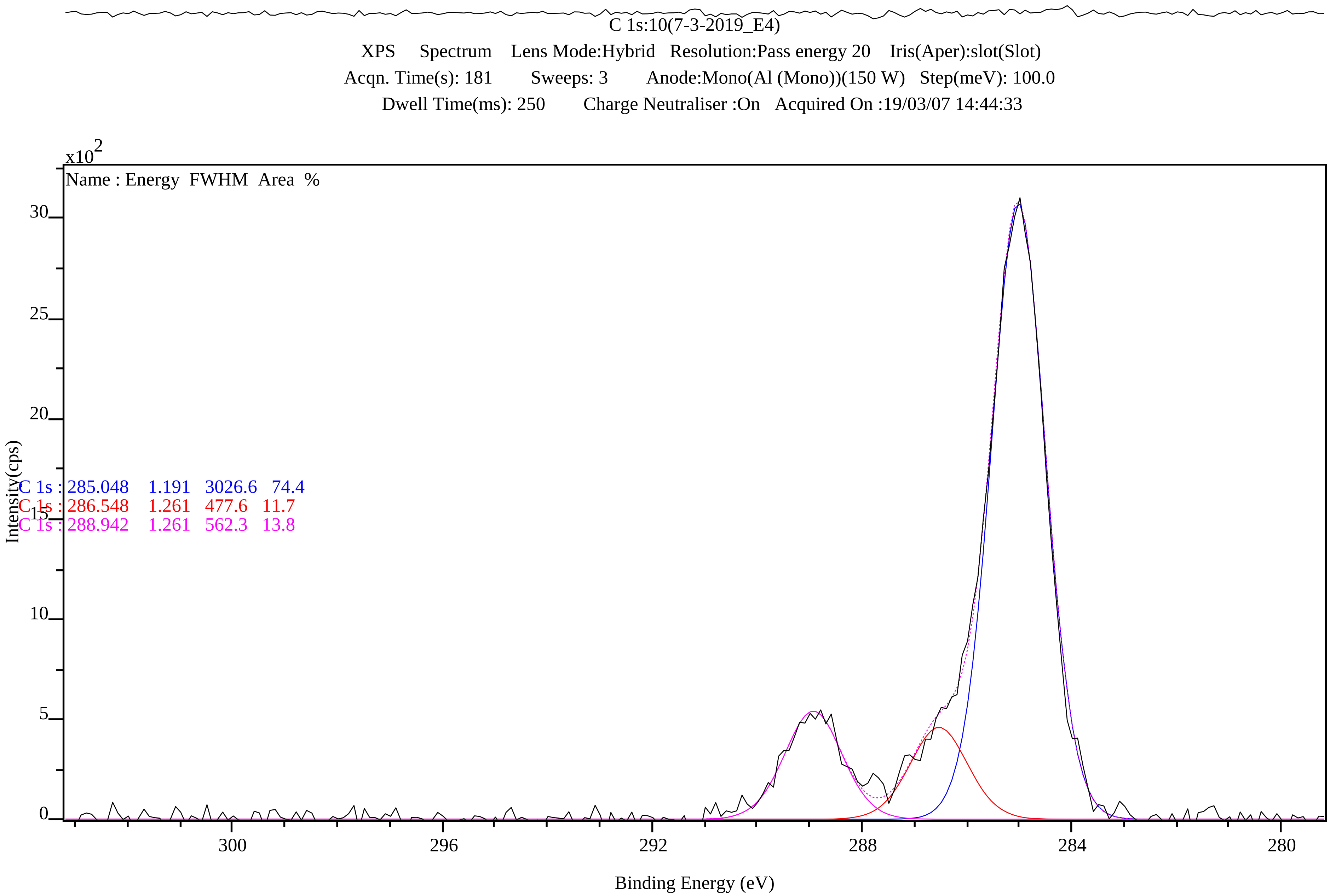

C 1s:10(7-3-2019_E4)
XPS Spectrum Lens Mode:Hybrid Resolution:Pass energy 20 Iris(Aper):slot(Slot)
Acqn. Time(s): 181 Sweeps: 3 Anode:Mono(Al (Mono))(150 W) Step(meV): 100.0
Dwell Time(ms): 250 Charge Neutraliser :On Acquired On :19/03/07 14:44:33
x10^2
Name : Energy FWHM Area %
C 1s : 285.048 1.191 3026.6 74.4
C 1s : 286.548 1.261 477.6 11.7
C 1s : 288.942 1.261 562.3 13.8
Intensity(cps)
30
25
20
15
10
5
0
300
296
292
288
284
280
Binding Energy (eV)
