# Supplementary material for: Influence of the magnetic field on bandgap and chemical composition of zinc thin films prepared by sparking discharge process
Source: Sci Rep. 2020 Jan 29;10:1388. doi: 10.1038/s41598-020-58183-4 (PMC6989455; doi:10.1038/s41598-020-58183-4)
Supplement: Supplementary file 5 — Related Manuscript File. [file 41598_2020_58183_MOESM5_ESM.zip › XPS_7-3-2019_Stefan/XPS_7-3-2019_E4/4_ZnN2-0.4T_ITO_Etch/survey.pdf]

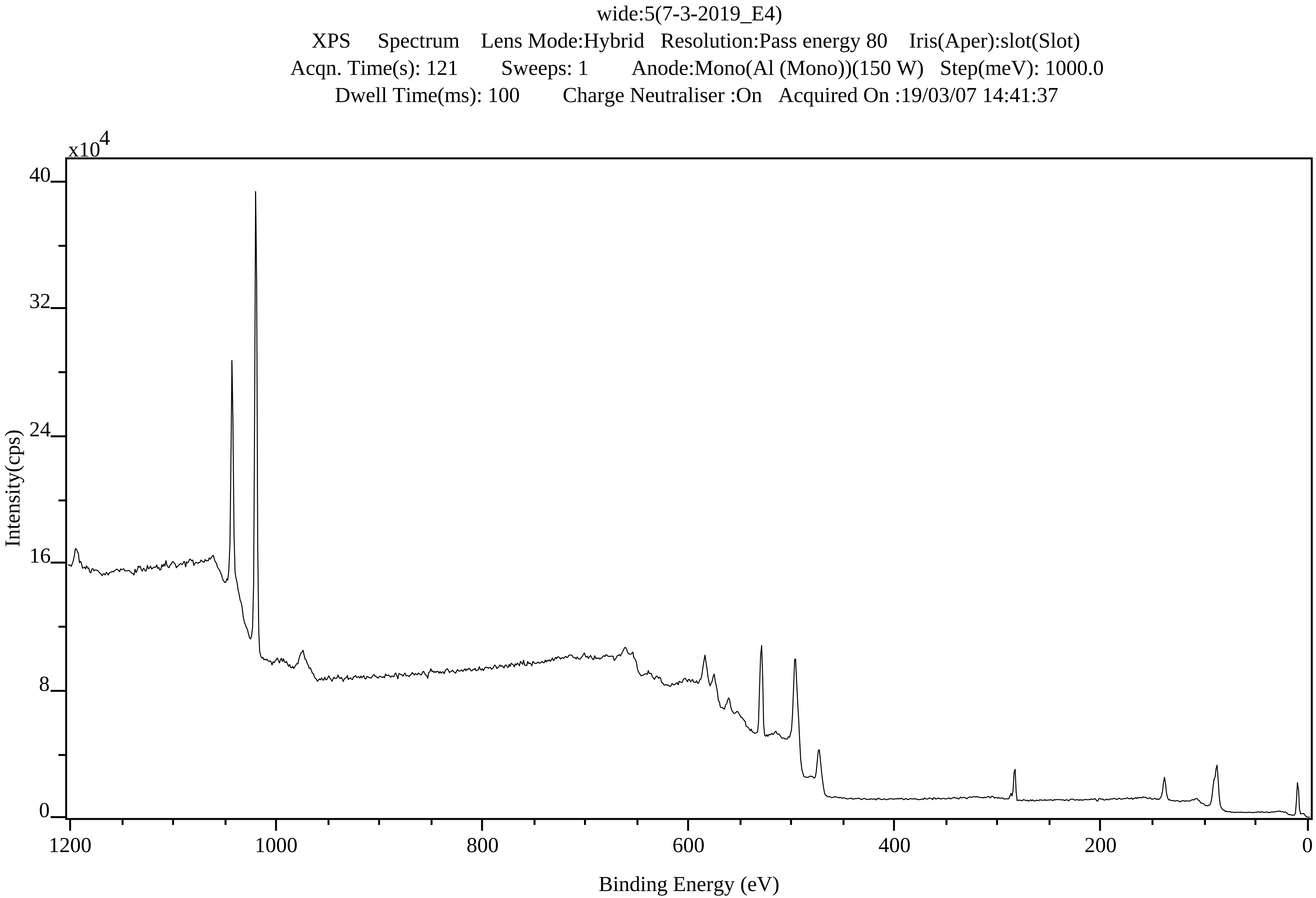
wide:5(7-3-2019_E4)
XPS Spectrum Lens Mode:Hybrid Resolution:Pass energy 80 Iris(Aper):slot(Slot)
Acqn. Time(s): 121 Sweeps: 1 Anode:Mono(Al (Mono))(150 W) Step(meV): 1000.0
Dwell Time(ms): 100 Charge Neutraliser :On Acquired On :19/03/07 14:41:37
x10^4
40
32
24
16
8
0
Intensity(cps)
1200
1000
800
600
400
200
0
Binding Energy (eV)
