# Supplementary material for: Influence of the magnetic field on bandgap and chemical composition of zinc thin films prepared by sparking discharge process
Source: Sci Rep. 2020 Jan 29;10:1388. doi: 10.1038/s41598-020-58183-4 (PMC6989455; doi:10.1038/s41598-020-58183-4)

Zn 2p:6(7-3-2019_E7)

XPS Spectrum Lens Mode:Hybrid Resolution:Pass energy 20 Iris(Aper):slot(Slot)

Acqn. Time(s): 180 Sweeps: 3 Anode:Mono(Al (Mono))(150 W) Step(meV): 100.0

Dwell Time(ms): 140 Charge Neutraliser :On Acquired On :19/03/07 14:16:48

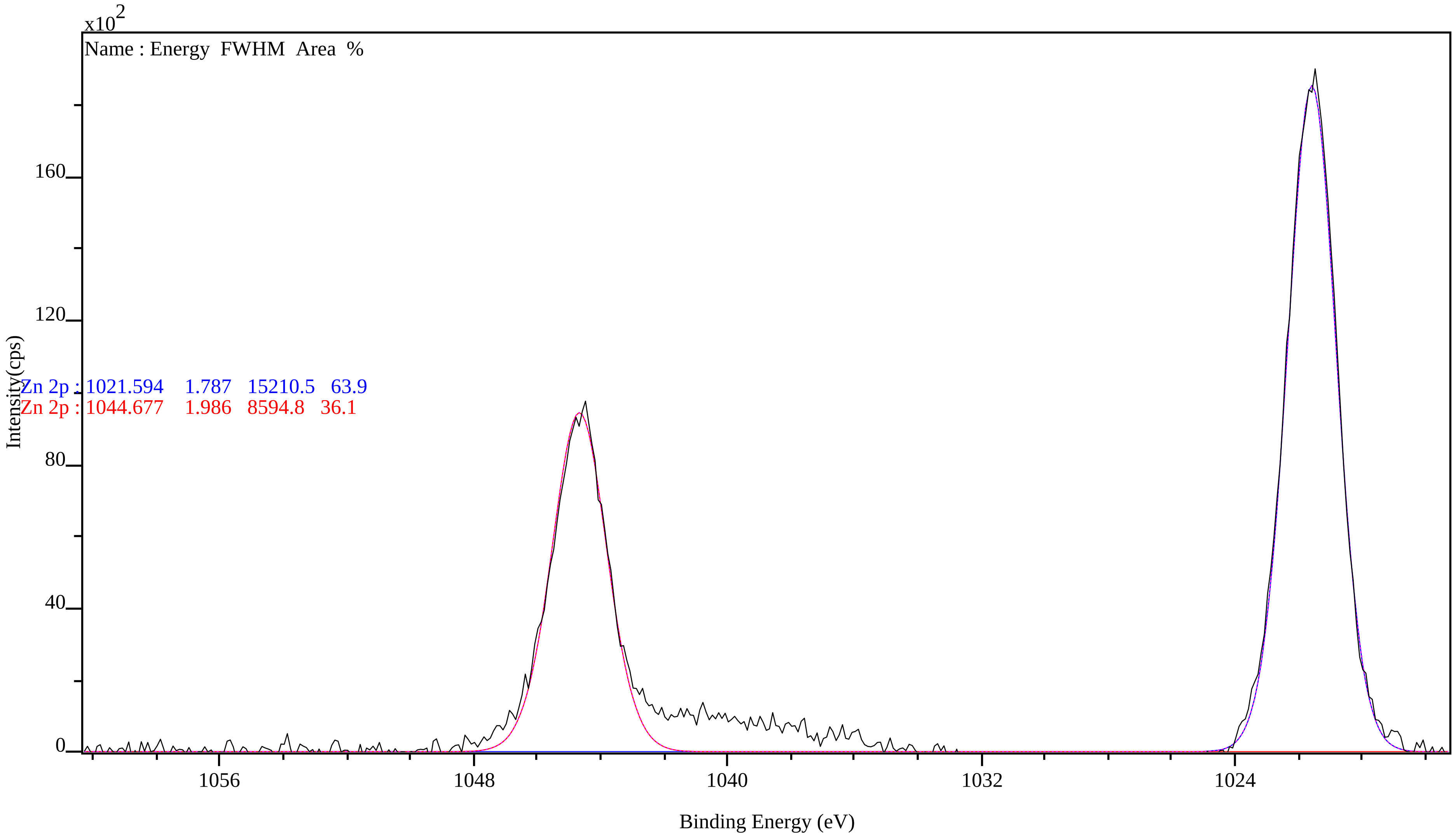

Ni 2p:7(7-3-2019_E7)

XPS Spectrum Lens Mode:Hybrid Resolution:Pass energy 20 Iris(Aper):slot(Slot)

Acqn. Time(s): 180 Sweeps: 3 Anode:Mono(Al (Mono))(150 W) Step(meV): 100.0

Dwell Time(ms): 154 Charge Neutraliser :On Acquired On :19/03/07 14:16:48

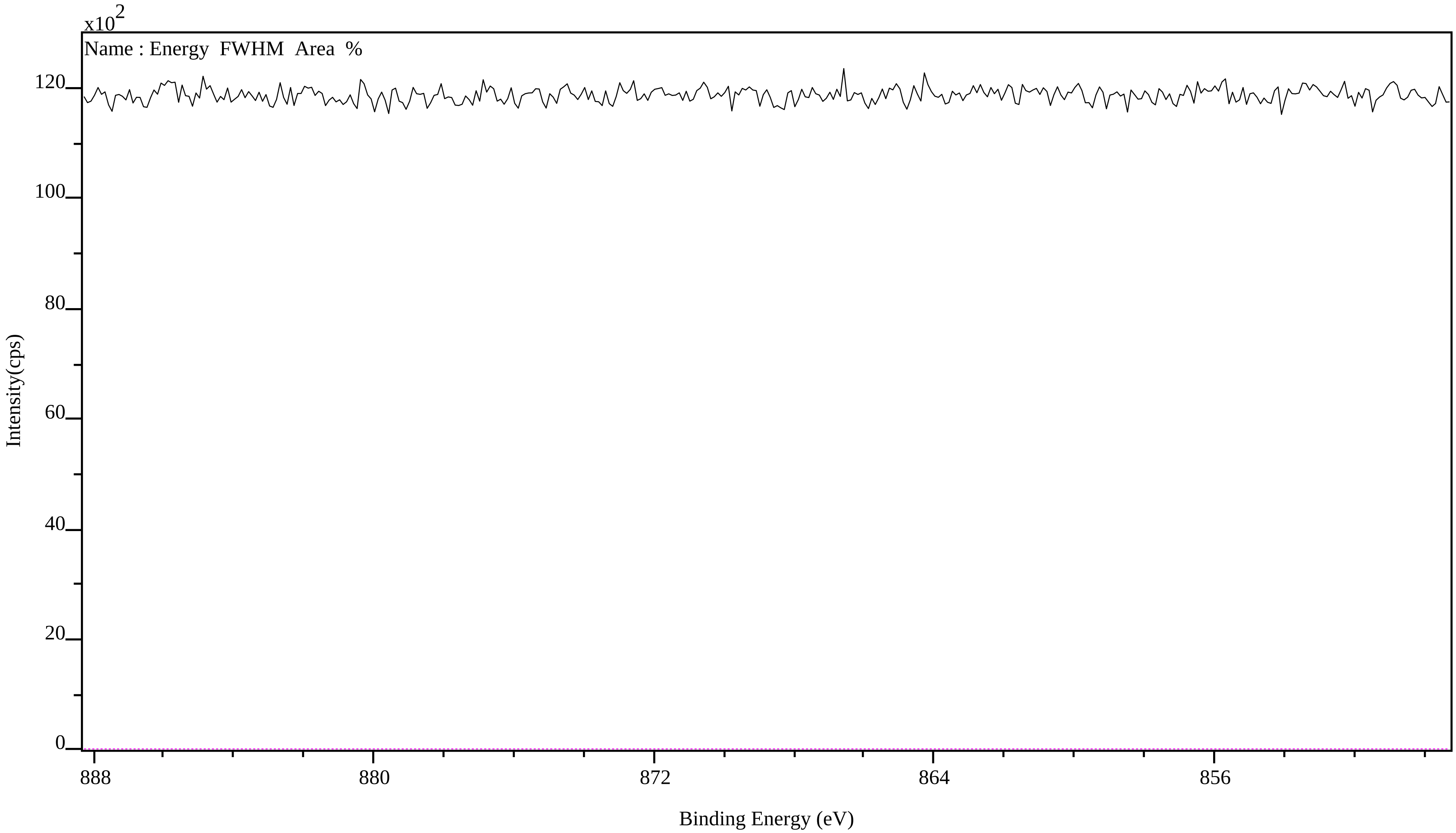

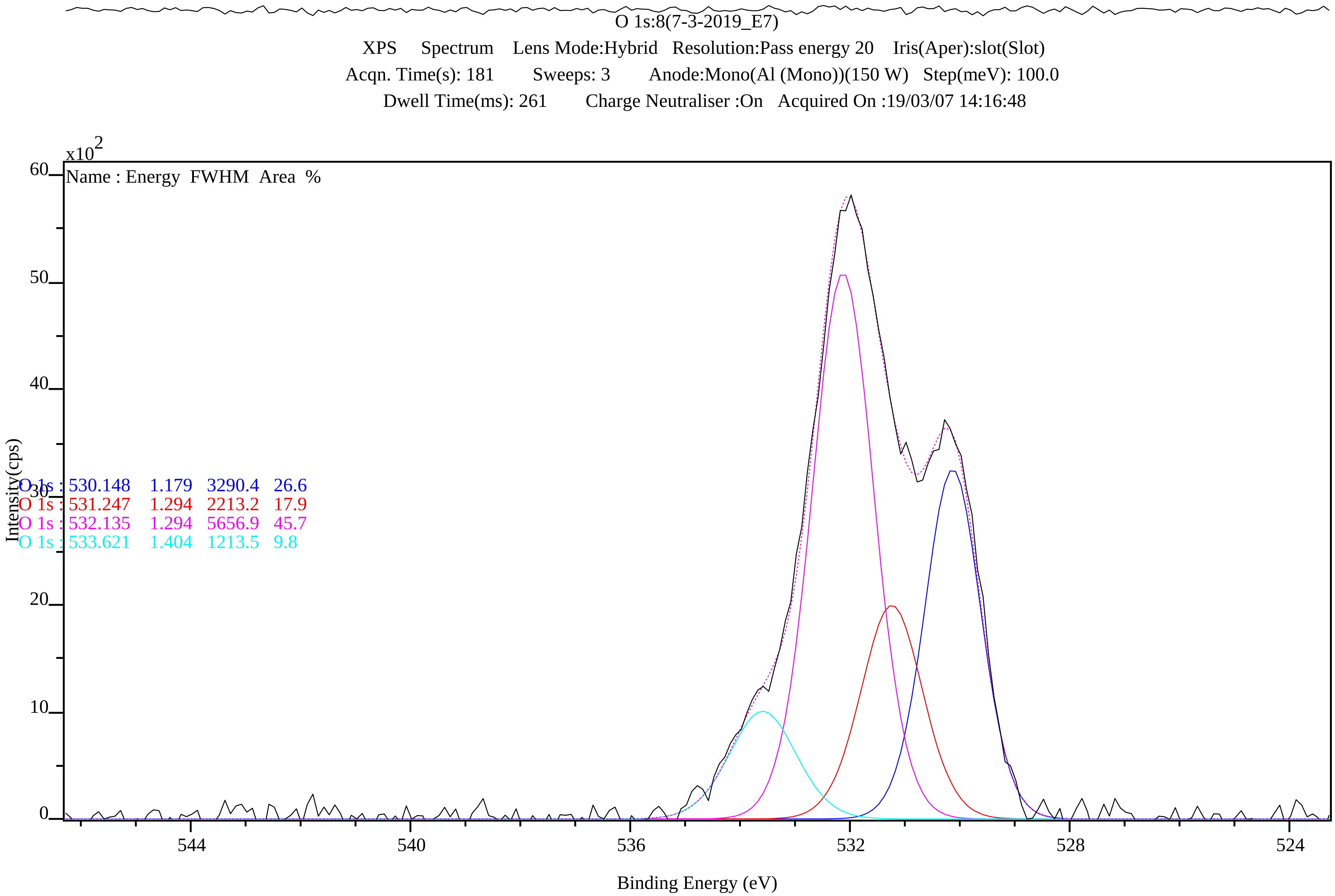
O 1s:8(7-3-2019_E7)
XPS Spectrum Lens Mode:Hybrid Resolution:Pass energy 20 Iris(Aper):slot(Slot)
Acqn. Time(s): 181 Sweeps: 3 Anode:Mono(Al (Mono))(150 W) Step(meV): 100.0
Dwell Time(ms): 261 Charge Neutraliser :On Acquired On :19/03/07 14:16:48
x10^2
Name : Energy FWHM Area %
O 1s : 530.148 1.179 3290.4 26.6
O 1s : 531.247 1.294 2213.2 17.9
O 1s : 532.135 1.294 5656.9 45.7
O 1s : 533.621 1.404 1213.5 9.8
Intensity(cps)
Binding Energy (eV)
0
10
20
30
40
50
60
544
540
536
532
528
524

N 1s:9(7-3-2019_E7)

XPS Spectrum Lens Mode:Hybrid Resolution:Pass energy 20 Iris(Aper):slot(Slot)

Acqn. Time(s): 181 Sweeps: 3 Anode:Mono(Al (Mono))(150 W) Step(meV): 100.0

Dwell Time(ms): 286 Charge Neutraliser :On Acquired On :19/03/07 14:16:48

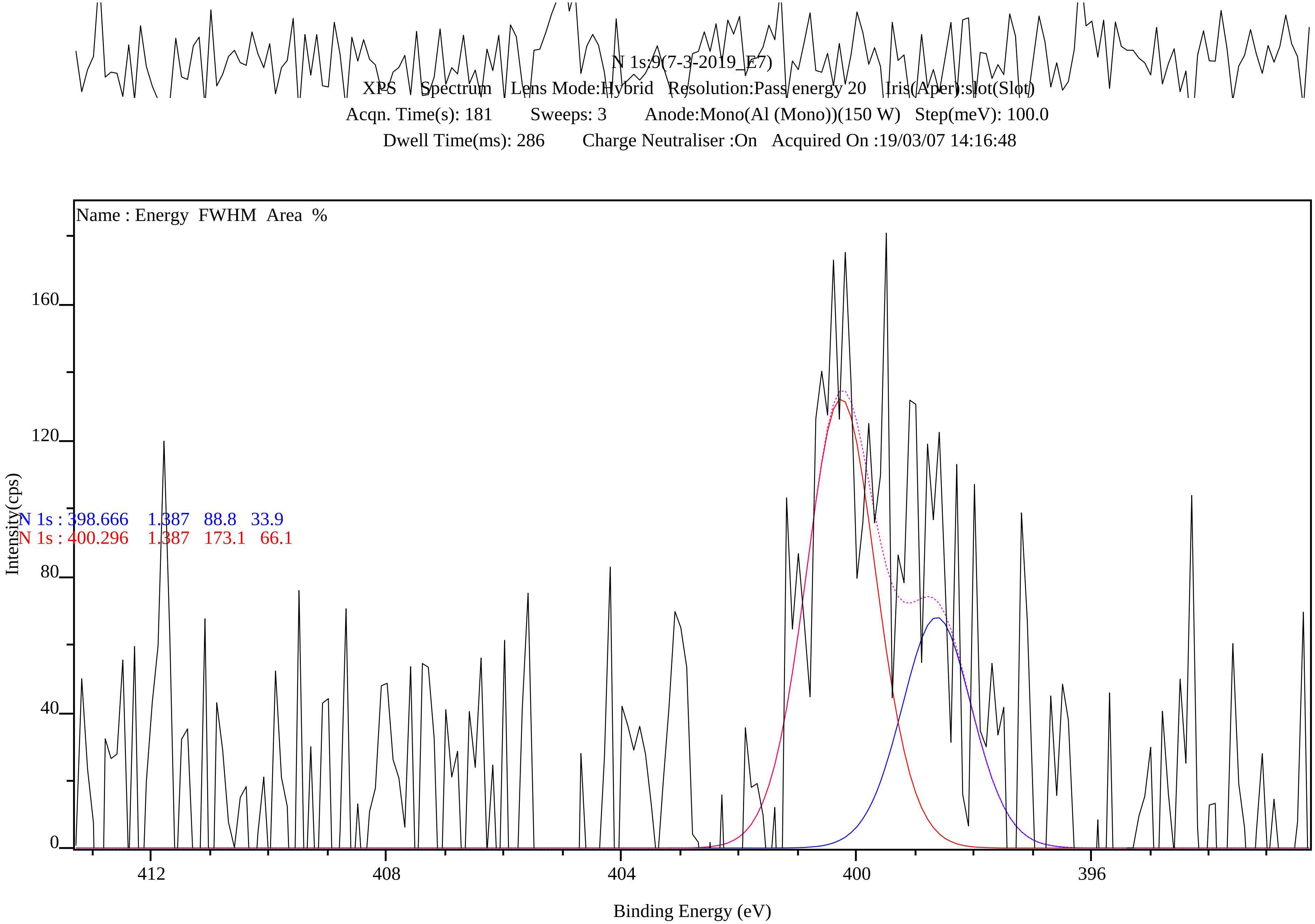

C 1s:10(7-3-2019_E7)

XPS Spectrum Lens Mode:Hybrid Resolution:Pass energy 20 Iris(Aper):slot(Slot)

Acqn. Time(s): 181 Sweeps: 3 Anode:Mono(Al (Mono))(150 W) Step(meV): 100.0

Dwell Time(ms): 250 Charge Neutraliser :On Acquired On :19/03/07 14:16:48

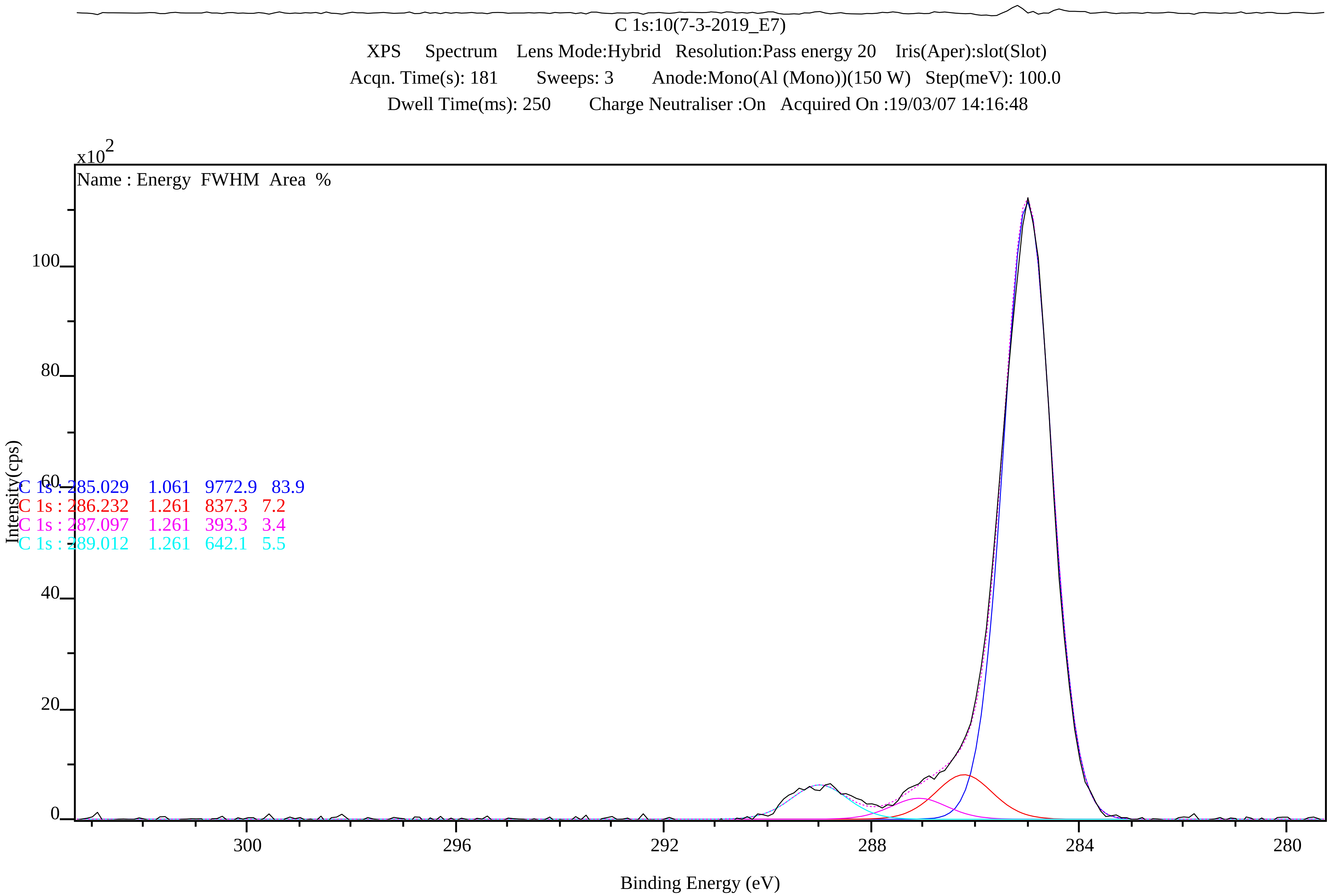

Supplement: Supplementary file 5 — Related Manuscript File. [file 41598_2020_58183_MOESM5_ESM.zip › XPS_7-3-2019_Stefan/XPS_7-3-2019_E7/7_ZnN2-0.2_Quartz_Etch/component.pdf]
