# Supplementary material for: Influence of the magnetic field on bandgap and chemical composition of zinc thin films prepared by sparking discharge process
Source: Sci Rep. 2020 Jan 29;10:1388. doi: 10.1038/s41598-020-58183-4 (PMC6989455; doi:10.1038/s41598-020-58183-4)

wide:5(7-3-2019_E7)

XPS Spectrum Lens Mode:Hybrid Resolution:Pass energy 80 Iris(Aper):slot(Slot)

Acqn. Time(s): 121 Sweeps: 1 Anode:Mono(Al (Mono))(150 W) Step(meV): 1000.0

Dwell Time(ms): 100 Charge Neutraliser :On Acquired On :19/03/07 14:14:11

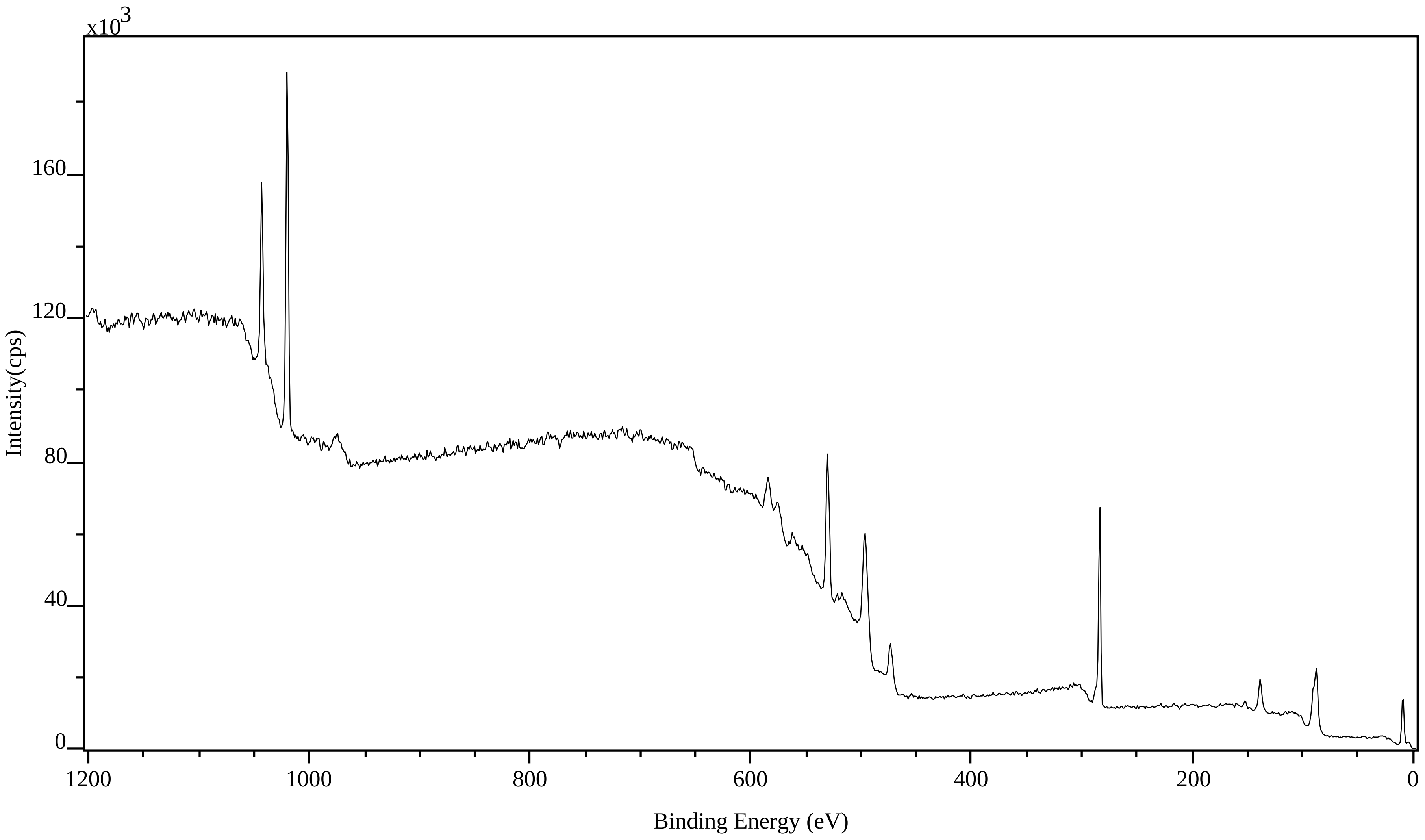

Supplement: Supplementary file 5 — Related Manuscript File. [file 41598_2020_58183_MOESM5_ESM.zip › XPS_7-3-2019_Stefan/XPS_7-3-2019_E7/7_ZnN2-0.2_Quartz_Etch/survey.pdf]
